# Antagonistic pleiotropy for carbon use is rare in new mutations

**DOI:** 10.1111/evo.13569

**Published:** 2018-09-13

**Authors:** Mrudula Sane, Joshua John Miranda, Deepa Agashe

**Affiliations:** ^1^ National Centre for Biological Sciences Tata Institute of Fundamental Research Bangalore India

**Keywords:** Antagonism, distribution of fitness effects, DFE, mutation accumulation, synergism, tradeoffs

## Abstract

Pleiotropic effects of mutations underlie diverse biological phenomena such as ageing and specialization. In particular, antagonistic pleiotropy (“AP”: when a mutation has opposite fitness effects in different environments) generates tradeoffs, which may constrain adaptation. Models of adaptation typically assume that AP is common ‐ especially among large‐effect mutations ‐ and that pleiotropic effect sizes are positively correlated. Empirical tests of these assumptions have focused on *de novo* beneficial mutations arising under strong selection. However, most mutations are actually deleterious or neutral, and may contribute to standing genetic variation that can subsequently drive adaptation. We quantified the incidence, nature, and effect size of pleiotropy for carbon utilization across 80 single mutations in *Escherichia coli* that arose under mutation accumulation (i.e., weak selection). Although ∼46% of the mutations were pleiotropic, only 11% showed AP; among beneficial mutations, only ∼4% showed AP. In some environments, AP was more common in large‐effect mutations; and AP effect sizes across environments were often negatively correlated. Thus, AP for carbon use is generally rare (especially among beneficial mutations); is not consistently enriched in large‐effect mutations; and often involves weakly deleterious antagonistic effects. Our unbiased quantification of mutational effects therefore suggests that antagonistic pleiotropy may be unlikely to cause maladaptive tradeoffs.

Biologists have long observed that organisms maximize resource allocation to one trait while compromising allocation to another trait (Lenoir 1984). Such tradeoffs manifest as negative correlations between traits, and may constrain evolution by limiting the breadth of phenotypes available to organisms (Rees 1993). The nature and strength of tradeoffs between traits can thus dictate whether organisms evolve to be generalists or specialists (Futuyma and Moreno 1988; Ferenci 2016). Tradeoffs also underlie diverse biological phenomena such as life‐history strategies (Zera and Harshman 2001; Sgrò and Hoffmann 2004), ageing (Kirkwood 2005), and assembly of microbial communities and host‐microbe interactions (Litchman et al. 2015). Although tradeoffs in resource use are undeniable, they remain relatively poorly understood at the mechanistic level. Tradeoffs can occur when multiple neutral or deleterious mutations accumulate and degrade traits under weak selection, leading to a negative correlation with other traits evolving under positive selection (Elena and Lenski 2003). For instance, in Lenski's long‐term experimental evolution lines, bacteria evolving under strong selection for one metabolic function (growth on glucose) lost multiple other metabolic functions because selection on these traits was very weak, allowing deleterious mutations to accumulate (Cooper 2014; Leiby and Marx 2014). Alternatively, tradeoffs may occur when a single mutation increases fitness in a specific environment (or trait), simultaneously reducing fitness in alternate environments (or a second trait) (Cooper and Lenski 2000). Such mutations are antagonistically pleiotropic for the two traits or environments, and the phenomenon is called antagonistic pleiotropy (henceforth “AP”).

The evolutionary impact of AP clearly depends on its incidence and magnitude. If AP is frequent or involves large‐effect mutations, the resulting tradeoffs are more likely to constrain adaptation. Historically, models of adaptive evolution have assumed that AP is the predominant form of pleiotropy (Lande 1983; Otto 2004), implying that synergistic pleiotropy (SP; when a mutation simultaneously either increases or decreases fitness in two different environments) is relatively uncommon. However, for single beneficial mutations in *Escherichia coli*, AP between fitness on glucose and alternate carbon sources was rare compared to positive SP (Ostrowski et al. 2005). Similarly, most of the first‐step beneficial mutations isolated from laboratory‐evolved *E. coli* populations showed SP, while only a few were strongly antagonistically pleiotropic (Dillon et al. 2016). Thus, contrary to model assumptions, empirical data suggests that AP may not be the predominant form of pleiotropy. A second assumption of theoretical models is that large effect mutations are more predisposed to show AP (Fisher 1930; Lande 1983), potentially explaining the prevalence of small effect mutations during adaptation in natural populations (Lande 1983; Orr and Coyne 1992; Tenaillon 2014; Dillon et al. 2016). Empirical studies have suggested that the degree of pleiotropy of genes or QTLs generally scales with their fitness effect sizes. However, these results have been questioned because most genes or QTLs only affected a small proportion of traits (Wagner et al. 2008; Wang et al. 2010; Dittmar et al. 2016), suggesting that the relationship between pleiotropy and fitness effect may be trait‐specific (Paaby and Rockman 2013). Interestingly, no empirical study has tested this assumption for individual mutations. Finally, the pleiotropic effect size of mutations is assumed to be proportional to their fitness effect in the selective environment where the mutation arose, that is its primary effect size (Orr 1992). Contrary to this assumption, previous studies found that the antagonistic effect size was not correlated with the primary effect size (Ostrowski et al. 2005; Dillon et al. 2016). Taken together, empirical studies indicate that SP is more common than AP, at least among beneficial mutations. Additionally, the direct and pleiotropic effects of beneficial mutations appear to be positively correlated when pleiotropy is synergistic, but not when pleiotropy is antagonistic. Thus, widely used models of adaptive evolution make assumptions that are either empirically untested or are poorly supported. Although the empirical studies mentioned above provide important results, all of them focus on beneficial mutations arising under strong directional selection, representing only a small fraction of all mutations. Most mutations are expected to be either neutral or mildly deleterious (Eyre‐Walker and Keightley 2007; Bataillon and Bailey 2014), but may accumulate under weak or fluctuating selection and drive subsequent adaptation (Barrett and Schluter 2008; Paaby and Rockman 2014; Gralka et al. 2016). Thus, by focusing only on beneficial mutations, we ignore most of the distribution of fitness effects of mutations (henceforth “DFE”), in turn ignoring the role of standing genetic variation in driving evolution.

To obtain unbiased estimates of AP, we evolved replicate populations of *E. coli* under mutation accumulation (henceforth “MA”) for hundreds of generations on a rich medium (Fig. 1). This regime of experimental evolution minimizes the strength of selection due to repeated bottlenecking of the populations, allowing all but lethal mutations to accumulate. We sequenced several time points frozen during experimental evolution to identify lines that had a single mutation relative to their immediate ancestor. Across 38 MA lines, we identified 80 isolates carrying new single mutations (including single nucleotide changes and small indels <10 bp; henceforth “mutants”) relative to their immediate ancestor. To determine the incidence of AP (i.e., the proportion of mutants that showed increased fitness on resource A and decreased fitness on resource B), we measured the growth rate of each of these mutants and their respective mutational ancestors on 11 different carbon sources. Many previous studies have demonstrated tradeoffs across these carbon sources (Cooper and Lenski 2000; Jasmin and Zeyl 2013; Leiby and Marx 2014; Satterwhite and Cooper 2015), indicating that they are sufficiently distinct environments where tradeoffs are frequent and relevant. For each pair of resources, we compared the observed incidence of AP with null distributions generated by randomly sampling from the independent DFEs for each resource (Fig. 1). We find that while pleiotropy is not rare among new mutations, AP is quite uncommon and variable across resources, even when compared to the null distribution. Although the incidence of AP often increases with the effect size of the mutation, the form of the relationship varies across resources. Finally, we find that the fitness effect sizes of mutations showing AP are either uncorrelated or negatively correlated. Taken together, our results suggest that AP is rarer than previously thought, indicating that AP‐mediated tradeoffs are generally unlikely to constrain adaptation.

**Figure 1:**
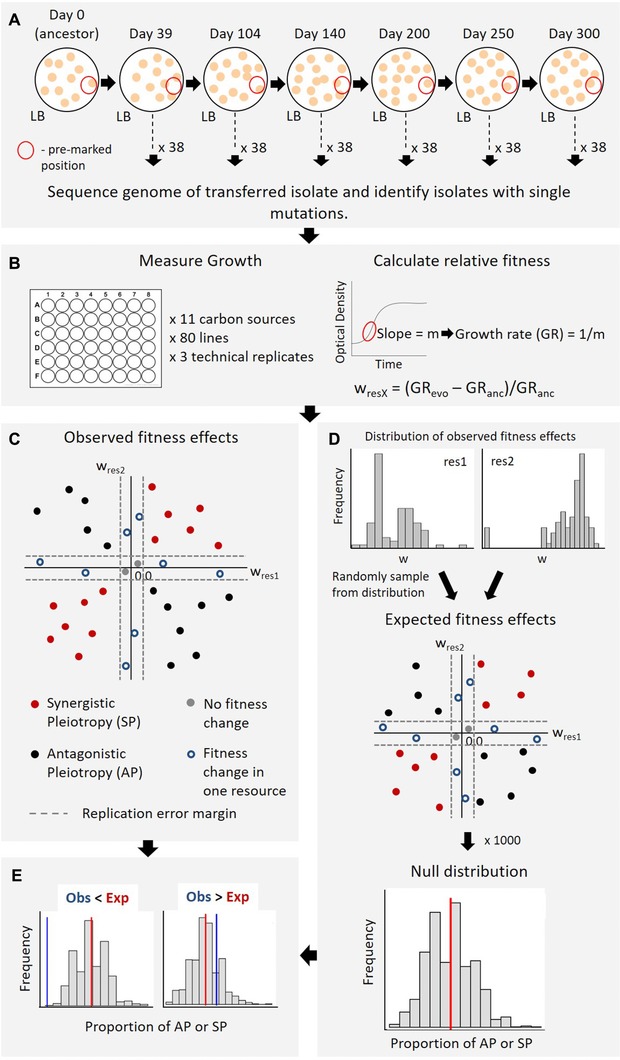
Experimental design and analysis. (A) Mutation accumulation experiment with wild‐type *E. coli*. (B) Estimating fitness effects of single step mutations in different environments. (C) Calculating the observed incidence of pleiotropic mutations. (D) Generating null distributions to calculate the expected incidence of pleiotropy. (E) Comparing the observed versus expected incidence of pleiotropy for each resource pair.

## Materials and Methods

### IDENTIFYING SINGLE‐STEP MUTATIONS IN MUTATION ACCUMULATION LINES

We obtained the wild‐type (WT) strain of *E. coli* K‐12 MG1655 from the Coli Genetic Stock Centre (CGSC, Yale University), streaked it on an LB (Miller, Difco) plate with 2% agar, and chose one colony at random as the WT ancestor for subsequent experiments. We then founded 38 WT MA lines (two lines per Petri plate) incubated at 37°C. For each line, every 24 hours we streaked out a random colony (closest to a premarked spot) on a fresh agar plate. Every 4–5 days, we inoculated a part of the transferred colony in LB broth at 37°C for 2–3 hours and froze 1 mL of the growing culture with 8% DMSO at –80°C. For the current study, we used stocks frozen on days 39, 104, 140, 200, 250, and 300 (Fig. 1A). For these time‐points, we sequenced whole genomes on the Illumina Hi‐seq 2500 platform (see SI Methods for details). We aligned quality‐filtered reads to the NCBI reference *E. coli* K‐12 MG1655 genome (RefSeq accession ID GCA_000005845.2) and called mutations (single nucleotide changes and short indels <10 bp; see SI Methods for details). At the final sequenced time point of the MA lines, each line had several mutations (Table S1), with an average of ∼7 mutations per line. From our sequencing data, we identified a total of 80 isolates carrying a single mutation with respect to their immediate ancestor (“mutants”; Table S1 and Table S2). For instance, if an MA line had one mutation on day 39 and an additional mutation at day 200, we retained both these isolates for further analysis, but discarded intermediate isolates (from days 104 and 140) since they did not represent single mutational steps. In this case, we obtained two distinct single‐mutation steps from a single MA line: for the evolved isolate at day 39, we considered the WT as ancestor; and for the evolved isolate at day 200, we considered the evolved isolate at day 39 as the ancestor. Of the 38 lines, five did not have any single‐mutation steps and were excluded from further analysis; the remaining 33 lines had acquired 1–4 single‐mutation steps (Table S1).

### ESTIMATING FITNESS EFFECT SIZES AND CALCULATING OBSERVED AND NULL ESTIMATES OF AP AND SP

For each mutant and its respective ancestor, we measured growth rates (as a fitness proxy) in liquid culture: LB broth (Miller, Difco), or M9 minimal salts medium + 5 mM of a carbon source (glucose, trehalose, fructose, maltose, lactose, galactose, succinate, pyruvate, melibiose, malate, fumarate; Sigma‐Aldrich; see S1 Methods for details). For a subset of 40 mutants, we repeated growth rate measurements in glucose, galactose, and pyruvate to ensure that growth rates were consistent across independent runs (Fig. S1). We used the average growth rate of three technical replicates of each mutant to calculate relative fitness as: (Growth rate of mutant – Growth rate of ancestor)/Growth rate of ancestor (Fig. 1B). A negative value indicated a deleterious mutation, while a positive value indicated a beneficial mutation. Growth rates for WT measured in different plates run on different days varied by less than 5%. Similarly, the error in measurement of growth rates across technical replicates (run on the same day) was also less than 5%. Hence, we considered mutants with <5% change in fitness from the ancestor as showing no change. For each pair of carbon sources, we calculated the proportion of mutants showing evidence of AP (relative growth rate < –0.05 in carbon source A but relative growth rate >0.05 in carbon source B) or SP (relative growth rate < –0.05 in both carbon source A and carbon source B as synergistic decreases in fitness; relative growth rate >0.05 in carbon source A and B as synergistic increases in fitness) (Fig. 1C). To determine the proportion of comparisons showing AP or SP for each focal resource, we calculated the total number of mutants showing AP or SP across all pairwise combinations with the focal resource. Since there were 80 mutants and 10 possible resource pairs for each focal resource, there were a total of 800 comparisons per focal resource. Thus, we calculated the “observed” proportion of comparisons showing AP or SP for each focal resource as the number of mutants showing AP or SP, divided by 800.

For each of the 55 possible resource combinations, we generated a null distribution of the incidence of pleiotropy among all mutations. We randomly picked a fitness value from the observed distribution of fitness effects (DFE) for resource A, simultaneously picking a fitness value from the DFE for resource B. We picked 80 such pairs of fitness values (sampling with replacement), and calculated the proportion of pairs showing AP or SP. We performed 1000 iterations to generate a null distribution of the incidence of AP or SP for each resource pair (Fig. 1D). When considering only beneficial mutations, we generated two null distributions for each resource pair (total 110 null distributions), since a beneficial mutation could occur in either resource A or B (Fig. 1D). For each null distribution, we estimated the average proportion of AP (or SP) as the “expected” incidence of AP (or SP), for comparison with the observed incidence of AP (or SP) for the specific resource pair (Fig. 1E).

### TESTING FOR A CORRELATION BETWEEN THE INCIDENCE OF PLEIOTROPY AND FITNESS EFFECT SIZE

For isolates showing AP or SP, we calculated the magnitude of pleiotropic effect size as the absolute values of relative fitness in each resource within a pair. We calculated the correlation between fitness effect size and proportion of pleiotropy in two ways. (1) We categorized the magnitude of fitness for each focal resource into four arbitrary classes: very low (relative fitness 0.05–0.1), low (relative fitness 0.1–0.2), medium (relative fitness 0.2–0.3), and high (relative fitness 0.3–0.4) (see SI Methods for details about binning of fitness effects). We then counted the number of instances of pleiotropy (AP or SP) in each class and tested whether the proportion of pleiotropy was correlated with the magnitude of fitness effect. (2) We selected only those mutants that showed pleiotropy (AP or SP) for a given focal resource. We then classified them into the four fitness effect bins, and counted the number of mutants falling in each class. Using these data, we asked: conditional on the occurrence of pleiotropy, how is it distributed across fitness effect size classes? Similarly, to calculate the null expectation for the relationship between fitness effect size and proportion of pleiotropy, we binned, as described above, fitness values randomly drawn from the DFEs for individual resources. We measured the proportion of pleiotropy (AP or SP) within the null distribution and asked if it was correlated with the fitness effects for each of the 55 resource pairs.

### TESTING FOR A CORRELATION BETWEEN PRIMARY AND PLEIOTROPIC FITNESS EFFECT SIZES

For each resource pair, we computed the Spearman's rank correlation between the magnitudes of effect sizes (absolute values of relative fitness, as above) in the two resources, for all mutants that showed pleiotropy (AP or SP). We included fitness data for LB, since our MA lines evolved in this medium. Thus, for this analysis, we had 12 resources and 66 resource pairs. We excluded resource pairs for which <5 mutants showed the specific type of pleiotropy. Since AP is rare, we could compute effect size correlations for only 50 of 66 resource pairs.

## Results

### ANTAGONISTIC PLEIOTROPY IS RARE, AND VARIES ACROSS ENVIRONMENTS

**Figure 2:**
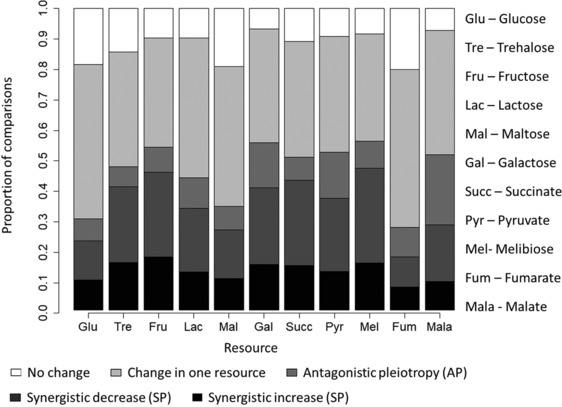
Incidence of pleiotropy among single mutational steps. Stacked bar plot shows the observed proportion of mutants showing various categories of fitness effects. Each bar represents pooled data across all pairwise resource comparisons for a focal resource (80 mutants × 10 resource pairs involving the focal resource = 800 data points per bar).

To estimate the incidence of pleiotropy, we measured the fitness effect (relative growth rate) of single mutations obtained during an MA experiment, on 11 different carbon sources (Fig. 1). As expected, the distribution of fitness effects (DFEs) observed for each resource showed that on average, ∼49% of all sampled mutations were deleterious, and would have been missed if we focused only on beneficial mutations (Fig. S2). Mutants differed in their fitness effects across carbon sources (Fig. S3), suggesting that single mutations could impact fitness in multiple environments. Combining data across all mutants and resource pairs (80 mutants × 55 resource pairs = 4400 data points), we observed pleiotropy in ∼46% of the cases (Fig. 2; also see Fig. S4 and Fig. S5). However, most pleiotropic mutations were synergistic (SP, ∼35% of total) rather than antagonistic (AP, ∼11%). Importantly, resource identity had a significant impact on the incidence of both AP and SP (Fig. 2; *P* < 0.05, generalized linear model with binomial errors; Table S3 and Table S4; also see Table S5 and Table S6 for all pairwise resource comparisons). Malate had the highest incidence of AP (∼23%) (Fig. S6A), while melibiose showed the highest incidence of SP (50%) (Fig. S6B). Finally, AP was even more rare (∼4%) when considering only beneficial mutations, whereas SP was not as rare (∼13% of beneficial mutations). Overall, AP was relatively rare compared to SP.

All of our single‐mutation steps occurred on different genetic backgrounds with distinct “ancestral” fitness. Therefore, we asked whether the incidence of AP changed across consecutive mutational steps. While most of our evolved MA lines had more than one mutation (Table S1), very few lines had more than two single‐mutation steps (Fig. S7). Hence, we compared the incidence of AP in first‐step and second‐step mutations. We found that in 9 out of 11 resources, first‐step and second‐step mutations had similar incidence of AP (*P* > 0.05, generalized linear model with binomial errors, Table S7; Fig. S7). The two exceptions to this pattern showed opposite results: for AP involving fumarate, second‐step mutations were less likely to show AP; whereas for malate, second‐step mutations were more likely to show AP (Fig. S7). Thus, these results suggest that in the initial phase of mutation accumulation, the incidence of AP is not affected significantly by the genetic background or ancestral fitness.

Another way to quantify the incidence of pleiotropy is to ask whether a given mutation shows pleiotropy across multiple resource pairs. Most mutations (72 of 80) showed AP for at least one pair of resources, with a median of six and a range of 0–24 resource pairs (out of 55 total resource pairs; Fig. S8). In contrast, all mutants showed SP for at least one resource pair, with a median of 16 resource pairs (Fig. S8). These results again highlight the relative rarity of AP compared to SP. The relatively high frequency of SP suggests that the paucity of AP cannot be explained by a general inability to simultaneously detect small, pleiotropic fitness effects in multiple environments. To test whether mutations in genes with specific functions are more likely to show AP, we classified antagonistically pleiotropic mutations based on the Gene Ontology (GO) terms associated with the affected gene (Table S2). We focused on mutations that cause AP in up to five resource pairs or between 5 and 10 resource pairs, since very few mutations caused AP in >10 resource pairs. We found that the distribution of molecular function categories in both categories were comparable to the null expectation from the number of *E. coli* genes with each GO term (*P* > 0.05, chi‐squared test, Table S8; also see Fig. S9). Thus, antagonistically pleiotropic mutations were not significantly enriched for specific functions.

Finally, we compared the observed incidence of AP and SP with the null expectation derived from DFEs for each resource in a given resource pair combination (Fig. 1C–E). Using random, repeated sampling from observed DFEs for each resource pair, we estimated that the expected incidence of AP was ∼16% (average across all resource pairs; Fig. S11); this is greater than the observed incidence of ∼11% described above. For each resource pair, we tested whether the observed proportion of mutants showing AP was significantly greater or lower than expected from the null distribution for the specific resource pair. We found that for most resource pairs (39 of 55), significantly fewer mutations showed AP than expected by chance (Table 1; Fig. S11). In contrast, in most cases SP was observed significantly more often than expected (46 of 55 resource pairs; Table 1; Fig. S12). When we considered only beneficial mutations for each focal resource, the pattern for AP was even more stark, with all 110 resource pairs showing lower AP incidence (on average, ∼4% across all resource pairs) than expected (average ∼40% across all resource pairs) (Table 1; see also Fig. S13). However, for beneficial mutations, SP showed a reverse pattern than for all mutations, with 109 of 110 resource pairs showing less SP (∼13% across all resource pairs) than expected (∼26% across all resource pairs) (Table 1; see also Fig. S14). Together, these results reinforce our conclusion that AP is very rare in new mutations. In contrast, SP is more common than expected, except when considering only beneficial mutations. Overall, our results may explain why AP‐mediated tradeoffs have been difficult to uncover in empirical studies: AP is not only rare, but also depends on the environment.

**Table 1:** Summary of the number of resource pairs that show specific patterns of observed versus expected incidence of pleiotropy for all mutations, or only for beneficial mutations

Number of resource pairs showing:	All mutations	Beneficial mutations
**Antagonistic pleiotropy (AP)**		
Obs > Exp	14	0
Obs < Exp	39	110
Obs = Exp	2	0
**Synergistic pleiotropy (SP)**		
Obs > Exp	46	0
Obs < Exp	9	109
Obs = Exp	0	1

Observed and expected proportions of AP or SP were designated as significantly different (Obs > Exp or Obs < Exp) when *P* < 0.05 for a Student's *t*‐test comparing the observed proportion of AP or SP with the mean of the null distribution for each resource pair (see Fig. 1C–E). Null distributions of the incidence of AP and SP are shown in Figs. S11–S14.

### LARGE‐EFFECT MUTATIONS ARE MORE LIKELY TO SHOW PLEIOTROPY

Theoretical models of adaptation assume that large‐effect mutations are more commonly associated with pleiotropic effects, and that these pleiotropic effects are mostly deleterious. To test this assumption (?), for each focal resource we grouped fitness effect sizes into four arbitrary classes: very low (relative fitness 0.05–0.1), low (relative fitness 0.1–0.2), medium (relative fitness 0.2–0.3), and high (relative fitness 0.3–0.4). Across all resources, ∼37, 45, 14, and 4% of fitness effects were classified in the respective classes. We then tested the relationship between the incidence of AP and fitness effect size in two ways.

**Figure 3:**
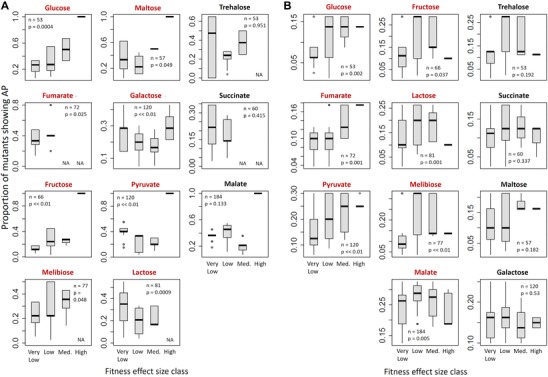
Relationship between the incidence of AP and fitness effect size. (A) Proportion of mutants showing AP in each fitness effect size class. We classified all fitness values for a focal resource into four effect size classes. Boxplots show the proportion of values in a class representing AP with any other resource (*n* = total instances of AP). (B) Distribution of antagonistically pleiotropic mutations across fitness effect size classes. We chose all instances of AP in our dataset (total 943), of which *n* (indicated in each plot) involved a given focal resource. For each resource, we calculated the proportion of measurements belonging to each effect size class. In both panels, plot titles in red indicate a significant correlation between the fitness effect size class and incidence of AP (*P* < 0.05, Kendall's rank correlation; see *P* values in each panel; also see Table S9 and Table S10). In panel A, “NA” indicates a lack of mutations in the respective fitness class. For correlations between expected AP incidence (based on null distributions) and fitness effect size, see Fig. S15.

We first asked: in each of the four fitness effect size classes, what proportion of mutants show AP? Considering each focal resource in turn, we observed distinct relationships between the proportion of AP and the mutational effect size. Four resources showed the predicted, monotonic positive correlation (Kendall's rank correlation, *P* < 0.05; first column in Fig. 3A; Table S9); three resources showed a concave positive relationship (second column in Fig. 3A); lactose showed a significant negative correlation; and the remaining three resources did not show a significant correlation between the incidence of AP and the fitness effect size. The correlation patterns for seven of 11 resources supported the prediction that large‐effect mutations are more likely to show AP; but the form of this relationship was not consistent across resources. Since a large fraction of mutations (37%) fall within the smallest effect size class, the relatively low incidence of AP in this bin is consistent with the conclusion that AP is generally rare. For SP, we observed more consistent relationships: the incidence of SP was positively correlated with effect size class for 10 of 11 focal resources (Fig. S10, Table S12).

Next, we asked: conditional on the occurrence of AP, do antagonistically pleiotropic mutations occur more frequently in large effect size classes? We again found variable patterns across resources: three resources showed a monotonic or saturating increase (first column, Fig. 3B); four resources showed a convex relationship with highest AP incidence at intermediate fitness effect sizes (second column, Fig. 3B); and the remaining four resources showed no correlation (Table S10). In contrast, for datasets generated from randomly sampling DFEs for each resource, we found that effect sizes were consistently negatively correlated with the proportion of AP (Fig. S15; Table S11). Thus, the observed positive relationship between proportion of AP and effect size cannot be explained by a greater chance of detecting AP in large‐effect mutations. A similar analysis for SP showed that four of 11 resources showed a positive correlation between effect size and incidence of SP (Fig. S10; Table S13), compared to the null expectation of a consistently negative correlation (Fig. S16, Table S14). Thus, while the incidence of AP in observed mutations is often positively correlated with the fitness effect size of those mutations, this pattern is not generally true for SP.

Together, these results offer partial support for the prediction that large‐effect mutations may be more like to show AP, with the caveat that the results vary dramatically across environments. For AP involving glucose, we observed a consistent, strong positive correlation in both analyses (compare Fig. 3A and Fig. 3B), indicating that AP‐mediated tradeoffs for glucose are more likely to occur for large‐effect mutations. However, for other resources, the relationship between effect size and AP incidence is either inconsistent, or insignificant, or more complex with intermediate maxima or minima. Hence, with respect to the model assumption, this relationship is not robust and requires more careful attention.

### PRIMARY FITNESS EFFECTS SIZES ARE CORRELATED WITH SYNERGISTIC, BUT NOT ANTAGONISTIC EFFECT SIZES

**Figure 4:**
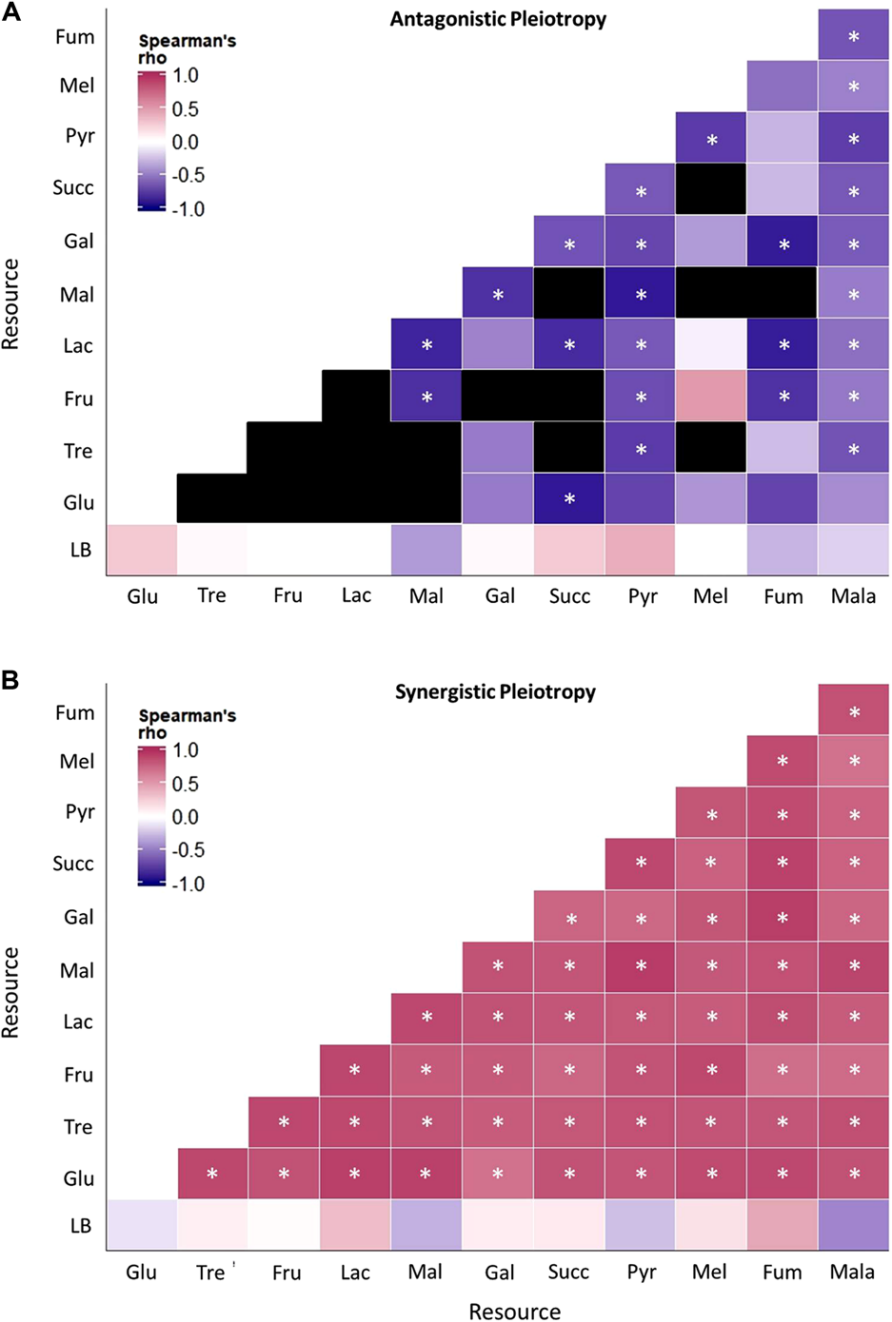
Correlation between primary and pleiotropic mutational effect size. Colored blocks indicate the coefficient of correlation between the magnitude of fitness effect sizes for a given pair of resources, for mutants that showed (A) AP or (B) SP. In panel A, black blocks represent cases where correlations were not computed because <5 mutants showed AP. Asterisks indicate a significant correlation (*P* < 0.05). Details for each correlation are given in Table S15 and Table S16.

We tested the relationship between primary and pleiotropic effect sizes for our set of random mutations, measuring primary effect sizes in LB, the growth medium in which our MA lines evolved. We measured secondary effect sizes in M9 minimal medium + 5 mM single carbon sources as above. Contrary to expectation, we found that for AP, in most cases the primary fitness effect sizes (in LB) were uncorrelated with the secondary effect sizes in specific carbon sources (bottom row, Fig. 4A; Table S15). Thus, the magnitude of fitness change in LB is unrelated to fitness change in other resources. For pairwise comparisons across single carbon sources, all significant correlations (25 of 39 possible comparisons; ∼64%) were negative (Fig. 4A). Thus, a large benefit in one carbon source was often associated with a small deleterious effect in another carbon source, or vice versa. Overall, antagonistic pleiotropic mutations either do not exhibit correlated fitness effects or show negatively correlated fitness effects in different environments. Synergistic pleiotropic effect sizes were also uncorrelated with primary effect sizes in LB (Fig. 4B; Table S16), suggesting that changes in fitness in a rich medium such as LB may generally not be related to fitness on individual carbon sources. However, all other pairwise resource combinations were strongly positive (Fig. 4B), indicating that large‐effect beneficial (or deleterious) mutations in one carbon source also had a large benefit (or disadvantage) in another carbon source. Thus, the predicted positive effect size correlations hold for synergistic, but not antagonistic pleiotropic effects.

## Discussion

In his artificial breeding experiments, Darwin observed Goethe's Law of Compensation in action, stating “if nourishment flows to one part or organ in excess, it rarely flows, at least in excess, to another part” (Darwin 1859). This concept of tradeoffs has played a central role in evolutionary thinking. Tradeoffs influence most major ecological and evolutionary processes (Agrawal et al. 2010), including speciation and adaptive radiation (Kneitel and Chase 2004), evolution of specialization (Bono et al. 2017; Elena 2017), evolution of life histories (Stearns 1977, 1989), and assembly and coexistence in ecological communities (Tilman 2000; Bohannan et al. 2002). In bacteria alone, tradeoffs affect many key physiological processes (reviewed in Ferenci 2016): nutrient utilization and metabolism, antibiotic resistance (see also Hershberg 2017), resistance to phages, resistance to environmental stress, virulence, and genome maintenance. However, the mechanisms underlying such phenotypic tradeoffs remain relatively poorly understood (Stearns 2000). A key mechanism is antagonistically pleiotropic mutations that can generate tradeoffs (Elena and Lenski 2003), but experimental measurements of the incidence, nature, and effect size of pleiotropic mutations are rare. Here, we present a systematic analysis of pleiotropic fitness effects of a large, unbiased sample of single mutations observed in *E. coli* populations evolving under weak selection.

Our results provide three clear lines of evidence suggesting that AP due to single mutations is unlikely to be an important mechanism generating carbon use tradeoffs that hinder adaptation. First, we find that AP is generally rare in new mutations. In fact, among beneficial mutations, AP is rarer than expected, indicating that beneficial mutations fixed during adaptation are unlikely to reduce fitness in other environments. Previous studies also found that only 10–14% of ∼20 beneficial mutations showed AP (Ostrowski et al. 2005; Dillon et al. 2016). Second, we find that large‐effect mutations are more likely to show AP in some (but not all) environments. Hence, AP may impose a major constraint only in specific environments and when adaptation involves large‐effect mutations. Finally, we find that antagonistically pleiotropic mutations often have negatively correlated fitness effects, such that a highly beneficial mutation in one environment is only weakly deleterious in an alternate environment, supporting previous studies that found similar results for beneficial mutations (Ostrowski et al. 2005; Jasmin and Zeyl 2013; Dillon et al. 2016). Thus, such mutations are unlikely to impose a significant fitness disadvantage in new habitats. Together, our results contradict the prevalent idea that tradeoffs generated by AP may often constrain adaptation.

Our analysis of 80 randomly sampled single mutational steps has several advantages over previous studies. First, we determined the expected distribution of the proportion of AP given the underlying distributions of fitness effects in different carbon sources, providing a general framework to determine the occurrence of AP by chance alone. This null distribution allowed us to determine that the observed proportion of AP is significantly lower than the expected proportion of AP for ∼71% of all resource pairs. Interestingly, we found that even the expected proportion of AP–derived from independently sampling from the DFEs of each resource in a pair–is very low (on average ∼16% across all resource pairs). This could be attributed to the fact that beneficial mutations are generally rare, and thus the probability of sampling a mutation that is beneficial in one resource and deleterious in another (i.e., showing AP) is very small. A second advantage of our experiment is that we measured fitness effects in 11 distinct carbon sources (55 resource pairs), a much larger set of environments than previous analyses. This allowed us to detect many more instances of pleiotropy: all but eight of our mutants showed AP for at least one pair of resources, and each mutant showed AP for a median of six resource pairs (out of 55). Finally, since our lines evolved under very weak selection, we were able to explore not only highly beneficial mutations, but the entire DFE for the occurrence of pleiotropy. This in turn allowed us to measure pleiotropic effects of a large set of mutations, making it possible to empirically test the relationship between fitness effect size and AP incidence.

We also note some important limitations of our work. First, to minimize false‐positive cases of pleiotropy due to error in measuring growth rates, we assumed that all mutations showing <5% change from the ancestor were neutral. Effectively, we may have thus ignored mutations with effect sizes <5%, potentially underestimating the incidence and effect sizes of antagonistically pleiotropic mutations. However, this seems unlikely because we found that for many resources, small‐effect mutations are depleted in AP. Second, we measured the incidence and nature of pleiotropy only for metabolic traits; specifically, for carbon utilization. Although we measured many more traits than previous studies, this is still a small fraction of traits that are probably relevant for ecological and evolutionary processes in bacteria. It is possible that antagonistic pleiotropy may be more frequent across diverse traits, such as those related to metabolism versus stress response. However, note that many studies focusing on adaptation in experimental microbial populations did not uncover tradeoffs in very diverse alternate environments (Björkman et al. 1998; Velicer and Lenski 1999; Reynolds 2000; Anderson et al. 2003; Lythgoe and Chao 2003; MacLean et al. 2004; Gagneux 2006; Kassen and Bataillon 2006; Buckling et al. 2007; Hughes et al. 2007; Ward et al. 2009; Bataillon et al. 2011; Vogwill et al. 2012; Jasmin and Zeyl 2013), suggesting that tradeoffs may be rare even across diverse traits. Finally, we caution that since our experiments were conducted for a relatively short time under controlled laboratory conditions, our results do not directly inform longer term phenomena such as ageing. Despite these limitations, our work represents the largest systematic analysis of single step mutational effects, and thus represents an important test of long‐held assumptions in evolutionary biology.

In summary, we provide new insights into the incidence, nature, and effect sizes of pleiotropic mutations affecting central carbon metabolism. Although phenotypic tradeoffs clearly influence many biological processes, we suggest that at the genetic level, tradeoffs may be generally rarer than expected. Antagonistic pleiotropy is thought to underlie the evolution and maintenance of generalists: AP may impose a cost of specialization on resource specialists, such that in heterogeneous environments, generalists that do not pay this cost are favoured (Cooper and Lenski 2000; Gompert and Messina 2016). Our results suggest that this broadly intuitive explanation needs to be more nuanced, because the incidence of AP varies significantly across environments. Thus, a generic “cost of specialization” cannot always explain the occurrence of generalists, but may have explanatory power in specific heterogeneous environments that include resource pairs showing high incidence of AP. Our work also demonstrates that whether evolution is largely driven by de novo (beneficial) mutations or by standing genetic variation (neutral or deleterious mutations), antagonistic pleiotropy is rare and cannot fully explain the pervasive fitness tradeoffs observed across environments. We hope that empirical quantification of the incidence and magnitude of AP across various organisms, environments, age classes, and genetic backgrounds will provide further insights into these issues. Ultimately, we need to integrate across mechanistic and phenotypic effects to better understand the role of tradeoffs in evolution.

Associate Editor: V. Cooper

Handling Editor: M. Servedio

## Supporting information


**Table S1**. Summary of the provenance of 80 focal mutants representing single mutational steps.
**Table S2**. Details of the 80 focal mutations representing single mutational steps.
**Table S3**. Output of generalized linear model with binomial errors for the effect of resource on the proportion of AP.
**Table S4**. Output of generalized linear model with binomial errors for the effect of resource on the proportion of SP.
**Table S5**. Tukey's post‐hoc pairwise comparisons between all resources in the generalized linear model (with binomial errors) for the effect of resource on proportion of AP.
**Table S6**. Tukey's post‐hoc pairwise comparisons between all resources in the generalized linear model (with binomial errors) for the effect of resource on proportion of SP.
**Table S7**. Output of generalized linear model with binomial errors for the effect of mutational step number on the proportion of AP.
**Table S8**. Output of chi‐squared test comparing the number of genes in molecular function categories in the GO database, with the observed molecular function categories for mutations which cause AP in less than 5 resource pairs or 5 to 10 resource pairs (also see Fig S9).
**Table S9**. Kendall's rank correlation between fitness effect bin and proportion of AP among all mutations.
**Table S10**. Kendall's rank correlation between fitness effect bin and proportion of AP among mutations that show AP.
**Table S11**. Kendall's rank correlation for the effect of fitness effect size on the proportion of AP in a null distribution of proportion of AP generated by randomly picking fitness values from the DFEs of each resource for each resource pair.
**Table S12**. Kendall's rank correlation between fitness effect bin and proportion of SP among all mutations.
**Table S13**. Kendall's rank correlation between fitness effect size and proportion of SP among mutations that show SP.
**Table S14**. Kendall's rank correlation for the effect of fitness effect size on the proportion of SP in a null distribution of proportion of SP generated by randomly picking fitness values from the DFEs of each resource for each resource pair.
**Table S15**. Spearman's two‐sided rank correlation for effect sizes of mutations showing AP across all resource pairs.
**Table S16**. Spearman's two‐sided rank correlation for effect sizes of mutations showing SP across all resource pairs.
**Fig S1**. Measured fitness of experimental isolates is consistent across two experimental runs.
**Fig S2**. Distribution of fitness effects of new mutations.
**Fig S3**. Fitness effects of single mutations in each resource.
**Fig S4**. Fitness effects of single mutations in all 55 resource pairs.
**Fig S5**. Fitness effects of non‐lethal single mutations in all 55 resource pairs.
**Fig S6**. Incidence of synergistic pleiotropy (SP) and antagonistic pleiotropy (AP) across resources.
**Fig S7**. Comparing the incidence of pleiotropy across first‐step and second‐step mutations.
**Fig S8**. Frequency of pleiotropy across resource pairs.
**Fig S9**. Functional classification of mutations showing AP.
**Fig S10**. Relationship between the incidence of SP and fitness effect size.
**Fig S11**. Observed proportion of AP is less than the null expectation in most resource pairs.
**Fig S12**. Observed proportion of SP is greater than the null expectation in most resource pairs.
**Fig S13**. Observed proportion of AP among beneficial mutations is less than the null expectation in most resource pairs.
**Fig S14**. Observed proportion of SP among beneficial mutations is less than the null expectation in most resource pairs.
**Fig S15**. Expected proportion of AP is negatively correlated with fitness effect size.
**Fig S16**. Expected proportion of SP is negatively correlated with fitness effect size.
