# Supplementary material for: Antagonistic pleiotropy for carbon use is rare in new mutations
Source: Evolution. 2018 Sep 13;72(10):2202–13. doi: 10.1111/evo.13569 (PMC6203952; doi:10.1111/evo.13569)
Supplement: Supplementary file 1 — Table S1. Summary of the provenance of 80 focal mutants representing single mutational steps. Table S2. Details of the 80 focal mutations representing single mutational steps. Table S3. Output of generalized linear model with binomial errors for the effect of resource on the proportion of AP. Table S4. Output of generalized linear model with binomial errors for the effect of resource on the proportion of SP. Table S5. Tukey's post‐hoc pairwise comparisons between all resources in the generalized linear model (with binomial errors) for the effect of resource on proportion of AP. Table S6. Tukey's post‐hoc pairwise comparisons between all resources in the generalized linear model (with binomial errors) for the effect of resource on proportion of SP. Table S7. Output of generalized linear model with binomial errors for the effect of mutational step number on the proportion of AP. Table S8. Output of chi‐squared test comparing the number of genes in molecular function categories in the GO database, with the observed molecular function categories for mutations which cause AP in less than 5 resource pairs or 5 to 10 resource pairs (also see Fig S9). Table S9. Kendall's rank correlation between fitness effect bin and proportion of AP among all mutations. Table S10. Kendall's rank correlation between fitness effect bin and proportion of AP among mutations that show AP. Table S11. Kendall's rank correlation for the effect of fitness effect size on the proportion of AP in a null distribution of proportion of AP generated by randomly picking fitness values from the DFEs of each resource for each resource pair. Table S12. Kendall's rank correlation between fitness effect bin and proportion of SP among all mutations. Table S13. Kendall's rank correlation between fitness effect size and proportion of SP among mutations that show SP. Table S14. Kendall's rank correlation for the effect of fitness effect size on the proportion of SP in a null distribution of proportion of SP genera [file EVO-72-2202-s001.docx]

**SUPPLEMENTARY INFORMATION**

**SUPPLEMENTARY METHODS**

**Whole genome sequencing of MA lines**

We inoculated 2µL of each frozen stock (and its ancestor) in 2mL LB and allowed cells to grow overnight at 37ºC with shaking at 200rpm. We extracted genomic DNA (GenElute Bacterial Genomic DNA kit, Sigma-Aldrich), quantified DNA (Qubit HS dsDNA assay, Invitrogen), and prepared genomic DNA libraries (Nextera XT DNA Library Preparation Kit, Illumina), following manufacturer’s instructions in each case. We sequenced libraries on the Illumina Hi-seq 2500 platform using either 2x100bp paired-end reaction chemistry (~150x coverage per genome; range 102x to 240x) or 1x100 single-end reaction chemistry (~70x coverage per genome; range 21x to 140x). We discarded reads with quality scores less than Q30, retaining >95% of reads per genome. We aligned filtered reads to the NCBI reference *E. coli* K-12 MG1655 genome (RefSeq accession ID GCA_000005845.2) using the Burrows-Wheeler short-read alignment tool, BWA (Li and Durbin 2009). We generated pileup files using SAMtools (Li et al. 2009), and used the VARSCAN package to extract a list of SNPs (single nucleotide changes) and short indels (<10bp) (Koboldt et al. 2009). To identify long indels (>10bp), we used Breseq (Deatherage and Barrick 2014). We discarded all mutations that occurred at <80% frequency or were supported by <10 reads on both strands, and one SNP and one indel that were shared across all evolved MA lines and the WT ancestor. We did not find any long indels in our sequenced isolates. We used the Ecocyc database (Keseler et al. 2013) to classify all mutations (except intergenic mutations) according to the Gene Ontology molecular function categories of the genes in which they occurred (S2 Table).

**Measuring growth rate as a fitness measure**

We measured growth rates of all mutants and their respective ancestors (Fig. 1B) in liquid culture media: LB broth (Miller, Difco), or M9 minimal salts medium + 5mM of a carbon source (glucose, trehalose, fructose, maltose, lactose, galactose, succinate, pyruvate, melibiose, malate, fumarate; Sigma-Aldrich). We inoculated each mutant from its freezer stock into M9 minimal salts medium with 0.4% glucose, kept at 37ºC with shaking at 200 rpm for 16 hours. We inoculated 6µL of this culture into 594µL growth media (LB broth or M9 minimal salts medium + 5mM carbon source) in 48-well plates (Costar) incubated in a shaking tower (Liconic) at 37ºC. Plates were read by an automated growth measurement system (Tecan, Austria) every 40 minutes for 18 hours. We measured the growth rate of three technical replicates per mutant per carbon source; meaningful biological replicates could not be obtained since we had a single colony at the end of each MA line transfer. In each 48-well plate, we included the WT ancestor to estimate variation in growth rates across plates and to calculate relative fitness of evolved isolates. We estimated maximum growth rates using the Curve Fitter software (Delaney et al. 2013).

**Binning fitness effects for analysing effect of mutational fitness effect sizes on proportion of AP**

We wanted to test the hypothesis that large-effect mutations are more likely to show AP (see Fig 3). This statement is agnostic to the direction of the fitness effect. Hence, for each focal resource in turn, we first classified mutations based on the absolute value of fitness effect in the focal resource (retaining information on the sign of the effect for later use). Next, we had to determine whether the incidence of AP varies as a function of focal effect size. We cannot calculate % AP unless we bin mutations by their effect size in the focal resource. Thus, we created four arbitrary bins for the magnitude of the mutational effect size. Then we asked how many of the mutations showed AP (here, the sign of the effect was considered: by definition, mutations showing AP will have a positive fitness effect in resource 1 and a negative effect in resource 2). In the results shown in Fig 4, we consider both the magnitude and the sign of the fitness effect sizes: for each pair of resources, we separated mutations that showed either SP or AP (using the sign of the fitness effect), and then asked whether the magnitude of the fitness change in the two resources was correlated.

**REFERENCES**

Deatherage, D. E., and J. E. Barrick. 2014. Identification of mutations in laboratory evolved microbes from next-generation sequencing data using breseq. Methods Mol. Biol. 1151:165–188.

Delaney, N. F., J. I. R. Echenique, and C. J. Marx. 2013. Clarity: An open-source manager for laboratory automation. J. Lab. Autom. 18:171–177.

Keseler, I. M., A. Mackie, M. Peralta-Gil, A. Santos-Zavaleta, S. Gama-Castro, C. Bonavides-Martínez, C. Fulcher, A. M. Huerta, A. Kothari, M. Krummenacker, M. Latendresse, L. Muñiz-Rascado, Q. Ong, S. Paley, I. Schröder, A. G. Shearer, P. Subhraveti, M. Travers, D. Weerasinghe, V. Weiss, J. Collado-Vides, R. P. Gunsalus, I. Paulsen, and P. D. Karp. 2013. EcoCyc: Fusing model organism databases with systems biology. Nucleic Acids Res. 41:605–612.

Koboldt, D. C., K. Chen, T. Wylie, D. E. Larson, M. D. McLellan, E. R. Mardis, G. M. Weinstock, R. K. Wilson, and L. Ding. 2009. VarScan: Variant detection in massively parallel sequencing of individual and pooled samples. Bioinformatics 25:2283–2285.

Li, H., and R. Durbin. 2009. Fast and accurate short read alignment with Burrows-Wheeler transform. Bioinformatics 25:1754–1760.

Li, H., B. Handsaker, A. Wysoker, T. Fennell, J. Ruan, N. Homer, G. Marth, G. Abecasis, and R. Durbin. 2009. The Sequence Alignment/Map format and SAMtools. Bioinformatics 25:2078–2079.

**SUPPLEMENTARY TABLES**

**S1 Table. Summary of the provenance of 80 focal mutants representing single mutational steps.** We initiated the MA experiment with 40 independent lines, of which we omitted two (lines 34 and 35) because of a mistake in the transfer protocol. Grey cells indicate single mutational steps, i.e. time points used to test fitness effects. Each single mutational step is marked by a unique number (m1 to m80). All other cells indicate isolates that were not useful for our analysis: white cells indicate multiple mutational steps relative to the previous time point , and “X” indicates no new mutations. “ND” indicates cases where sequence data were not obtained and mutations could not be identified.

| Line | Day 39 | Day 104 | Day 140 | Day 200 | Day 250 | Day 300 | Total mutations  at Day 300 |
| --- | --- | --- | --- | --- | --- | --- | --- |
| 1 | m1 |  | m26 |  | m52 |  | 8 |
| 2 |  |  |  |  |  |  | 10 |
| 3 | X |  |  |  | X | X | 5 |
| 4 | m2 |  | X | m34 |  | ND | ND |
| 5 | m3 | m13 | X | m35 |  | m64 | 5 |
| 6 | m4 | m14 | ND | m36 | X | m65 | 4 |
| 7 | X | m15 | m27 | m37 | ND | m66 | 5 |
| 8 | X | m16 | X | m38 | X | X | 2 |
| 9 |  | m17 | X | X | m53 | X | 4 |
| 10 |  | m18 |  | m39 |  |  | 4 |
| 11 | X | m19 | X | m40 |  | X | 5 |
| 12 | m5 |  |  |  |  | m67 | 6 |
| 13 | m6 | m20 | m28 |  |  |  | 7 |
| 14 |  |  |  | m41 |  | X | 7 |
| 15 | X |  | X | X |  |  | 8 |
| 16 |  |  |  |  |  |  | 16 |
| 17 | X | m21 | X | m42 | ND | m68 | 3 |
| 18 | X | X | X | X | m54 | m69 | 2 |
| 19 | X |  | X | m43 |  | X | 11 |
| 20 | ND |  | ND |  | m55 | m70 | 8 |
| 21 | X |  | X | m44 | m56 | m71 | 5 |
| 22 |  |  | m29 |  |  | m72 | 10 |
| 23 | m7 | X | X |  | m57 | X | 6 |
| 24 | m8 |  | m30 | m45 |  | m73 | 7 |
| 25 |  |  | X |  | m58 | m74 | 9 |
| 26 |  | m22 |  |  | m59 | X | 9 |
| 27 | X | m23 | m31 | m46 |  | X | 5 |
| 28 | m9 | m24 | m32 |  | X | X | 6 |
| 29 | m10 |  |  | m47 | X | m75 | 8 |
| 30 |  |  | X | m48 | X | m76 | 7 |
| 31 |  |  |  | X | m60 | m77 | 8 |
| 32 | ND |  | ND | m49 | m61 | m78 | 5 |
| 33 |  |  |  |  | ND |  | 10 |
| 36 | m11 |  |  | m50 |  |  | 13 |
| 37 |  |  |  |  | m62 | X | 9 |
| 38 |  |  | m33 | X | X | m79 | 7 |
| 39 | m12 | m25 | X |  | m63 | m80 | 6 |
| 40 |  |  |  | m51 |  | X | 6 |

**S2 Table. Details of the 80 focal mutations representing single mutational steps.** The provenance of each mutational step (first column) is given in S1 Table. The last column lists the Gene Ontology (GO) term associated with each mutation. Cases where information about a gene was not available are marked “unknown”. Non-coding mutations could not be classified. IGR = intergenic region.

| Mutation | Annotation | Type | Coding? | Synonymous? | GO terms (Molecular Function) |
| --- | --- | --- | --- | --- | --- |
| m1 | mntR | indel | Y | NA | transcription regulator |
| m2 | IGR | indel | N | NA | NA |
| m3 | IGR | snp | N | NA | NA |
| m4 | yehD | snp | Y | nonsyn | transporter activity |
| m5 | sslE | snp | Y | nonsyn | unknown |
| m6 | cutC | snp | Y | nonsyn | binding activity |
| m7 | IGR | snp | N | NA | NA |
| m8 | rpsA | snp | Y | syn | structural molecule |
| m9 | rplD | snp | Y | nonsyn | transcription regulator |
| m10 | torS | snp | Y | syn | catalytic activity |
| m11 | paaH | snp | Y | syn | catalytic activity |
| m12 | ydcK | snp | Y | syn | catalytic activity |
| m13 | cysG | snp | Y | nonsyn | catalytic activity |
| m14 | glcG | snp | Y | nonsyn | catalytic activity |
| m15 | efp | snp | Y | nonsyn | binding activity |
| m16 | xapB | snp | Y | nonsyn | catalytic activity |
| m17 | rapA | snp | Y | nonsyn | binding activity |
| m18 | IGR | snp | N | NA | NA |
| m19 | yhjC | snp | Y | nonsyn | binding activity |
| m20 | narQ | snp | Y | nonsyn | catalytic activity |
| m21 | ynbB | snp | Y | nonsyn | catalytic activity |
| m22 | pta | snp | Y | nonsyn | catalytic activity |
| m23 | yjgL | snp | Y | nonsyn | binding activity |
| m24 | mocA | snp | Y | syn | binding activity |
| m25 | IGR | snp | N | NA | NA |
| m26 | tehB | snp | Y | nonsyn | catalytic activity |
| m27 | yhdP | snp | Y | nonsyn | unknown |
| m28 | yfaQ | snp | Y | nonsyn | unknown |
| m29 | yffS | snp | Y | nonsyn | binding activity |
| m30 | IGR | snp | N | NA | NA |
| m31 | IGR | snp | N | NA | NA |
| m32 | IGR | indel | N | NA | NA |
| m33 | citT | snp | Y | nonsyn | transporter activity |
| m34 | fecE | snp | Y | nonsyn | binding activity |
| m35 | IGR | indel | N | NA | NA |
| m36 | helD | snp | Y | nonsyn | catalytic activity |
| m37 | glpA | snp | Y | nonsyn | catalytic activity |
| m38 | ilvD | snp | Y | nonsyn | catalytic activity |
| m39 | rrlC | snp | Y | syn | binding activity |
| m40 | IGR | snp | N | NA | NA |
| m41 | wecF | snp | Y | NA | catalytic activity |
| m42 | btuB | snp | Y | nonsyn | binding activity |
| m43 | metC | snp | Y | nonsyn | catalytic activity |
| m44 | hyfF | snp | Y | syn | catalytic activity |
| m45 | ycdU | snp | Y | nonsyn | unknown |
| m46 | csgA | snp | Y | nonsyn | binding activity |
| m47 | yddB | snp | Y | nonsyn | binding activity |
| m48 | rlmB | snp | Y | nonsyn | catalytic activity |
| m49 | purM | snp | Y | syn | catalytic activity |
| m50 | IGR | snp | N | NA | NA |
| m51 | yhfX | snp | Y | nonsyn | binding activity |
| m52 | ppx | snp | Y | nonsyn | catalytic activity |
| m53 | putA | snp | Y | nonsyn | transcription regulator |
| m54 | nlpI | snp | Y | nonsyn | binding activity |
| m55 | mraZ | snp | Y | syn | binding activity |
| m56 | ygjR | snp | Y | syn | binding activity |
| m57 | bioD | snp | Y | nonsyn | catalytic activity |
| m58 | fadH | snp | Y | nonsyn | catalytic activity |
| m59 | IGR | snp | N | NA | NA |
| m60 | insJ | snp | Y | syn | binding activity |
| m61 | ybbN | snp | Y | nonsyn | binding activity |
| m62 | IGR | snp | N | NA | NA |
| m63 | puuB | snp | Y | nonsyn | catalytic activity |
| m64 | IGR | indel | N | NA | NA |
| m65 | leuW | indel | Y | NA | binding activity |
| m66 | oppC | snp | Y | nonsyn | binding activity |
| m67 | IGR | snp | N | NA | NA |
| m68 | panB | snp | Y | syn | catalytic activity |
| m69 | IGR | snp | N | NA | NA |
| m70 | prpE | snp | Y | syn | catalytic activity |
| m71 | yciF | snp | Y | nonsyn | binding activity |
| m72 | yhhH | snp | Y | nonsyn | unknown |
| m73 | IGR | snp | N | NA | NA |
| m74 | IGR | snp | N | NA | NA |
| m75 | rplU | snp | Y | nonsyn | structural molecule |
| m76 | dld | snp | Y | nonsyn | catalytic activity |
| m77 | clpA | snp | Y | nonsyn | binding activity |
| m78 | rhlB | indel | Y | NA | binding activity |
| m79 | fryA | snp | Y | nonsyn | catalytic activity |
| m80 | IGR | snp | N | NA | NA |

**S3 Table.** Output of generalized linear model with binomial errors for the effect of resource on the proportion of AP. Model: glm(I(cbind(AP, non_AP))~as.factor(focal_resource) , family=binomial).

| **Treatment** | **Model coefficient** | **95% confidence interval** | | **Odds ratio (e^coefficient^)** | **P** |
| --- | --- | --- | --- | --- | --- |
|  |  | **Lower** | **Upper** |  |  |
| Intercept (Glucose) | -2.549 | -2.827 | -2.291 |  | 5.44E-78 |
| Resource - Trehalose | -0.097 | -0.485 | 0.289 | 0.908 | 0.623 |
| Resource - Fructose | 0.156 | -0.209 | 0.525 | 1.169 | 0.402 |
| Resource - Lactose | 0.379 | 0.030 | 0.734 | 1.461 | 0.035 |
| Resource - Maltose | 0.072 | -0.300 | 0.446 | 1.075 | 0.704 |
| Resource - Galactose | 0.814 | 0.489 | 1.150 | 2.258 | 1.35E-06 |
| Resource - Succinate | 0.054 | -0.319 | 0.429 | 1.056 | 0.775 |
| Resource - Pyruvate | 0.834 | 0.509 | 1.169 | 2.302 | 7.06E-07 |
| Resource - Melibiose | 0.235 | -0.124 | 0.599 | 1.265 | 0.201 |
| Resource - Fumarate | 0.338 | -0.014 | 0.695 | 1.402 | 0.062 |
| Resource - Malate | 1.355 | 1.047 | 1.676 | 3.875 | 2.51E-17 |

**S4 Table.** Output of generalized linear model with binomial errors for the effect of resource on the proportion of SP. Model: glm(I(cbind(SP, non_SP))~as.factor(focal_resource) , family=binomial).

| **Treatment** | **Model coefficient** | **95% confidence interval** | | **Odds ratio**  **(e^coefficient^)** | **P** |
| --- | --- | --- | --- | --- | --- |
|  |  | **lower** | **upper** |  |  |
| Intercept - Glucose | -1.208 | -1.376 | -1.046 |  | 6.67E-47 |
| Resource - Trehalose | 0.839 | 0.623 | 1.057 | 10.119 | 3.25E-14 |
| Resource - Fructose | 1.038 | 0.824 | 1.255 | 16.832 | 3.81E-21 |
| Resource - Lactose | 0.534 | 0.314 | 0.755 | 5.504 | 2.07E-06 |
| Resource - Maltose | 0.195 | -0.033 | 0.422 | 3.370 | 0.093 |
| Resource - Galactose | 0.829 | 0.613 | 1.047 | 9.881 | 6.81E-14 |
| Resource - Succinate | 0.932 | 0.717 | 1.149 | 12.660 | 2.92E-17 |
| Resource - Pyruvate | 0.681 | 0.464 | 0.901 | 7.219 | 9.57E-10 |
| Resource - Melibiose | 1.093 | 0.879 | 1.310 | 19.762 | 2.56E-23 |
| Resource - Fumarate | -0.334 | -0.580 | -0.089 | 2.047 | 0.008 |
| Resource - Malate | 0.282 | 0.058 | 0.508 | 3.767 | 0.014 |

**S5 Table.** Tukey’s post-hoc pairwise comparisons between all resources in the generalized linear model (with binomial errors) for the effect of resource on proportion of AP. Model: glht(AP_prop_glm, linfct = mcp(res1 = "Tukey")).

| **Comparison** | **Estimate** | **Std. Error** | **Statistic** | **P** |
| --- | --- | --- | --- | --- |
| Trehalose - Glucose | -0.097 | 0.197 | -0.492 | 0.999993 |
| Fructose - Glucose | 0.156 | 0.187 | 0.838 | 0.999018 |
| Lactose - Glucose | 0.379 | 0.179 | 2.114 | 0.560981 |
| Maltose - Glucose | 0.072 | 0.190 | 0.380 | 0.999999 |
| Galactose - Glucose | 0.814 | 0.169 | 4.833 | 5.78E-05 |
| Succinate - Glucose | 0.054 | 0.191 | 0.286 | 1 |
| Pyruvate - Glucose | 0.834 | 0.168 | 4.960 | 3.54E-05 |
| Melibiose - Glucose | 0.235 | 0.184 | 1.279 | 0.971415 |
| Fumarate - Glucose | 0.338 | 0.181 | 1.869 | 0.731482 |
| Malate - Glucose | 1.355 | 0.160 | 8.468 | 2.22E-16 |
| Fructose - Trehalose | 0.253 | 0.191 | 1.326 | 0.963245 |
| Lactose - Trehalose | 0.476 | 0.184 | 2.589 | 0.24891 |
| Maltose - Trehalose | 0.169 | 0.194 | 0.870 | 0.998633 |
| Galactose - Trehalose | 0.911 | 0.173 | 5.260 | 4.98E-06 |
| Succinate - Trehalose | 0.151 | 0.195 | 0.777 | 0.999493 |
| Pyruvate - Trehalose | 0.931 | 0.173 | 5.384 | 1.64E-06 |
| Melibiose - Trehalose | 0.332 | 0.188 | 1.764 | 0.797011 |
| Fumarate - Trehalose | 0.435 | 0.185 | 2.348 | 0.394278 |
| Malate - Trehalose | 1.452 | 0.165 | 8.799 | 1.11E-16 |
| Lactose - Fructose | 0.223 | 0.173 | 1.288 | 0.969838 |
| Maltose - Fructose | -0.084 | 0.184 | -0.459 | 0.999996 |
| Galactose - Fructose | 0.658 | 0.162 | 4.073 | 0.002097 |
| Succinate - Fructose | -0.102 | 0.184 | -0.553 | 0.999977 |
| Pyruvate - Fructose | 0.677 | 0.161 | 4.204 | 0.001266 |
| Melibiose - Fructose | 0.079 | 0.178 | 0.444 | 0.999997 |
| Fumarate - Fructose | 0.181 | 0.174 | 1.041 | 0.993984 |
| Malate - Fructose | 1.198 | 0.153 | 7.851 | 3.91E-14 |
| Maltose - Lactose | -0.307 | 0.176 | -1.742 | 0.809187 |
| Galactose - Lactose | 0.435 | 0.153 | 2.845 | 0.138407 |
| Succinate - Lactose | -0.325 | 0.177 | -1.834 | 0.754385 |
| Pyruvate - Lactose | 0.455 | 0.153 | 2.981 | 0.096508 |
| Melibiose - Lactose | -0.144 | 0.170 | -0.847 | 0.99892 |
| Fumarate - Lactose | -0.041 | 0.166 | -0.249 | 1 |
| Malate - Lactose | 0.976 | 0.144 | 6.798 | 1.14E-10 |
| Galactose - Maltose | 0.742 | 0.165 | 4.493 | 0.000317 |
| Succinate - Maltose | -0.018 | 0.188 | -0.094 | 1 |
| Pyruvate - Maltose | 0.762 | 0.165 | 4.622 | 0.000183 |
| Melibiose - Maltose | 0.163 | 0.181 | 0.902 | 0.998157 |
| Fumarate - Maltose | 0.266 | 0.178 | 1.496 | 0.91913 |
| Malate - Maltose | 1.283 | 0.156 | 8.196 | 5.00E-15 |
| Succinate - Galactose | -0.760 | 0.166 | -4.578 | 0.000176 |
| Pyruvate - Galactose | 0.019 | 0.140 | 0.140 | 1 |
| Melibiose - Galactose | -0.579 | 0.158 | -3.657 | 0.011578 |
| Fumarate - Galactose | -0.477 | 0.154 | -3.086 | 0.071911 |
| Malate - Galactose | 0.540 | 0.130 | 4.168 | 0.001447 |
| Pyruvate - Succinate | 0.779 | 0.166 | 4.706 | 9.32E-05 |
| Melibiose - Succinate | 0.181 | 0.182 | 0.995 | 0.995807 |
| Fumarate - Succinate | 0.283 | 0.178 | 1.588 | 0.884346 |
| Malate - Succinate | 1.300 | 0.157 | 8.264 | 1.55E-15 |
| Melibiose - Pyruvate | -0.599 | 0.158 | -3.790 | 0.007252 |
| Fumarate - Pyruvate | -0.496 | 0.154 | -3.221 | 0.048259 |
| Malate - Pyruvate | 0.521 | 0.129 | 4.033 | 0.002679 |
| Fumarate - Melibiose | 0.102 | 0.171 | 0.598 | 0.999953 |
| Malate - Melibiose | 1.119 | 0.149 | 7.501 | 5.36E-13 |
| Malate - Fumarate | 1.017 | 0.145 | 7.009 | 1.24E-10 |

**S6 Table.** Tukey’s post-hoc pairwise comparisons between all resources in the generalized linear model (with binomial errors) for the effect of resource on proportion of SP. Model: glht(SP_prop_glm, linfct = mcp(res1 = "Tukey")).

| **Comparison** | **Estimate** | **Std. Error** | **Statistic** | **P** |
| --- | --- | --- | --- | --- |
| Trehalose - Glucose | 0.839 | 0.111 | 7.588 | 7.08E-13 |
| Fructose - Glucose | 1.038 | 0.110 | 9.438 | 0 |
| Lactose - Glucose | 0.534 | 0.112 | 4.747 | 0.000212 |
| Maltose - Glucose | 0.195 | 0.116 | 1.677 | 0.846552 |
| Galactose - Glucose | 0.829 | 0.111 | 7.491 | 8.75E-13 |
| Succinate - Glucose | 0.932 | 0.110 | 8.450 | 5.55E-16 |
| Pyruvate - Glucose | 0.681 | 0.111 | 6.116 | 4.75E-08 |
| Melibiose - Glucose | 1.093 | 0.110 | 9.948 | 0 |
| Fumarate - Glucose | -0.334 | 0.125 | -2.666 | 0.213893 |
| Malate - Glucose | 0.282 | 0.115 | 2.457 | 0.328425 |
| Fructose - Trehalose | 0.199 | 0.101 | 1.967 | 0.670468 |
| Lactose - Trehalose | -0.305 | 0.104 | -2.943 | 0.109881 |
| Maltose - Trehalose | -0.645 | 0.108 | -5.993 | 3.73E-08 |
| Galactose - Trehalose | -0.010 | 0.102 | -0.102 | 1 |
| Succinate - Trehalose | 0.092 | 0.101 | 0.912 | 0.998037 |
| Pyruvate - Trehalose | -0.158 | 0.103 | -1.537 | 0.906622 |
| Melibiose - Trehalose | 0.254 | 0.101 | 2.516 | 0.294126 |
| Fumarate - Trehalose | -1.173 | 0.117 | -9.990 | 0 |
| Malate - Trehalose | -0.557 | 0.106 | -5.233 | 6.92E-06 |
| Lactose - Fructose | -0.504 | 0.103 | -4.890 | 4.94E-05 |
| Maltose - Fructose | -0.843 | 0.107 | -7.886 | 2.78E-14 |
| Galactose - Fructose | -0.209 | 0.101 | -2.068 | 0.597714 |
| Succinate - Fructose | -0.106 | 0.101 | -1.056 | 0.993409 |
| Pyruvate - Fructose | -0.356 | 0.102 | -3.497 | 0.020148 |
| Melibiose - Fructose | 0.055 | 0.100 | 0.551 | 0.999979 |
| Fumarate - Fructose | -1.372 | 0.117 | -11.741 | 0 |
| Malate - Fructose | -0.756 | 0.106 | -7.143 | 2.73E-11 |
| Maltose - Lactose | -0.339 | 0.109 | -3.099 | 0.07036 |
| Galactose - Lactose | 0.295 | 0.104 | 2.842 | 0.142129 |
| Succinate - Lactose | 0.398 | 0.103 | 3.847 | 0.005534 |
| Pyruvate - Lactose | 0.148 | 0.105 | 1.411 | 0.945625 |
| Melibiose - Lactose | 0.559 | 0.103 | 5.431 | 2.97E-06 |
| Fumarate - Lactose | -0.868 | 0.119 | -7.280 | 2.73E-12 |
| Malate - Lactose | -0.251 | 0.108 | -2.321 | 0.418063 |
| Galactose - Maltose | 0.634 | 0.108 | 5.894 | 1.20E-07 |
| Succinate - Maltose | 0.737 | 0.107 | 6.874 | 3.39E-10 |
| Pyruvate - Maltose | 0.487 | 0.108 | 4.491 | 0.000376 |
| Melibiose - Maltose | 0.899 | 0.107 | 8.411 | 1.33E-15 |
| Fumarate - Maltose | -0.528 | 0.123 | -4.312 | 0.00088 |
| Malate - Maltose | 0.088 | 0.112 | 0.784 | 0.999471 |
| Succinate - Galactose | 0.103 | 0.101 | 1.013 | 0.995309 |
| Pyruvate - Galactose | -0.147 | 0.103 | -1.436 | 0.939196 |
| Melibiose - Galactose | 0.264 | 0.101 | 2.618 | 0.238126 |
| Fumarate - Galactose | -1.162 | 0.117 | -9.899 | 0 |
| Malate - Galactose | -0.546 | 0.106 | -5.133 | 1.01E-05 |
| Pyruvate - Succinate | -0.250 | 0.102 | -2.446 | 0.335507 |
| Melibiose - Succinate | 0.162 | 0.101 | 1.607 | 0.879051 |
| Fumarate - Succinate | -1.265 | 0.117 | -10.807 | 0 |
| Malate - Succinate | -0.649 | 0.106 | -6.121 | 1.95E-08 |
| Melibiose - Pyruvate | 0.412 | 0.102 | 4.043 | 0.002536 |
| Fumarate - Pyruvate | -1.015 | 0.118 | -8.590 | 0 |
| Malate - Pyruvate | -0.399 | 0.107 | -3.721 | 0.009408 |
| Fumarate - Melibiose | -1.427 | 0.117 | -12.223 | 0 |
| Malate - Melibiose | -0.811 | 0.106 | -7.673 | 1.46E-13 |
| Malate - Fumarate | 0.616 | 0.121 | 5.071 | 2.33E-05 |

**S7 Table.** Output of generalized linear model with binomial errors for the effect of mutational step number on the proportion of AP. Model: glm(I(cbind(AP, non_AP))~as.factor(mut_step), family=binomial).

| **Resource** | **Treatment** | **Model coefficient** | **95% confidence interval** | | **Odds ratio** | **P** |
| --- | --- | --- | --- | --- | --- | --- |
|  |  |  | **Lower** | **Upper** |  |  |
| Glucose | Intercept - first mutational step | -2.450 | -2.867 | -2.077 |  | 2.54E-34 |
|  | second mutational step | -0.228 | -0.861 | 0.384 | 0.796 | 0.471 |
| Trehalose | Intercept - first mutational step | -2.944 | -3.471 | -2.490 |  | 2.64E-32 |
|  | second mutational step | 0.324 | -0.352 | 1.008 | 1.383 | 0.346 |
| Fructose | Intercept - first mutational step | -2.671 | -3.132 | -2.264 |  | 8.69E-34 |
|  | second mutational step | 0.513 | -0.062 | 1.101 | 1.670 | 0.082 |
| Lactose | Intercept - first mutational step | -1.855 | -2.178 | -1.555 |  | 1.29E-31 |
|  | second mutational step | -0.303 | -0.807 | 0.186 | 0.738 | 0.229 |
| Maltose | Intercept - first mutational step | -2.578 | -3.019 | -2.186 |  | 4.27E-34 |
|  | second mutational step | 0.164 | -0.436 | 0.762 | 1.178 | 0.589 |
| Galactose | Intercept - first mutational step | -1.987 | -2.328 | -1.673 |  | 8.20E-33 |
|  | second mutational step | 0.307 | -0.151 | 0.768 | 1.360 | 0.189 |
| Succinate | Intercept - first mutational step | -2.411 | -2.820 | -2.044 |  | 2.43E-34 |
|  | second mutational step | -0.209 | -0.828 | 0.390 | 0.811 | 0.498 |
| Pyruvate | Intercept - first mutational step | -1.987 | -2.328 | -1.673 |  | 8.21E-33 |
|  | second mutational step | 0.360 | -0.094 | 0.817 | 1.434 | 0.120 |
| Melibiose | Intercept - first mutational step | -2.578 | -3.019 | -2.186 |  | 4.27E-34 |
|  | second mutational step | 0.530 | -0.021 | 1.094 | 1.699 | 0.061 |
| Fumarate | Intercept - first mutational step | -1.782 | -2.096 | -1.489 |  | 8.59E-31 |
|  | second mutational step | -0.839 | -1.416 | -0.299 | 0.432 | 0.003 |
| Malate | Intercept - first mutational step | -1.735 | -2.043 | -1.447 |  | 3.29E-30 |
|  | second mutational step | 1.178 | 0.798 | 1.568 | 3.247 | 1.93E-09 |

**S8 Table.** Output of chi-squared test comparing the number of genes in molecular function categories in the GO database, with the observed molecular function categories for mutations which cause AP in less than 5 resource pairs or 5 to 10 resource pairs (also see Fig S9).

| **Treatment** | **statistic** | **P** | **parameter** |
| --- | --- | --- | --- |
| GO database vs. Observed (AP in < 5 resource pairs) | 48 | 0.313 | 44 |
| GO database vs. Observed (AP in 5-10 resource pairs) | 36 | 0.329 | 33 |

**S9 Table.** Kendall’s rank correlation between fitness effect bin and proportion of AP among all mutations. Model: cor.test(resource$fitness_effect_bin, resource$AP_prop, method="kendall").

| **Kendall's Rank correlation** | **Kendall's tau** | **P** |
| --- | --- | --- |
| Proportion of AP vs. fitness effect in Glucose | 0.390 | 0.0005 |
| Proportion of AP vs. fitness effect in Trehalose | -0.007 | 0.951 |
| Proportion of AP vs. fitness effect in Fructose | 0.502 | 6.43E-07 |
| Proportion of AP vs. fitness effect in Lactose | -0.304 | 0.001 |
| Proportion of AP vs. fitness effect in Maltose | 0.207 | 0.050 |
| Proportion of AP vs. fitness effect in Galactose | 0.264 | 0.0004 |
| Proportion of AP vs. fitness effect in Succinate | -0.094 | 0.415 |
| Proportion of AP vs. fitness effect in Pyruvate | -0.593 | 7.68E-15 |
| Proportion of AP vs. fitness effect in Melibiose | 0.205 | 0.049 |
| Proportion of AP vs. fitness effect in Fumarate | 0.222 | 0.028 |
| Proportion of AP vs. fitness effect in Malate | -0.091 | 0.134 |

**S10 Table.** Kendall’s rank correlation between fitness effect bin and proportion of AP among mutations that show AP. Model: cor.test(resource$fitness_effect_bin, resource$AP_prop, method="kendall").

| **Kendall's Rank correlation** | **Kendall's tau** | **P** |
| --- | --- | --- |
| Proportion of AP vs. Fitness effect in glucose | 0.302 | 0.003 |
| Proportion of AP vs. Fitness effect in trehalose | 0.135 | 0.193 |
| Proportion of AP vs. Fitness effect in fructose | 0.189 | 0.037 |
| Proportion of AP vs. Fitness effect in lactose | 0.254 | 0.002 |
| Proportion of AP vs. Fitness effect in maltose | 0.131 | 0.183 |
| Proportion of AP vs. Fitness effect in galactose | 0.041 | 0.533 |
| Proportion of AP vs. Fitness effect in succinate | 0.092 | 0.338 |
| Proportion of AP vs. Fitness effect in pyruvate | 0.296 | 7.53E-06 |
| Proportion of AP vs. Fitness effect in melibiose | 0.275 | 0.001 |
| Proportion of AP vs. Fitness effect in fumarate | 0.376 | 7.77E-06 |
| Proportion of AP vs. Fitness effect in malate | 0.145 | 0.006 |

**S11 Table.** Kendall’s rank correlation for the effect of fitness effect size on the proportion of AP in a null distribution of proportion of AP generated by randomly picking fitness values from the DFEs of each resource for each resource pair. Model_correlation: cor.test(resource$fitness_effect_bin, resource$null_AP_prop, method="kendall").

| **Resource pair** | **Estimate** | **Statistic** | **P** |
| --- | --- | --- | --- |
| Trehalose - Glucose | -0.68802 | -17.338 | 2.43E-67 |
| Fructose - Glucose | -0.73047 | -18.5371 | 1.04E-76 |
| Lactose - Glucose | -0.6619 | -16.6459 | 3.24E-62 |
| Maltose - Glucose | -0.686 | -17.0578 | 3.06E-65 |
| Galactose - Glucose | -0.75274 | -19.1092 | 2.12E-81 |
| Succinate - Glucose | -0.72208 | -18.3724 | 2.18E-75 |
| Pyruvate - Glucose | -0.74253 | -18.8327 | 4.07E-79 |
| Melibiose - Glucose | -0.76751 | -19.6541 | 5.33E-86 |
| Fumarate - Glucose | -0.56099 | -13.4216 | 4.52E-41 |
| Malate - Glucose | -0.69882 | -17.6601 | 8.51E-70 |
| Fructose - Trehalose | -0.67564 | -17.202 | 2.57E-66 |
| Lactose - Trehalose | -0.67195 | -17.0178 | 6.06E-65 |
| Maltose - Trehalose | -0.70956 | -17.8808 | 1.66E-71 |
| Galactose - Trehalose | -0.66801 | -17.0926 | 1.69E-65 |
| Succinate - Trehalose | -0.68129 | -17.3733 | 1.32E-67 |
| Pyruvate - Trehalose | -0.69091 | -17.6383 | 1.25E-69 |
| Melibiose - Trehalose | -0.67299 | -17.2294 | 1.60E-66 |
| Fumarate - Trehalose | -0.67248 | -16.5612 | 1.33E-61 |
| Malate - Trehalose | -0.66017 | -16.7082 | 1.14E-62 |
| Lactose - Fructose | -0.54603 | -14.1014 | 3.72E-45 |
| Maltose - Fructose | -0.50769 | -12.9881 | 1.43E-38 |
| Galactose - Fructose | -0.55117 | -14.3659 | 8.47E-47 |
| Succinate - Fructose | -0.52782 | -13.7435 | 5.57E-43 |
| Pyruvate - Fructose | -0.55928 | -14.5544 | 5.48E-48 |
| Melibiose - Fructose | -0.53723 | -14.0284 | 1.04E-44 |
| Fumarate - Fructose | -0.48466 | -12.1058 | 9.84E-34 |
| Malate - Fructose | -0.54769 | -14.1953 | 9.80E-46 |
| Maltose - Lactose | -0.70131 | -17.6633 | 8.04E-70 |
| Galactose - Lactose | -0.704 | -18.0102 | 1.62E-72 |
| Succinate - Lactose | -0.6924 | -17.7161 | 3.15E-70 |
| Pyruvate - Lactose | -0.68748 | -17.6157 | 1.87E-69 |
| Melibiose - Lactose | -0.69757 | -17.921 | 8.08E-72 |
| Fumarate - Lactose | -0.6216 | -15.3056 | 7.02E-53 |
| Malate - Lactose | -0.69295 | -17.6859 | 5.38E-70 |
| Galactose - Maltose | -0.77625 | -19.7425 | 9.30E-87 |
| Succinate - Maltose | -0.77074 | -19.596 | 1.67E-85 |
| Pyruvate - Maltose | -0.77911 | -19.7515 | 7.78E-87 |
| Melibiose - Maltose | -0.79308 | -20.2227 | 6.18E-91 |
| Fumarate - Maltose | -0.63421 | -15.1784 | 4.92E-52 |
| Malate - Maltose | -0.75889 | -19.156 | 8.63E-82 |
| Succinate - Galactose | 0.194641 | 5.029628 | 4.91E-07 |
| Pyruvate - Galactose | 0.190352 | 4.919177 | 8.69E-07 |
| Melibiose - Galactose | 0.242794 | 6.266049 | 3.70E-10 |
| Fumarate - Galactose | 0.138841 | 3.449028 | 0.000563 |
| Malate - Galactose | 0.215856 | 5.53914 | 3.04E-08 |
| Pyruvate - Succinate | -0.8189 | -20.4896 | 2.66E-93 |
| Melibiose - Succinate | -0.83318 | -20.8801 | 8.12E-97 |
| Fumarate - Succinate | -0.75217 | -18.3773 | 2.00E-75 |
| Malate - Succinate | -0.8149 | -20.3615 | 3.67E-92 |
| Melibiose - Pyruvate | -0.62428 | -16.2646 | 1.76E-59 |
| Fumarate - Pyruvate | -0.59193 | -14.7499 | 3.08E-49 |
| Malate - Pyruvate | -0.62204 | -16.0602 | 4.85E-58 |
| Fumarate - Melibiose | -0.49718 | -12.3774 | 3.47E-35 |
| Malate - Melibiose | -0.5765 | -14.8919 | 3.72E-50 |
| Malate - Fumarate | -0.80092 | -19.3905 | 9.28E-84 |

**S12 Table.** Kendall’s rank correlation between fitness effect bin and proportion of SP among all mutations. Model: cor.test(resource$fitness_effect_bin, resource$SP_prop, method="kendall").

| **Kendall's Rank correlation** | **Kendall's tau** | **P** |
| --- | --- | --- |
| Proportion of SP vs. fitness effect in Glucose | 0.494 | 4.68E-13 |
| Proportion of SP vs. fitness effect in Trehalose | 0.171 | 0.00037 |
| Proportion of SP vs. fitness effect in Fructose | 0.317 | 4.68E-13 |
| Proportion of SP vs. fitness effect in Lactose | 0.461 | 0 |
| Proportion of SP vs. fitness effect in Maltose | 0.470 | 7.02E-14 |
| Proportion of SP vs. fitness effect in Galactose | 0.228 | 1.18E-06 |
| Proportion of SP vs. fitness effect in Succinate | 0.189 | 8.07E-05 |
| Proportion of SP vs. fitness effect in Pyruvate | 0.376 | 3.49E-14 |
| Proportion of SP vs. fitness effect in Melibiose | 0.149 | 0.000701 |
| Proportion of SP vs. fitness effect in Fumarate | -0.079 | 0.344083 |
| Proportion of SP vs. fitness effect in Malate | 0.141 | 0.015255 |

**S13 Table.** Kendall’s rank correlation between fitness effect size and proportion of SP among mutations that show SP. Model: cor.test(resource$fitness_effect_bin, resource$SP_prop, method="kendall").

| **Kendall's Rank correlation** | **Kendall's tau** | **P** |
| --- | --- | --- |
| Proportion of SP vs. fitness effect in Glucose | 0.024 | 0.682 |
| Proportion of SP vs. fitness effect in Trehalose | 0.112 | 0.007 |
| Proportion of SP vs. fitness effect in Fructose | 0.074 | 0.057 |
| Proportion of SP vs. fitness effect in Lactose | 0.207 | 0.000 |
| Proportion of SP vs. fitness effect in Maltose | 0.063 | 0.246 |
| Proportion of SP vs. fitness effect in Galactose | 0.069 | 0.103 |
| Proportion of SP vs. fitness effect in Succinate | 0.109 | 0.007 |
| Proportion of SP vs. fitness effect in Pyruvate | 0.034 | 0.433 |
| Proportion of SP vs. fitness effect in Melibiose | 0.062 | 0.118 |
| Proportion of SP vs. fitness effect in Fumarate | 0.021 | 0.767 |
| Proportion of SP vs. fitness effect in Malate | 0.193 | 0.000 |

**S14 Table.** Kendall’s rank correlation for the effect of fitness effect size on the proportion of SP in a null distribution of proportion of SP generated by randomly picking fitness values from the DFEs of each resource for each resource pair. Model_correlation: cor.test(resource$fitness_effect_bin, resource$null_SP_prop, method="kendall").

| **Resource pair** | **Estimate** | **Statistic** | **P** |
| --- | --- | --- | --- |
| Trehalose - Glucose | -0.60372 | -15.2626 | 1.36E-52 |
| Fructose - Glucose | -0.63597 | -16.1788 | 7.11E-59 |
| Lactose - Glucose | -0.61422 | -15.5502 | 1.59E-54 |
| Maltose - Glucose | -0.54649 | -13.6323 | 2.57E-42 |
| Galactose - Glucose | -0.61511 | -15.6926 | 1.70E-55 |
| Succinate - Glucose | -0.63157 | -16.1033 | 2.42E-58 |
| Pyruvate - Glucose | -0.60764 | -15.5096 | 2.99E-54 |
| Melibiose - Glucose | -0.64577 | -16.5421 | 1.83E-61 |
| Fumarate - Glucose | -0.47599 | -11.5022 | 1.29E-30 |
| Malate - Glucose | -0.57438 | -14.5933 | 3.10E-48 |
| Fructose - Trehalose | -0.61785 | -15.7945 | 3.40E-56 |
| Lactose - Trehalose | -0.63009 | -16.0038 | 1.20E-57 |
| Maltose - Trehalose | -0.60844 | -15.3138 | 6.19E-53 |
| Galactose - Trehalose | -0.67366 | -17.1701 | 4.45E-66 |
| Succinate - Trehalose | -0.63961 | -16.3934 | 2.13E-60 |
| Pyruvate - Trehalose | -0.62443 | -15.967 | 2.17E-57 |
| Melibiose - Trehalose | -0.64592 | -16.6582 | 2.64E-62 |
| Fumarate - Trehalose | -0.57865 | -14.1741 | 1.33E-45 |
| Malate - Trehalose | -0.60976 | -15.5442 | 1.74E-54 |
| Lactose - Fructose | -0.76254 | -19.7057 | 1.93E-86 |
| Maltose - Fructose | -0.74146 | -19.0369 | 8.43E-81 |
| Galactose - Fructose | -0.79797 | -20.7041 | 3.18E-95 |
| Succinate - Fructose | -0.78298 | -20.3465 | 4.98E-92 |
| Pyruvate - Fructose | -0.7795 | -20.2805 | 1.91E-91 |
| Melibiose - Fructose | -0.79934 | -20.877 | 8.67E-97 |
| Fumarate - Fructose | -0.6988 | -17.5529 | 5.65E-69 |
| Malate - Fructose | -0.76476 | -19.8108 | 2.40E-87 |
| Maltose - Lactose | -0.64749 | -16.3244 | 6.62E-60 |
| Galactose - Lactose | -0.70399 | -18.0923 | 3.67E-73 |
| Succinate - Lactose | -0.69728 | -17.9029 | 1.12E-71 |
| Pyruvate - Lactose | -0.67117 | -17.2413 | 1.30E-66 |
| Melibiose - Lactose | -0.70667 | -18.2743 | 1.33E-74 |
| Fumarate - Lactose | -0.62758 | -15.5183 | 2.61E-54 |
| Malate - Lactose | -0.68014 | -17.397 | 8.69E-68 |
| Galactose - Maltose | -0.57986 | -14.7065 | 5.85E-49 |
| Succinate - Maltose | -0.61099 | -15.5269 | 2.28E-54 |
| Pyruvate - Maltose | -0.58558 | -14.8837 | 4.20E-50 |
| Melibiose - Maltose | -0.58969 | -15.0847 | 2.04E-51 |
| Fumarate - Maltose | -0.42032 | -10.0937 | 5.89E-24 |
| Malate - Maltose | -0.58805 | -14.8187 | 1.11E-49 |
| Succinate - Galactose | -0.84844 | -22.3992 | 4.00E-111 |
| Pyruvate - Galactose | -0.8588 | -22.6273 | 2.33E-113 |
| Melibiose - Galactose | -0.86273 | -22.7858 | 6.35E-115 |
| Fumarate - Galactose | -0.7654 | -19.7131 | 1.67E-86 |
| Malate - Galactose | -0.83426 | -21.9605 | 6.88E-107 |
| Pyruvate - Succinate | -0.50253 | -12.811 | 1.42E-37 |
| Melibiose - Succinate | -0.48734 | -12.5591 | 3.54E-36 |
| Fumarate - Succinate | -0.47815 | -11.6253 | 3.07E-31 |
| Malate - Succinate | -0.51655 | -13.0173 | 9.76E-39 |
| Melibiose - Pyruvate | -0.76675 | -19.9262 | 2.41E-88 |
| Fumarate - Pyruvate | -0.70611 | -17.5698 | 4.19E-69 |
| Malate - Pyruvate | -0.74033 | -19.1573 | 8.41E-82 |
| Fumarate - Melibiose | -0.64455 | -16.0588 | 4.96E-58 |
| Malate - Melibiose | -0.71184 | -18.3367 | 4.21E-75 |
| Malate - Fumarate | -0.29616 | -7.32282 | 2.43E-13 |

**S15 Table.** Spearman’s two-sided rank correlation for effect sizes of mutations showing AP across all resource pairs. Model: cor.test(abs(focal_resource_fitness), abs(other_resource_fitness), method = "spearman", exact = F, conf.level = 0.95).

| **Comparison** | **Spearman's rho** | **P** | **n** |
| --- | --- | --- | --- |
| Glucose - LB | 0.288 | 0.279 | 16 |
| Trehalose - LB | 0.054 | 0.811 | 22 |
| Fructose - LB | 0.026 | 0.903 | 25 |
| Lactose - LB | -0.010 | 0.963 | 23 |
| Maltose - LB | -0.382 | 0.221 | 12 |
| Galactose - LB | 0.055 | 0.775 | 30 |
| Succinate - LB | 0.275 | 0.183 | 25 |
| Pyruvate - LB | 0.417 | 0.096 | 17 |
| Melibiose - LB | -0.005 | 0.980 | 28 |
| Fumarate - LB | -0.283 | 0.460 | 9 |
| Malate - LB | -0.186 | 0.326 | 30 |
| Trehalose - Glucose | NA | NA | 2 |
| Fructose - Glucose | NA | NA | 3 |
| Lactose - Glucose | NA | NA | 5 |
| Maltose - Glucose | NA | NA | 1 |
| Galactose - Glucose | -0.500 | 0.117 | 11 |
| Succinate - Glucose | -0.900 | 0.037 | 5 |
| Pyruvate - Glucose | -0.714 | 0.071 | 7 |
| Melibiose - Glucose | -0.400 | 0.505 | 5 |
| Fumarate - Glucose | -0.714 | 0.111 | 6 |
| Malate - Glucose | -0.434 | 0.138 | 13 |
| Fructose - Trehalose | NA | NA | 1 |
| Lactose - Trehalose | NA | NA | 1 |
| Maltose - Trehalose | NA | NA | 2 |
| Galactose - Trehalose | -0.500 | 0.170 | 9 |
| Succinate - Trehalose | NA | NA | 0 |
| Pyruvate - Trehalose | -0.758 | 0.011 | 10 |
| Melibiose - Trehalose | NA | NA | 0 |
| Fumarate - Trehalose | -0.257 | 0.623 | 6 |
| Malate - Trehalose | -0.656 | 0.001 | 22 |
| Lactose - Fructose | NA | NA | 1 |
| Maltose - Fructose | -0.810 | 0.015 | 5 |
| Galactose - Fructose | NA | NA | 8 |
| Succinate - Fructose | NA | NA | 2 |
| Pyruvate - Fructose | -0.685 | 0.014 | 12 |
| Melibiose - Fructose | 0.500 | 0.667 | 3 |
| Fumarate - Fructose | -0.800 | 0.010 | 9 |
| Malate - Fructose | -0.509 | 0.013 | 23 |
| Maltose - Lactose | -0.857 | 0.007 | 8 |
| Galactose - Lactose | -0.467 | 0.205 | 9 |
| Succinate - Lactose | -0.829 | 0.042 | 6 |
| Pyruvate - Lactose | -0.632 | 0.009 | 16 |
| Melibiose - Lactose | -0.071 | 0.879 | 7 |
| Fumarate - Lactose | -0.881 | 0.004 | 8 |
| Malate - Lactose | -0.546 | 0.011 | 21 |
| Galactose - Maltose | -0.802 | 0.001 | 13 |
| Succinate - Maltose | NA | NA | 4 |
| Pyruvate - Maltose | -0.900 | 0.037 | 5 |
| Melibiose - Maltose | NA | NA | 4 |
| Fumarate - Maltose | NA | NA | 3 |
| Malate - Maltose | -0.493 | 0.045 | 17 |
| Succinate - Galactose | -0.661 | 0.038 | 10 |
| Pyruvate - Galactose | -0.708 | 0.000 | 20 |
| Melibiose - Galactose | -0.382 | 0.247 | 11 |
| Fumarate - Galactose | -0.890 | 0.000 | 14 |
| Malate - Galactose | -0.625 | 0.013 | 15 |
| Pyruvate - Succinate | -0.636 | 0.048 | 10 |
| Melibiose - Succinate | NA | NA | 0 |
| Fumarate - Succinate | -0.267 | 0.488 | 9 |
| Malate - Succinate | -0.636 | 0.011 | 15 |
| Melibiose - Pyruvate | -0.758 | 0.011 | 10 |
| Fumarate - Pyruvate | -0.286 | 0.493 | 8 |
| Malate - Pyruvate | -0.744 | 0.000 | 24 |
| Fumarate - Melibiose | -0.543 | 0.266 | 6 |
| Malate - Melibiose | -0.476 | 0.014 | 26 |
| Malate - Fumarate | -0.648 | 0.043 | 10 |

**S16 Table.** Spearman’s two-sided rank correlation for effect sizes of mutations showing SP across all resource pairs. Model: cor.test(abs(focal_resource_fitness), abs(other_resource_fitness), method = "spearman", exact = F, conf.level = 0.95).

| **Comparison** | **Spearman's rho** | **P** | **n** |
| --- | --- | --- | --- |
| Glucose - LB | -0.117 | 0.765008 | 9 |
| Trehalose - LB | 0.098 | 0.708151 | 17 |
| Fructose - LB | 0.046 | 0.852896 | 19 |
| Lactose - LB | 0.335 | 0.204255 | 16 |
| Maltose - LB | -0.305 | 0.28817 | 14 |
| Galactose - LB | 0.105 | 0.687266 | 17 |
| Succinate - LB | 0.120 | 0.646143 | 17 |
| Pyruvate - LB | -0.250 | 0.200237 | 28 |
| Melibiose - LB | 0.161 | 0.509171 | 19 |
| Fumarate - LB | 0.445 | 0.127524 | 13 |
| Malate - LB | -0.462 | 0.112376 | 13 |
| Trehalose - Glucose | 0.884 | 4.96E-08 | 22 |
| Fructose - Glucose | 0.841 | 1.85E-06 | 21 |
| Lactose - Glucose | 0.920 | 1.2E-06 | 15 |
| Maltose - Glucose | 0.910 | 1.03E-09 | 23 |
| Galactose - Glucose | 0.696 | 0.003937 | 15 |
| Succinate - Glucose | 0.838 | 2.17E-06 | 21 |
| Pyruvate - Glucose | 0.828 | 6.56E-06 | 20 |
| Melibiose - Glucose | 0.875 | 5.04E-09 | 26 |
| Fumarate - Glucose | 0.887 | 0.000122 | 12 |
| Malate - Glucose | 0.836 | 0.004954 | 9 |
| Fructose - Trehalose | 0.887 | 4.72E-17 | 48 |
| Lactose - Trehalose | 0.882 | 1.23E-12 | 36 |
| Maltose - Trehalose | 0.839 | 8.43E-08 | 26 |
| Galactose - Trehalose | 0.791 | 5.64E-09 | 37 |
| Succinate - Trehalose | 0.818 | 6.78E-11 | 41 |
| Pyruvate - Trehalose | 0.843 | 1.41E-09 | 32 |
| Melibiose - Trehalose | 0.829 | 1.02E-13 | 50 |
| Fumarate - Trehalose | 0.820 | 0.000181 | 15 |
| Malate - Trehalose | 0.853 | 1.76E-06 | 20 |
| Lactose - Fructose | 0.894 | 7.01E-16 | 43 |
| Maltose - Fructose | 0.802 | 1.04E-06 | 25 |
| Galactose - Fructose | 0.801 | 1.35E-11 | 47 |
| Succinate - Fructose | 0.745 | 2.81E-09 | 46 |
| Pyruvate - Fructose | 0.834 | 1.49E-10 | 37 |
| Melibiose - Fructose | 0.879 | 5.34E-18 | 53 |
| Fumarate - Fructose | 0.707 | 0.004664 | 14 |
| Malate - Fructose | 0.721 | 3.20E-06 | 32 |
| Maltose - Lactose | 0.887 | 9.09E-07 | 18 |
| Galactose - Lactose | 0.836 | 4.02E-10 | 35 |
| Succinate - Lactose | 0.821 | 1.53E-08 | 31 |
| Pyruvate - Lactose | 0.815 | 2.17E-06 | 23 |
| Melibiose - Lactose | 0.795 | 4.12E-09 | 37 |
| Fumarate - Lactose | 0.859 | 0.003032 | 9 |
| Malate - Lactose | 0.798 | 5.16E-06 | 23 |
| Galactose - Maltose | 0.838 | 7.66E-06 | 19 |
| Succinate - Maltose | 0.822 | 4.68E-07 | 25 |
| Pyruvate - Maltose | 0.936 | 2.04E-11 | 24 |
| Melibiose - Maltose | 0.808 | 6.74E-08 | 30 |
| Fumarate - Maltose | 0.850 | 0.000233 | 13 |
| Malate - Maltose | 0.897 | 0.000438 | 10 |
| Succinate - Galactose | 0.755 | 3.20E-09 | 44 |
| Pyruvate - Galactose | 0.735 | 7.43E-07 | 34 |
| Melibiose - Galactose | 0.824 | 6.37E-12 | 44 |
| Fumarate - Galactose | 0.933 | 2.71E-05 | 11 |
| Malate - Galactose | 0.750 | 3.88E-08 | 39 |
| Pyruvate - Succinate | 0.886 | 1.36E-13 | 38 |
| Melibiose - Succinate | 0.774 | 4.41E-11 | 50 |
| Fumarate - Succinate | 0.908 | 1.89E-07 | 18 |
| Malate - Succinate | 0.763 | 5.96E-07 | 31 |
| Melibiose - Pyruvate | 0.824 | 6.59E-12 | 44 |
| Fumarate - Pyruvate | 0.867 | 1.53E-06 | 19 |
| Malate - Pyruvate | 0.763 | 5.80E-06 | 26 |
| Fumarate - Melibiose | 0.871 | 2.60E-06 | 18 |
| Malate - Melibiose | 0.695 | 0.000114 | 25 |
| Malate - Fumarate | 0.844 | 0.000557 | 12 |

**SUPPLEMENTARY FIGURES**

**S1 Fig. Measured fitness of experimental isolates is consistent across two experimental runs.** Each panel shows the linear regression fit (dashed line) for growth rates obtained in two independent experimental measurements for a subset of 40 randomly picked mutants for three resources.

**
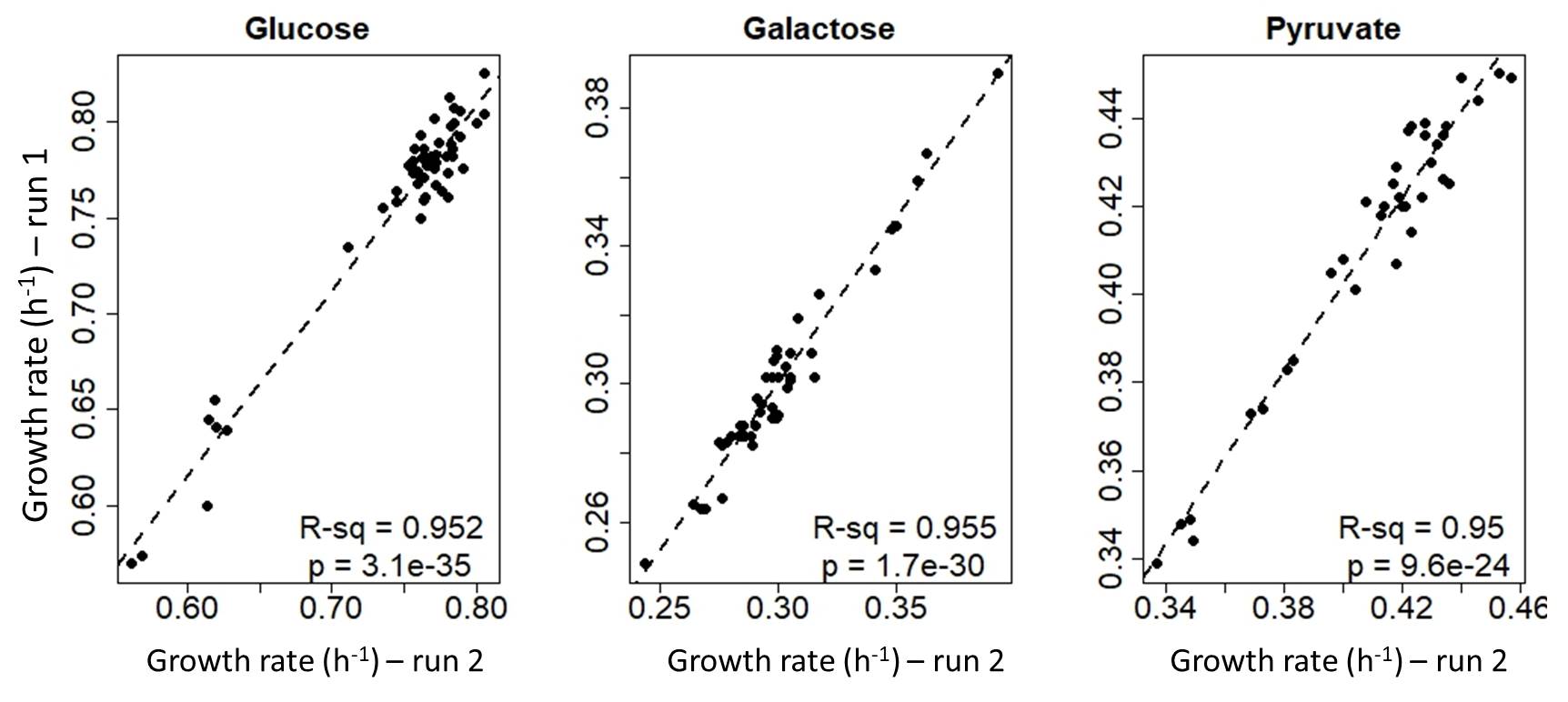
**

**S2 Fig. Distribution of fitness effects of new mutations.** Histograms show the distribution of relative fitness effects of all mutations in each resource (*n* = 80 mutants per panel). The solid red line in each panel indicates no change in fitness; dashed red lines indicate the replication error threshold (absolute relative fitness < 0.05; also see Fig. 1). Percentage values on the left and right of the solid red line indicate the percentage of deleterious and beneficial mutations.


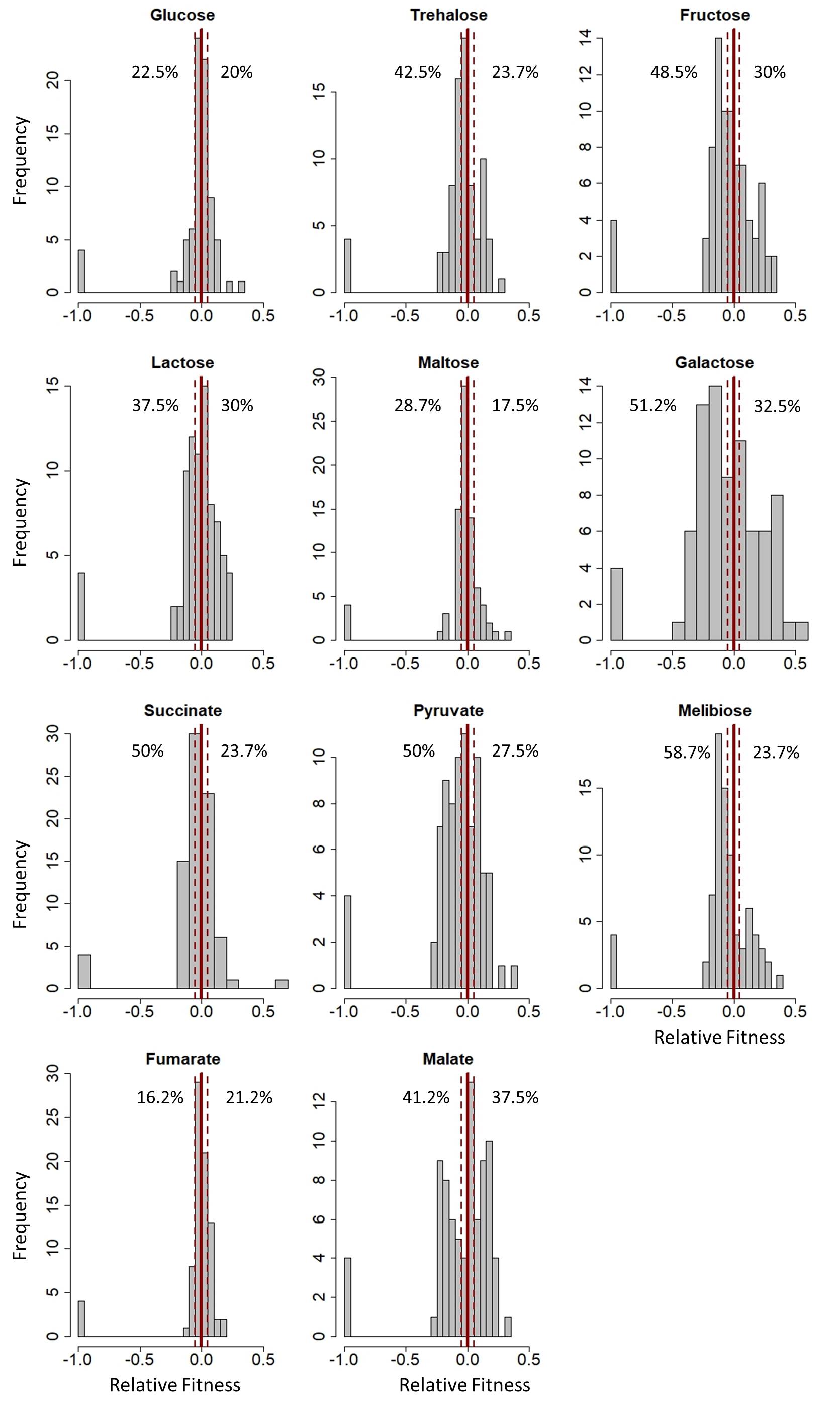


**Supplementary figures S3 to S5 are uploaded as separate PDF files.**

**S3 Fig. Fitness effects of single mutations in each resource.** The heatmap shows the fitness effects of each of the 80 single mutants (y-axis), in each of the 11 resources (x-axis). The color intensity indicates the magnitude of relative fitness change compared to the immediate ancestor.

**S4 Fig. Fitness effects of single mutations in all 55 resource pairs.** Each panel shows the relative fitness of each of the 80 single mutants in all resource pairs. Positive values indicate an increase in fitness compared to the immediate mutational ancestor; negative values indicate a decrease in fitness compared to the immediate mutational ancestor. Lethal mutations are colored red. Also see S5 Fig.

**S5 Fig. Fitness effects of non-lethal single mutations in all 55 resource pairs.** Each panel shows fitness effects of each of the 76 non-lethal single mutants in all possible resource pairs (i.e. excluding the red points from S4 Fig). Positive values indicate an increase in fitness compared to the immediate mutational ancestor; negative values indicate a decrease in fitness compared to the immediate mutational ancestor.

**S6 Fig. Incidence of synergistic pleiotropy (SP) and antagonistic pleiotropy (AP) across resources.** Proportion of mutants showing (A) AP and (B) SP, for each focal resource. Boxplots show the median proportion of SP or AP between the focal resource and all other resources (*n* = 80 mutants). Each colored point represents the proportion of SP or AP for a specific resource paired with the focal resource (see color key). Overlapping points are scattered along the x-axis for clarity.

**
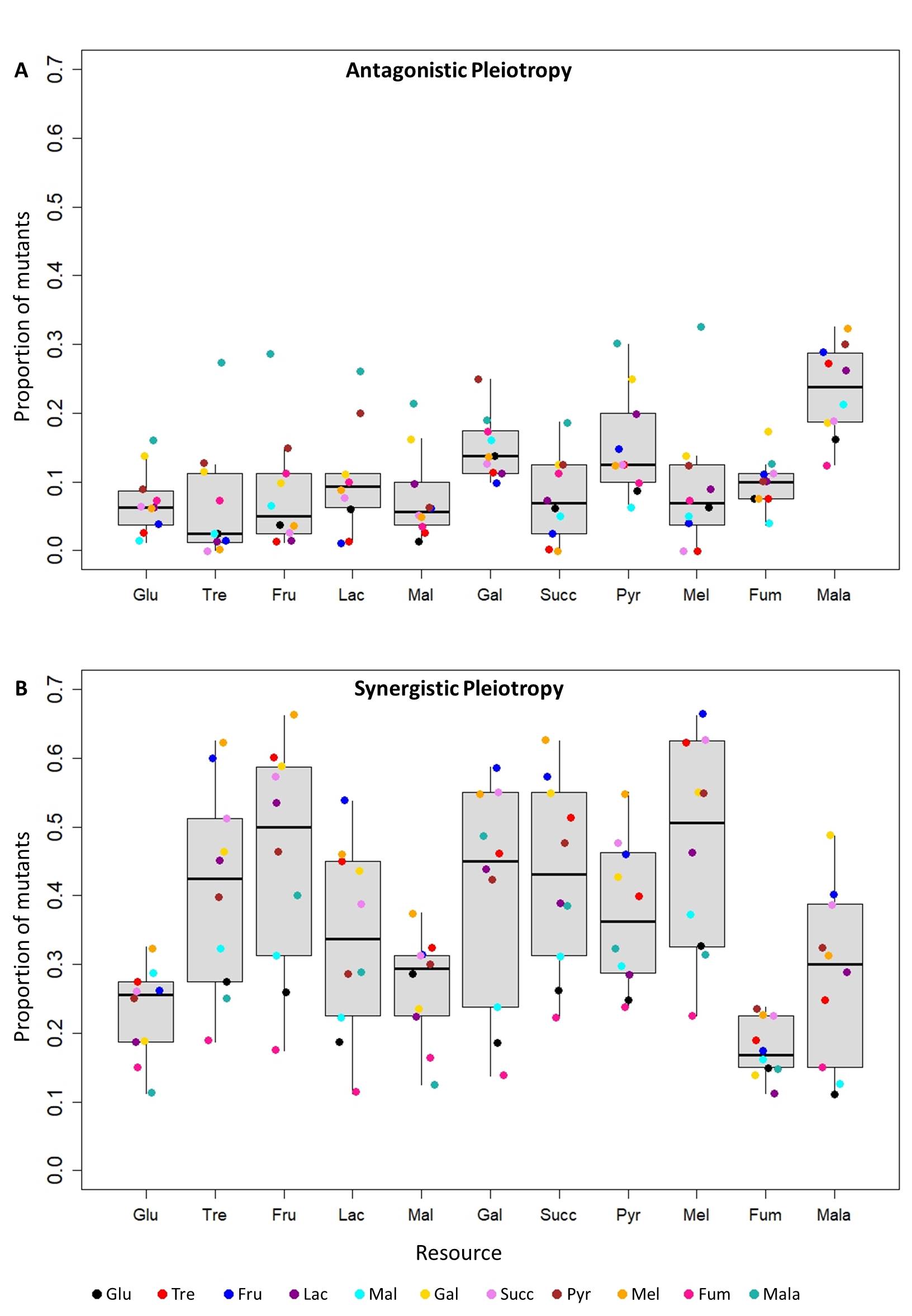
**

**S7 Fig. Comparing the incidence of pleiotropy across first-step and second-step mutations.** In the first panel, bars show the number of mutants (out of 80) that represent the 1^st^, 2^nd^, 3^rd^ or 4^th^ mutational step in their respective MA line. In subsequent panels, boxplots show the median proportion of AP between the focal resource and all other resources, for first-step mutations (*n* = 33) vs. second-step mutations (*n* = 29). Each point represents the proportion of AP for the focal resource (indicated in each panel) paired with another resource (see color key at the bottom). Overlapping points are slightly scattered along the x-axis for clarity. Asterisks indicate a significant difference between the proportion of AP in first- vs. second-step mutations (in fumarate and malate; p <0.05, generalized linear model with binomial errors; see S7 Table).


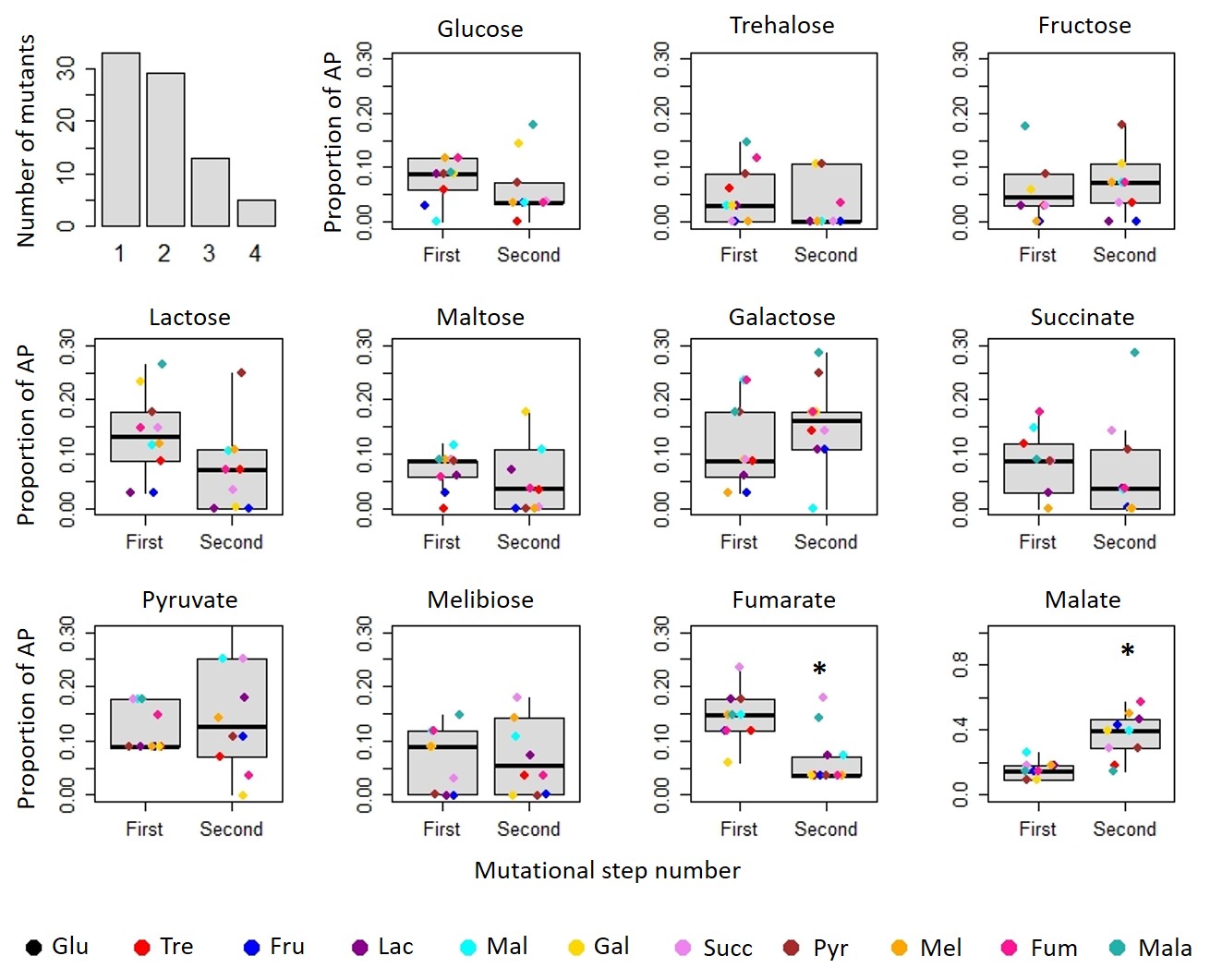


**S8 Fig.** **Frequency of pleiotropy across resource pairs.** Histograms show the number of mutants showing a given type of fitness effect in a given number of resource pairs (x-axis) (*n* = 80 mutants). (A) synergistic decreases, (B) synergistic increases (C) Antagonistic pleiotropy, and (D) no fitness change.

**
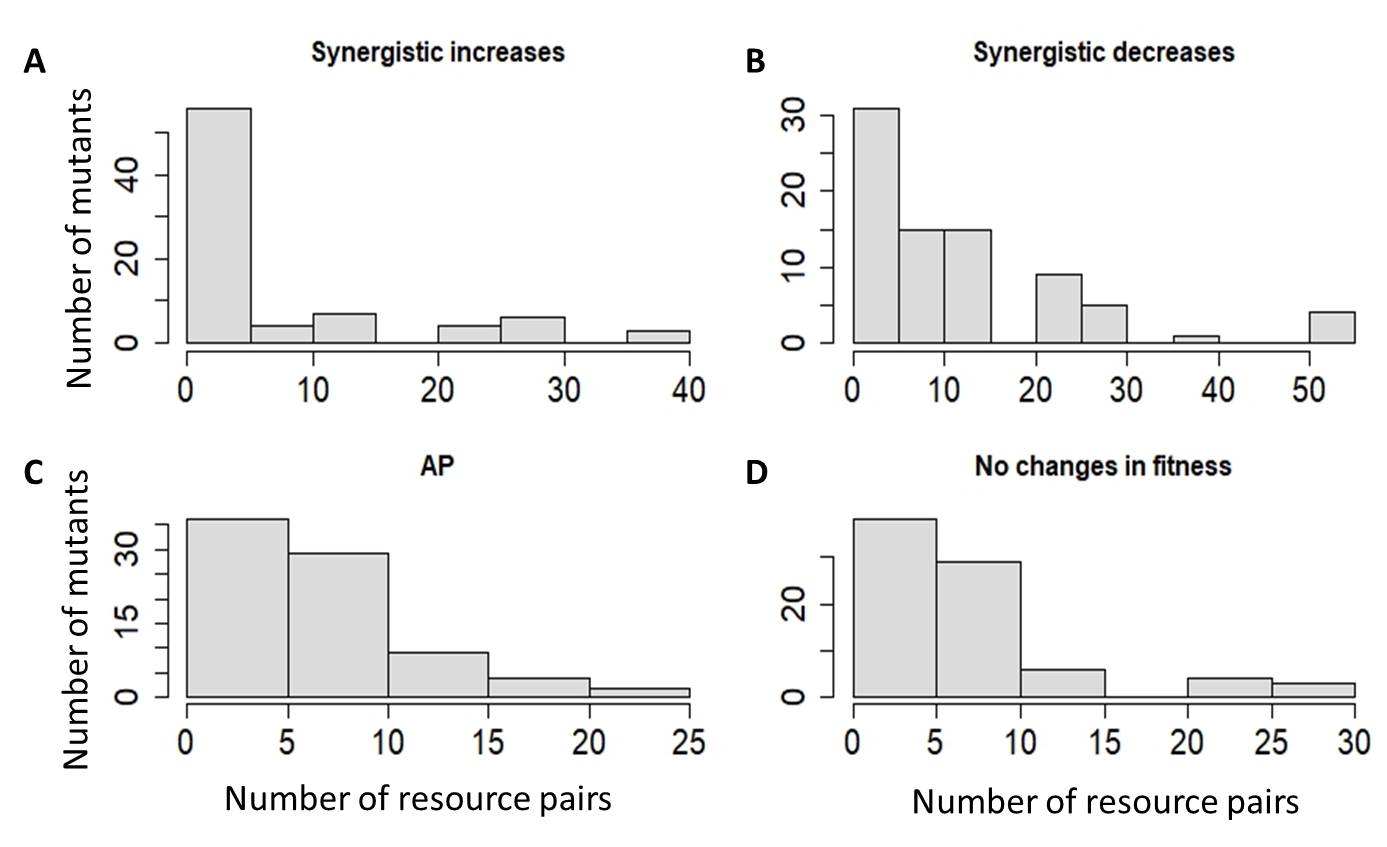
**

**S9 Fig. Functional classification of mutations showing AP.** The histogram shows the number of mutants exhibiting AP for a given number of resource pairs. Numbers in each bar indicate the exact number of mutations showing AP for that particular category. Pie charts show the proportion of AP-causing mutations in each category that correspond to the listed GO “molecular function” categories. GO categories of observed mutations are not significantly different than expected based on the number of genes in each category in the GO database (p > 0.05, chi-squared test, see S8 Table).

**
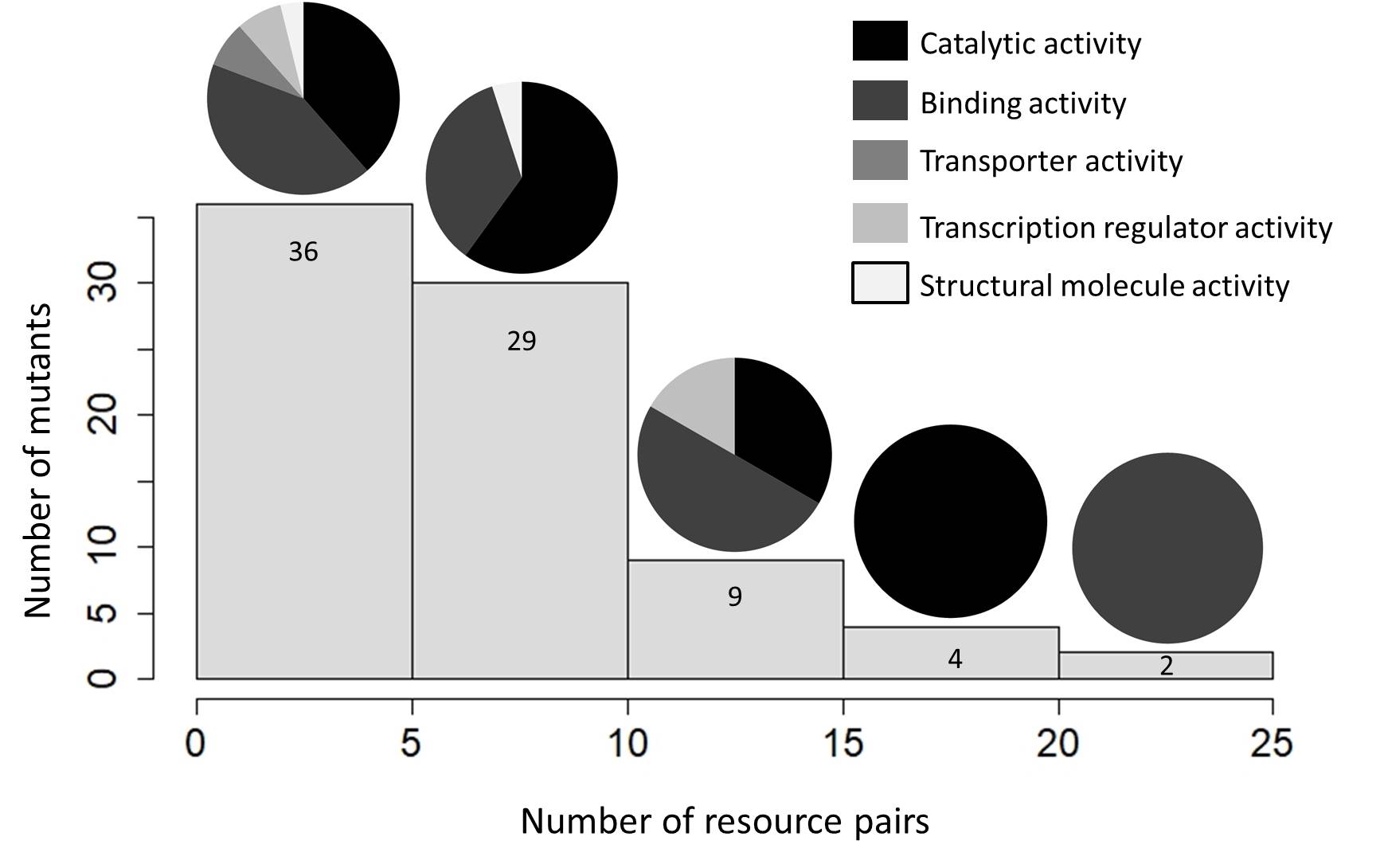
**

**S10 Fig. Relationship between the incidence of SP and fitness effect size.** (A) Proportion of mutants showing SP in each fitness effect size class. For each mutant, we classified measured fitness effects for the focal resource into four effect size classes, and then calculated the proportion of measurements representing SP with any other resource. Each plot thus represents a total of 80 measurements, of which *n* (indicated in each plot) represent SP. (B) Distribution of SP-causing mutations across fitness effect size classes. We filtered all instances of SP across our dataset (total 2648), of which *n* (indicated in each plot) represent data for a given focal resource. For each resource, we calculated the proportion of measurements belonging to each fitness effect size class. Plot titles in red indicate a significant correlation between the fitness effect size bin and the incidence of SP (p <0.05, Kendall’s rank correlation; see inset p values in each panel; also see Tables S12 and S13). Corresponding results for the correlation between expected SP incidence (based on null distributions) and fitness effect size are shown in Fig. S16.

**
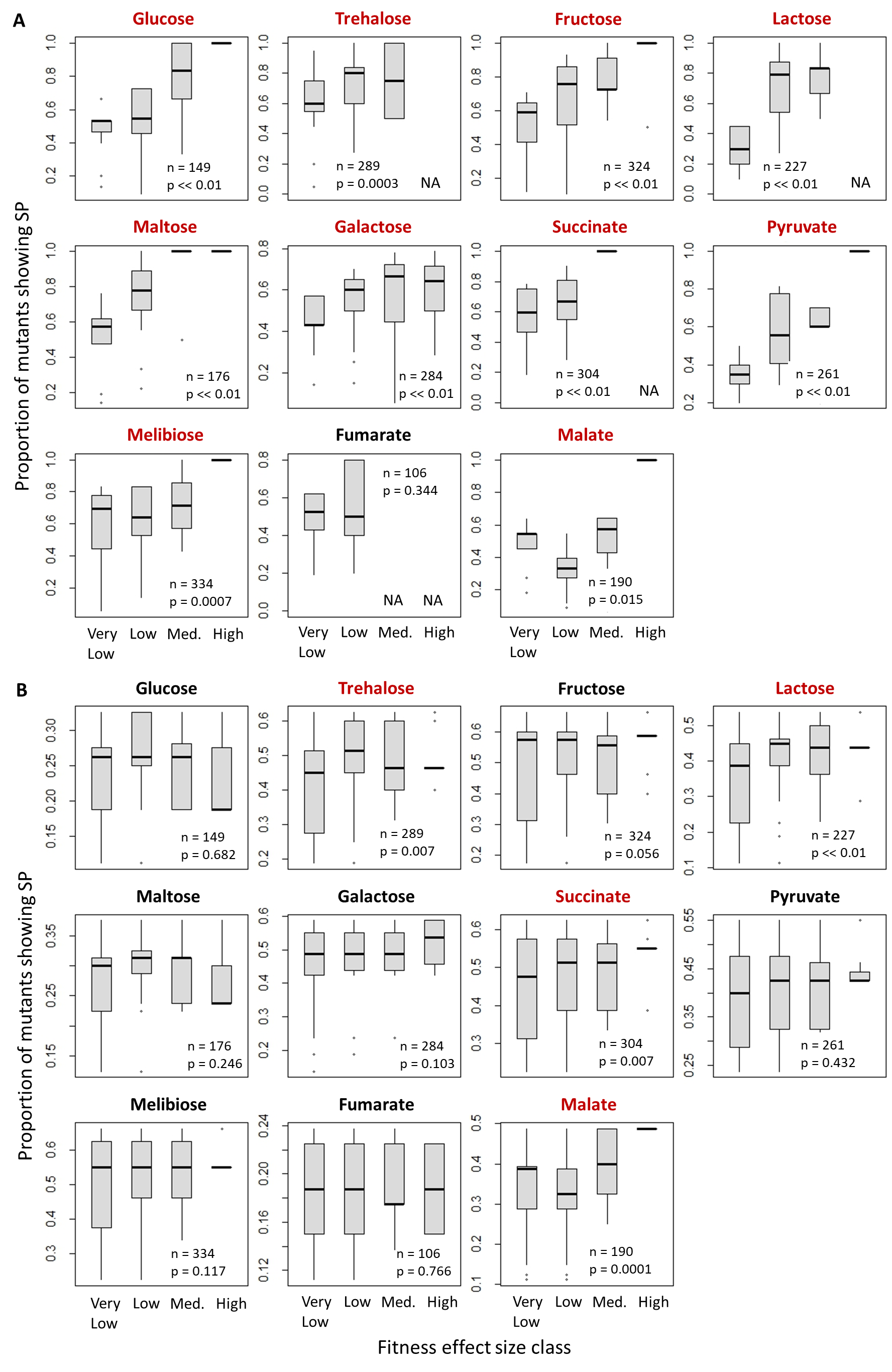
**

**Supplementary figures S11 to S16 are uploaded as separate PDF files.**

**S11 Fig. Observed proportion of AP is less than the null expectation in most resource pairs.** Each panel shows the null distribution for the expected proportion of AP for one of 55 resource pairs, generated by randomly picking fitness values from the DFE of each resource in the pair. The red line in each panel indicates the mean expected proportion of AP for the particular resource pair; the blue line indicates the observed proportion of AP for that resource pair. p values are for a One-sample Student’s t-test comparing the observed and expected proportions of AP (also see Table 1).

**S12 Fig. Observed proportion of SP is greater than the null expectation in most resource pairs.** Each panel shows the null distribution for the expected proportion of SP for one of 55 resource pairs, generated by randomly picking fitness values from the DFE of each resource in the pair. The red line in each panel indicates the mean expected proportion of SP for the particular resource pair; the blue line indicates the observed proportion of SP for that resource pair. p values are for a One-sample Student’s t-test comparing the observed and expected proportions of SP (also see Table 1).

**S13 Fig. Observed proportion of AP among beneficial mutations is less than the null expectation in most resource pairs.** Each panel shows the null distribution for the expected proportion of AP for one of 110 resource pairs, generated by randomly picking only beneficial fitness values from the DFE of one of the resources in the pair. The red line in each panel indicates the mean expected proportion of AP for the particular resource pair; the blue line indicates the observed proportion of AP for that resource pair. p values in the top right are for a One-sample Student’s t-test comparing the observed and expected proportions of AP (also see Table 1).

**S14 Fig. Observed proportion of SP among beneficial mutations is less than the null expectation in most resource pairs.** Each panel shows the null distribution for the expected proportion of SP for one of 110 resource pairs, generated by randomly picking only beneficial fitness values from the DFE of one of the resources in the pair. The red line in each panel indicates the mean expected proportion of SP for the particular resource pair; the blue line indicates the observed proportion of SP for that resource pair. p values in the top right are for a One-sample Student’s t-test comparing the observed and expected proportions of SP (also see Table 1).

**S15 Fig. Expected proportion of AP is negatively correlated with fitness effect size.** Each panel shows the correlation between the expected proportion of AP and fitness effect size for one of 55 resource pairs, generated by randomly picking fitness values from the DFE of each resource in the pair. p values in the top right of each panel are for a Kendall’s rank correlation (Kendall’s tau values also at the top right of each panel) between proportion of AP and fitness class (also see Table S11).

**S16 Fig. Expected proportion of SP is negatively correlated with fitness effect size.** Each panel shows the correlation between the expected proportion of SP and fitness effect size for one of 55 resource pairs, generated by randomly picking fitness values from the DFE of each resource in the pair. p values in the top right of each panel are for a Kendall’s rank correlation (Kendall’s tau values also at the top right of each panel) between proportion of SP and fitness class (also see Table S14).
