# Supplementary figures and images for: Antagonistic pleiotropy for carbon use is rare in new mutations
Source: Evolution. 2018 Sep 13;72(10):2202–13. doi: 10.1111/evo.13569 (PMC6203952; doi:10.1111/evo.13569)

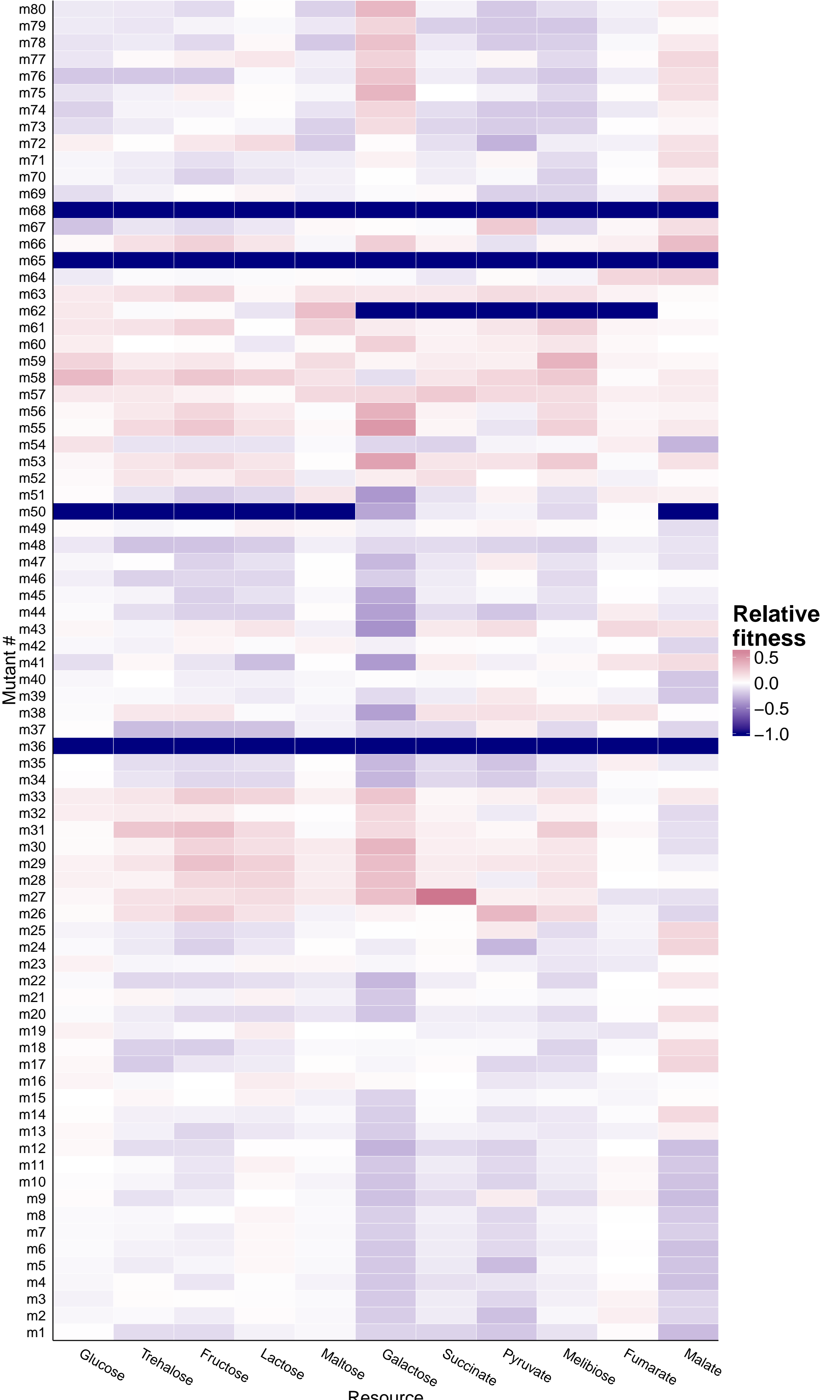

Supplement: Supplementary file 2 [file EVO-72-2202-s002.pdf]

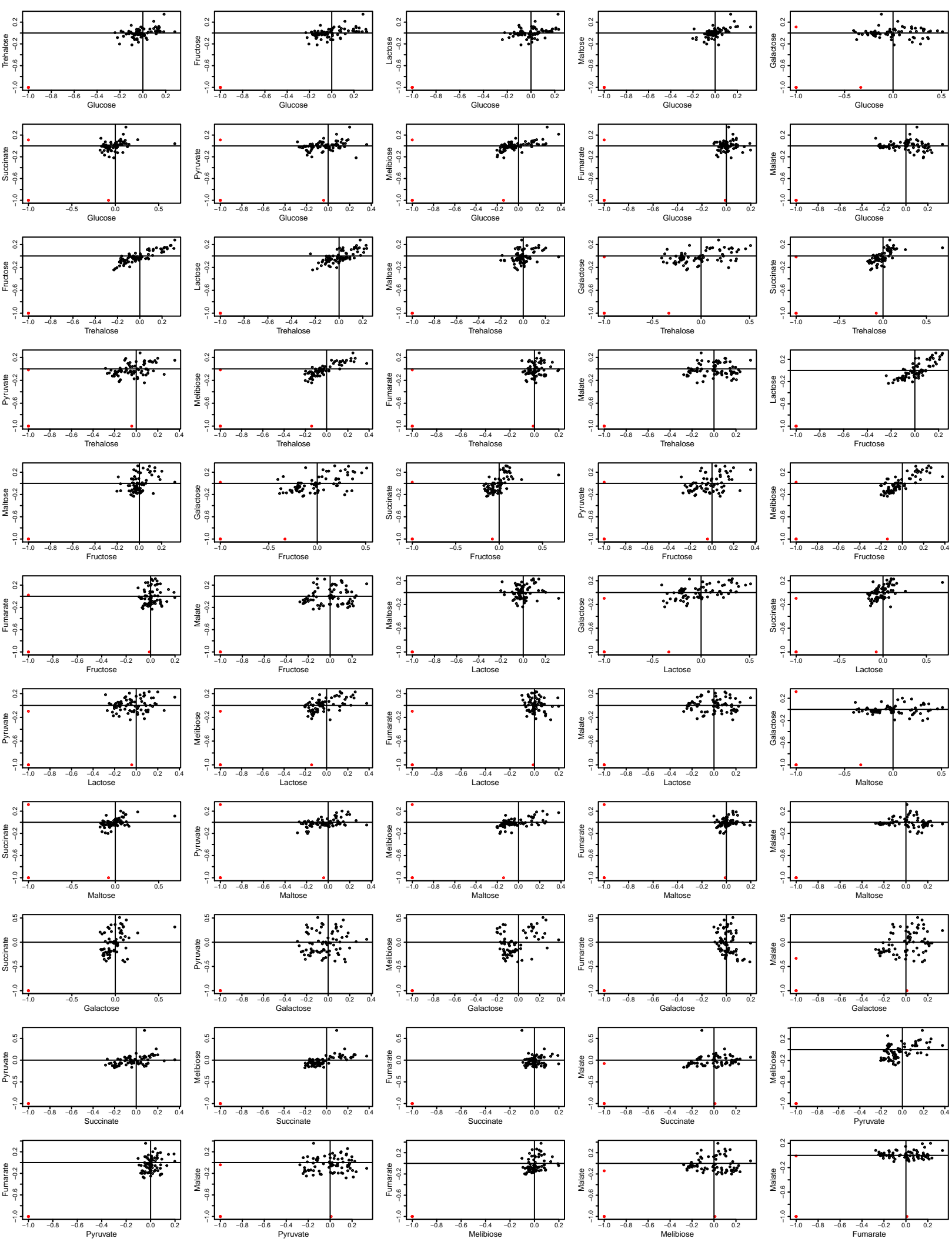

Supplement: Supplementary file 3 [file EVO-72-2202-s003.pdf]

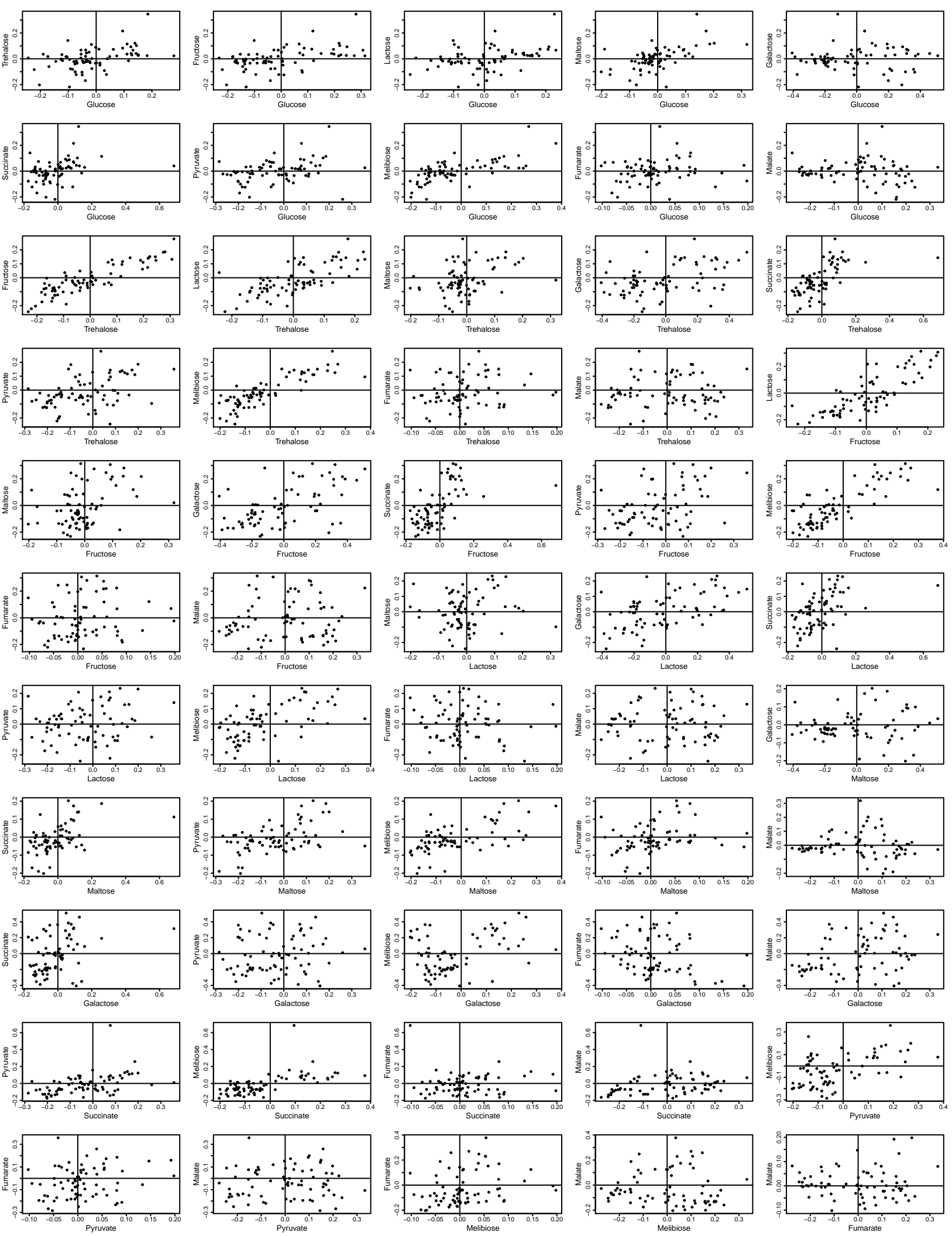

Supplement: Supplementary file 4 [file EVO-72-2202-s004.pdf]

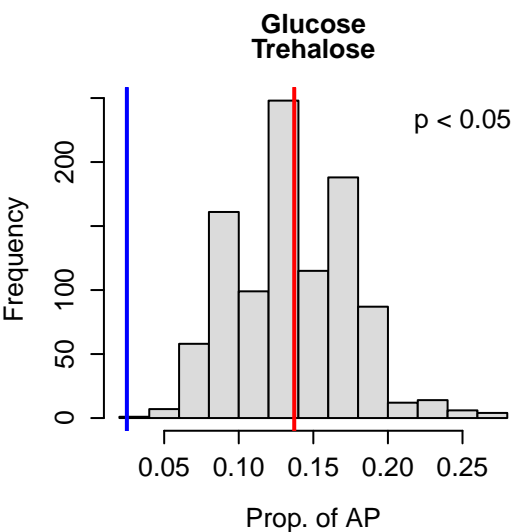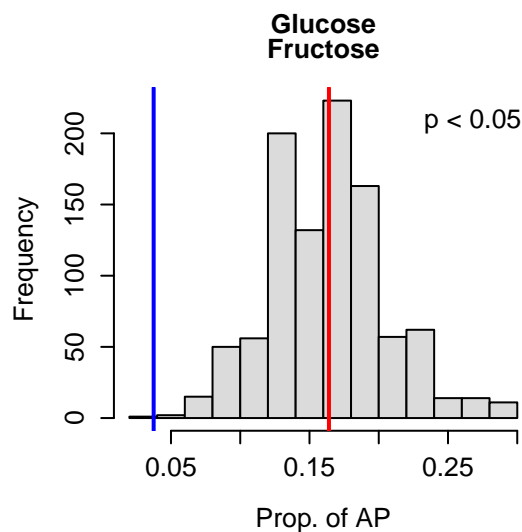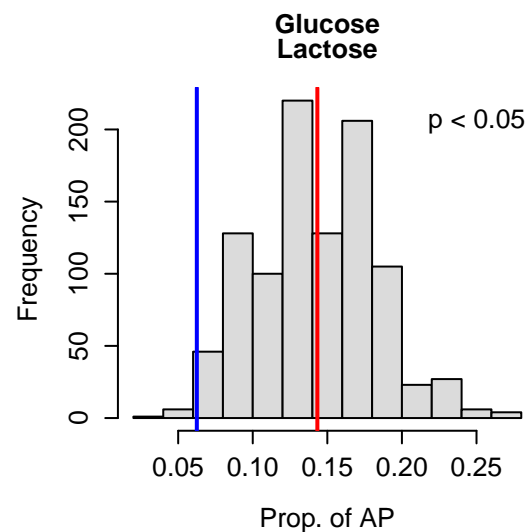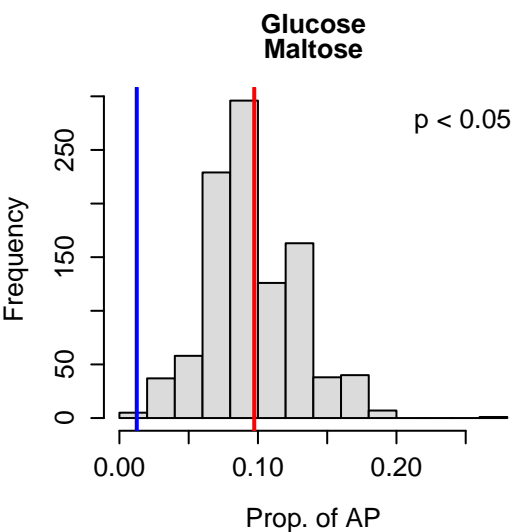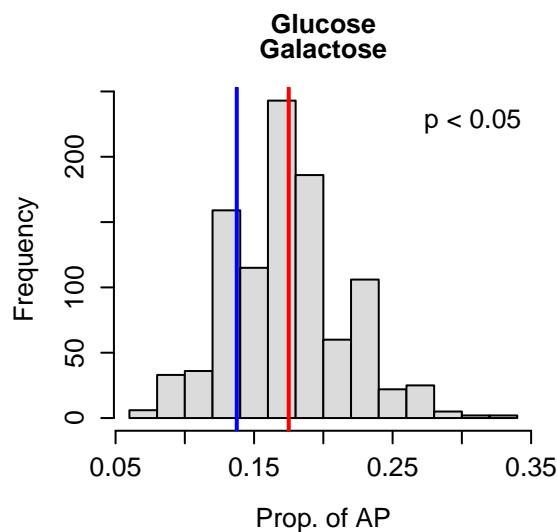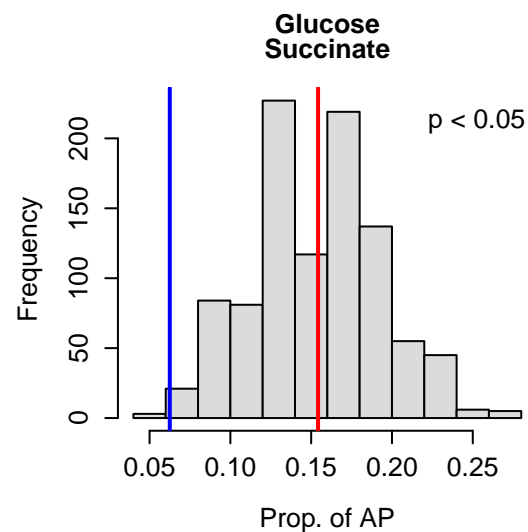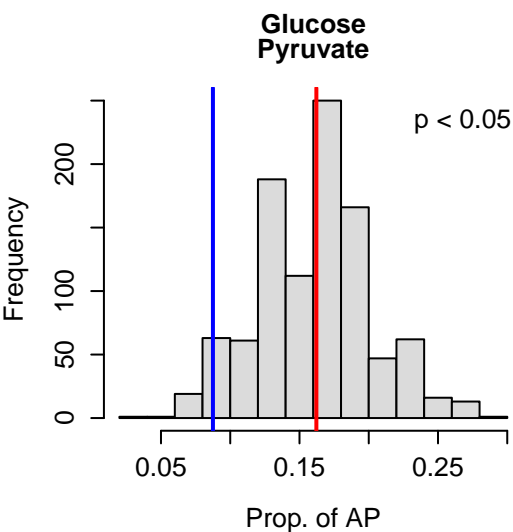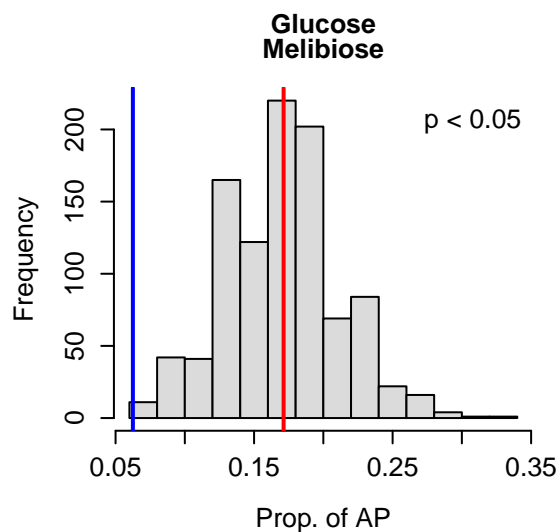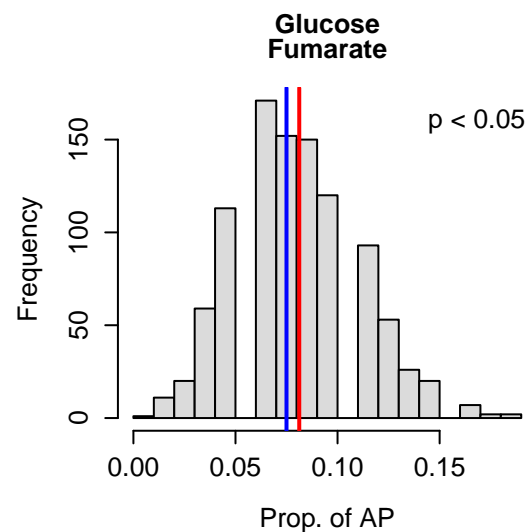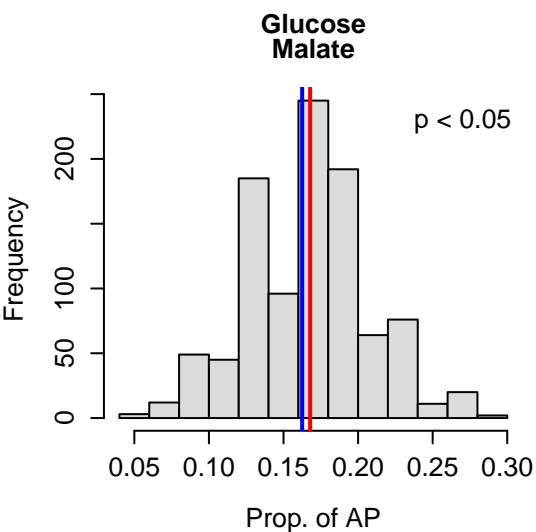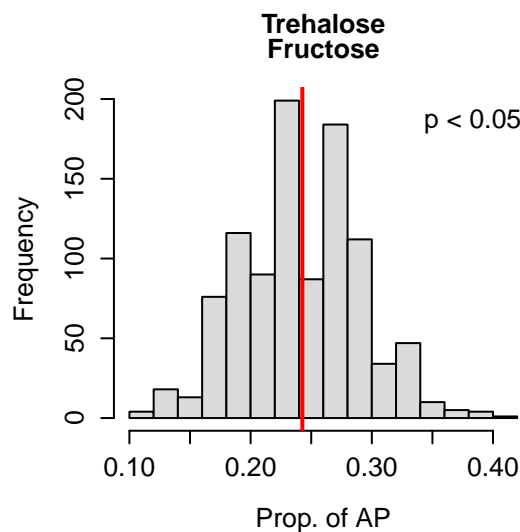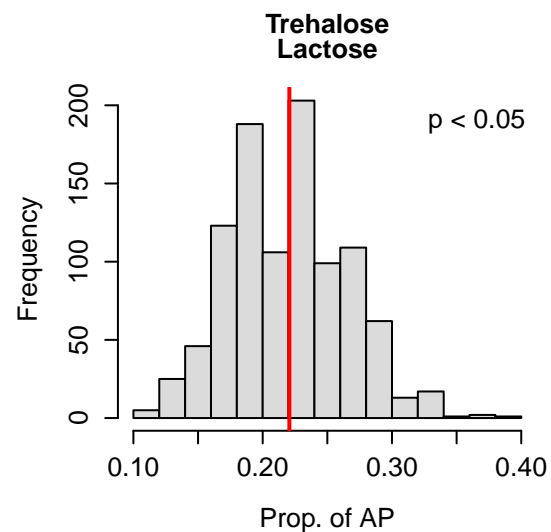

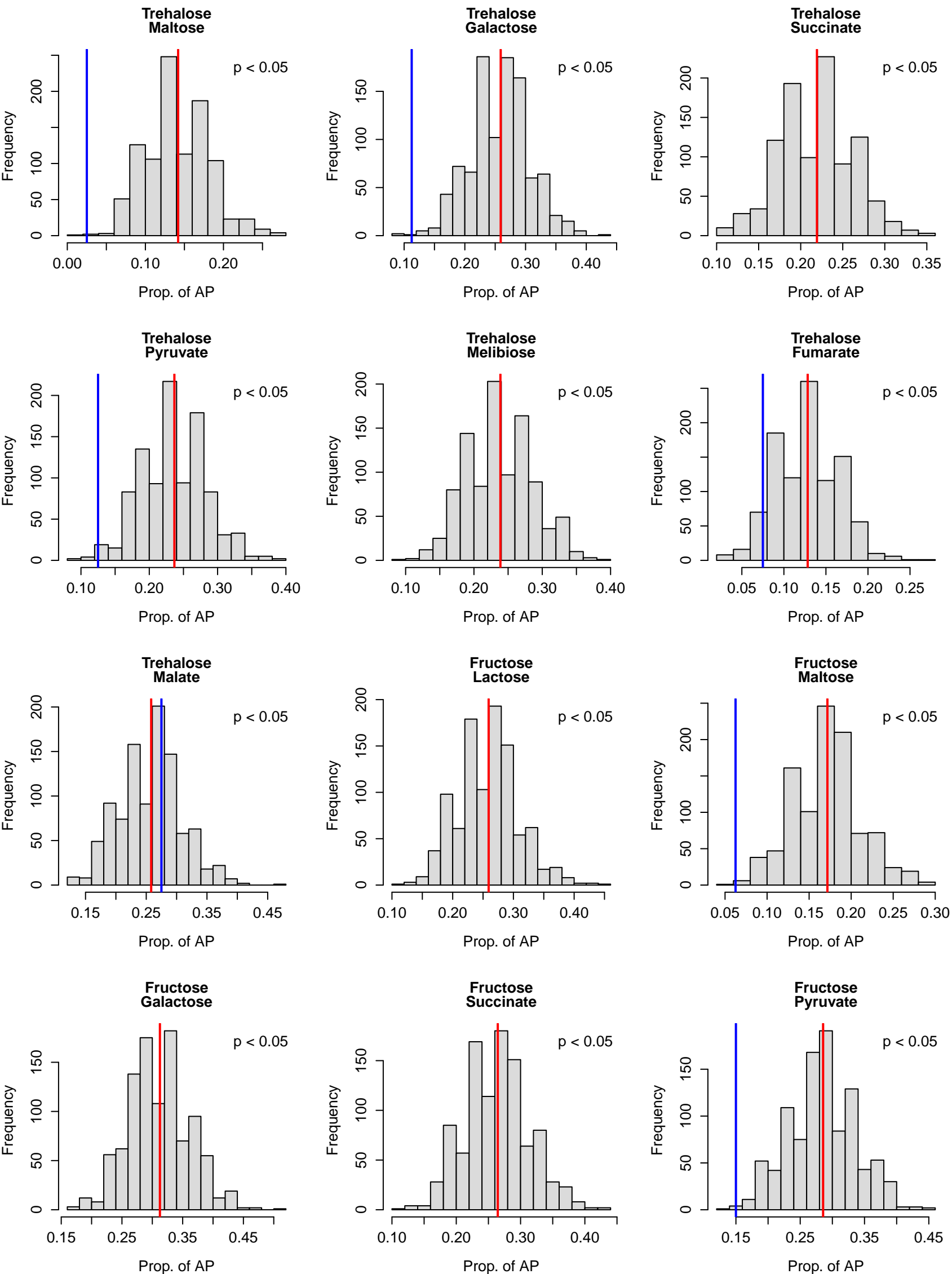

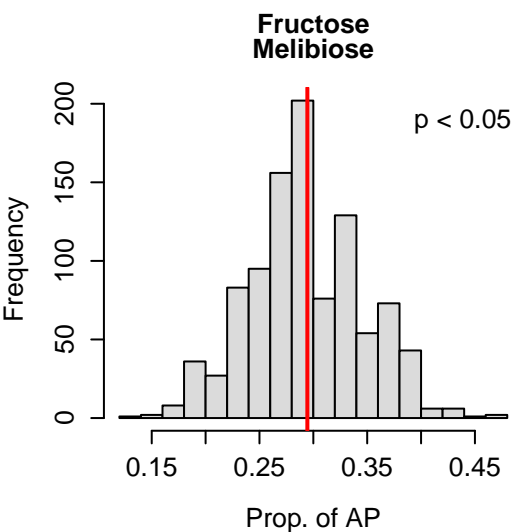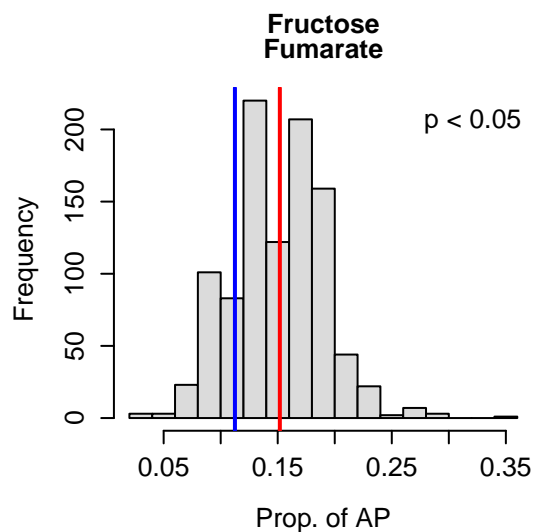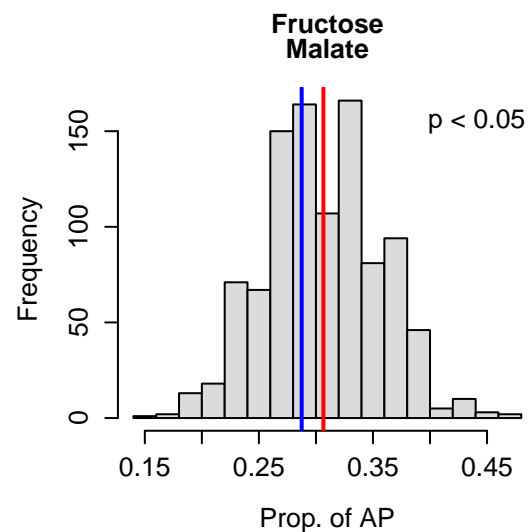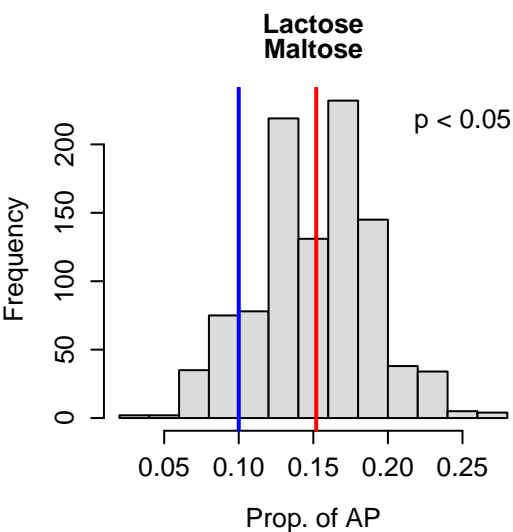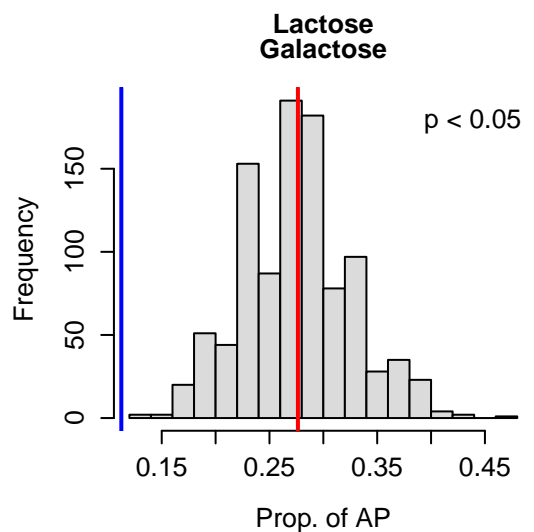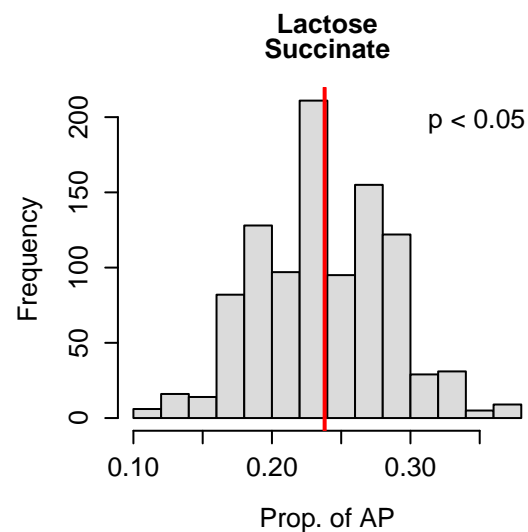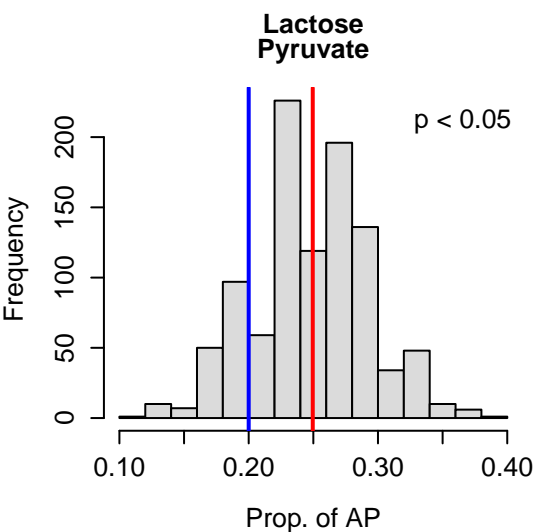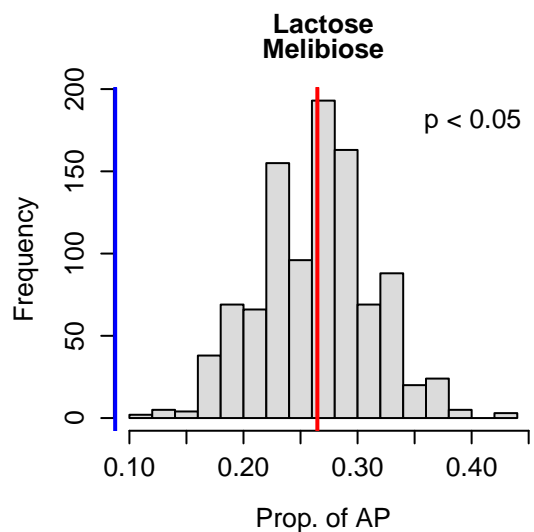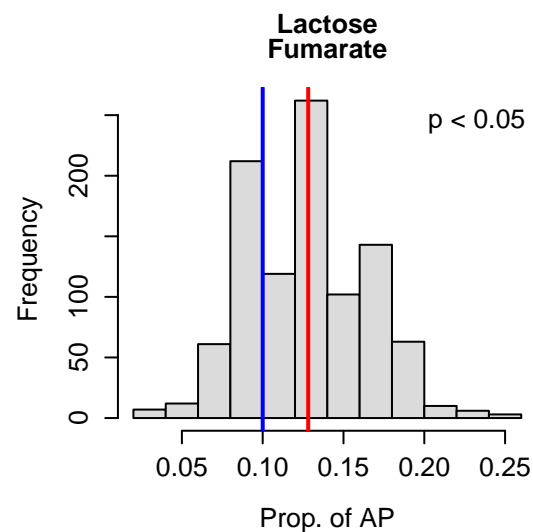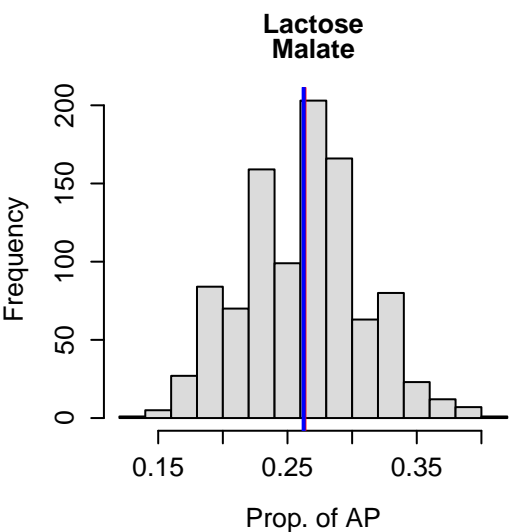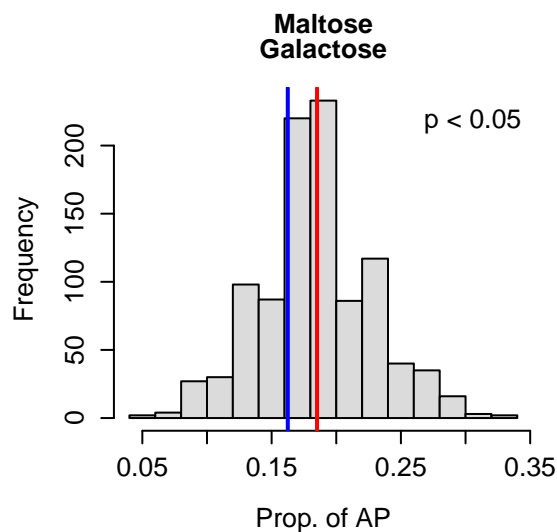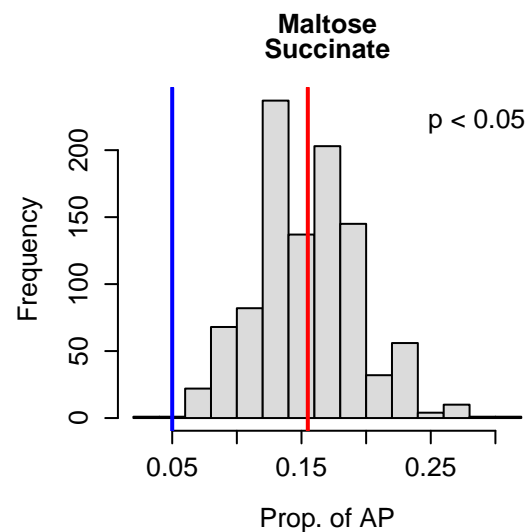

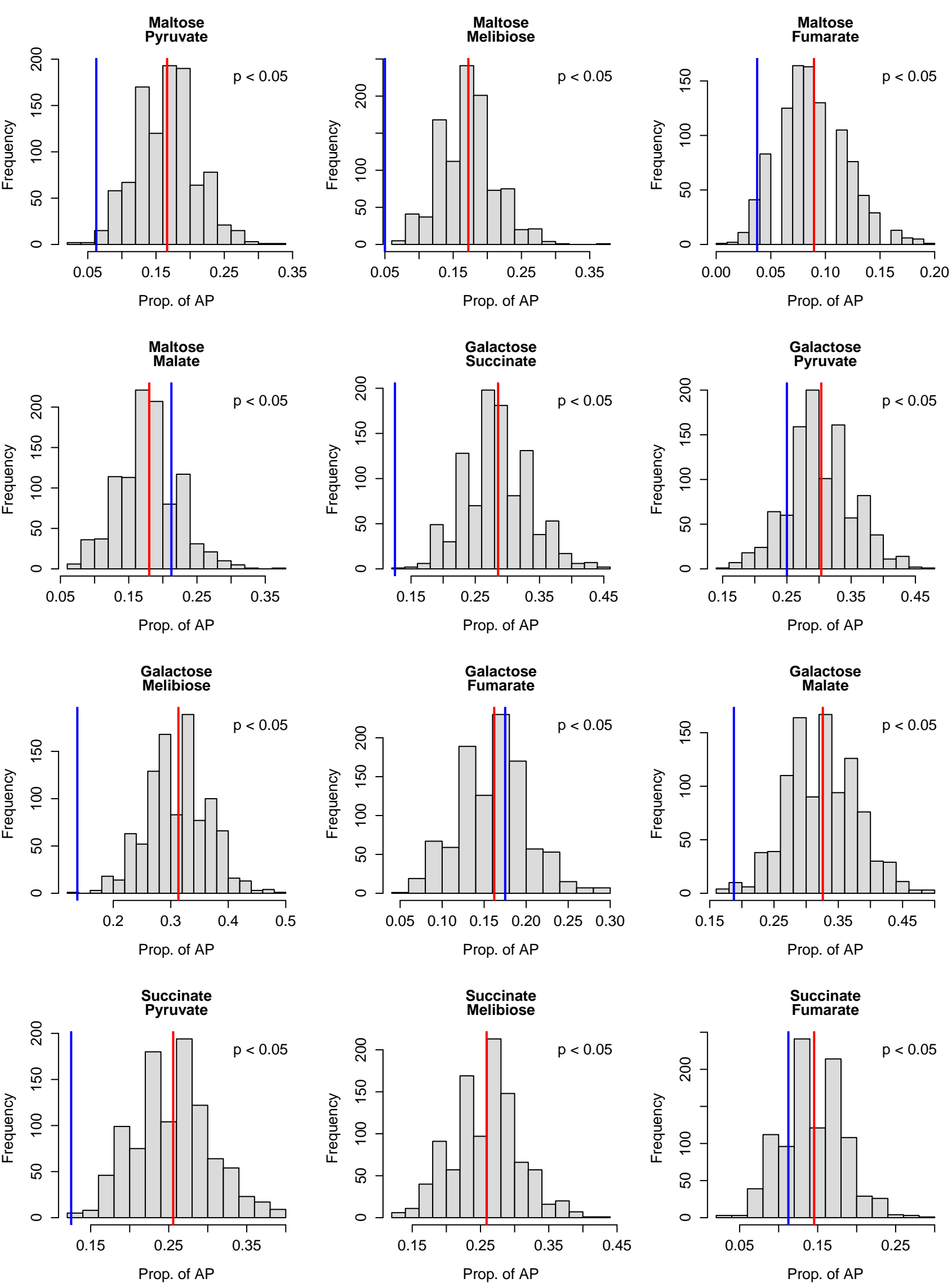

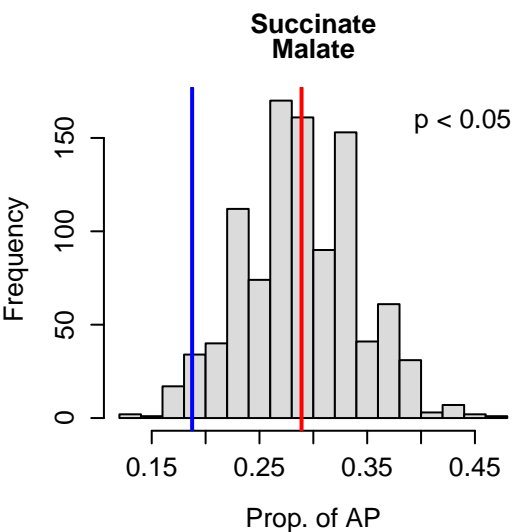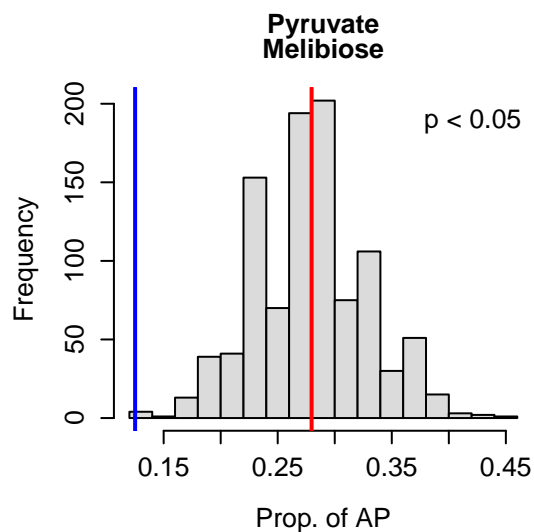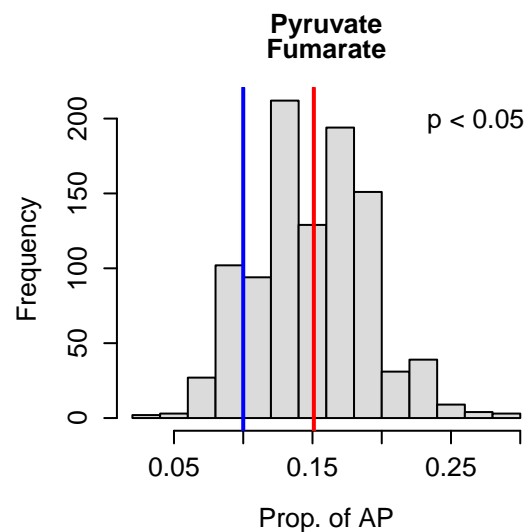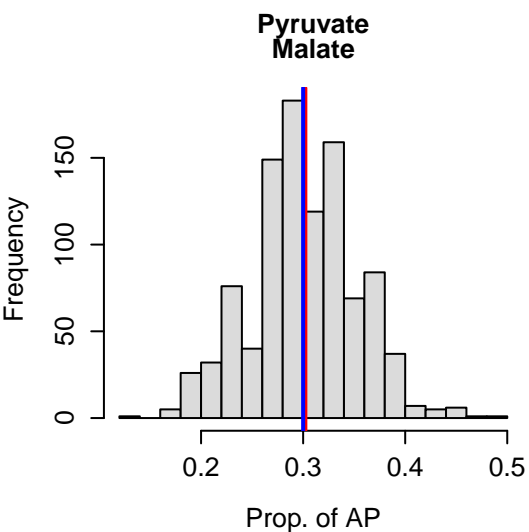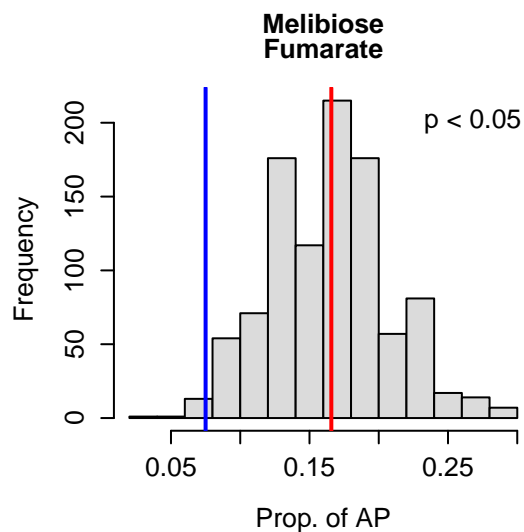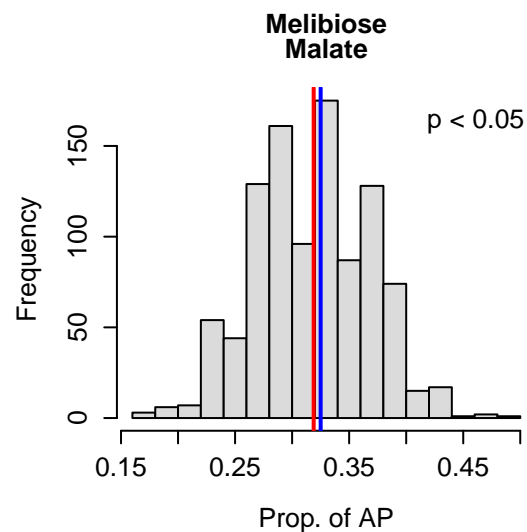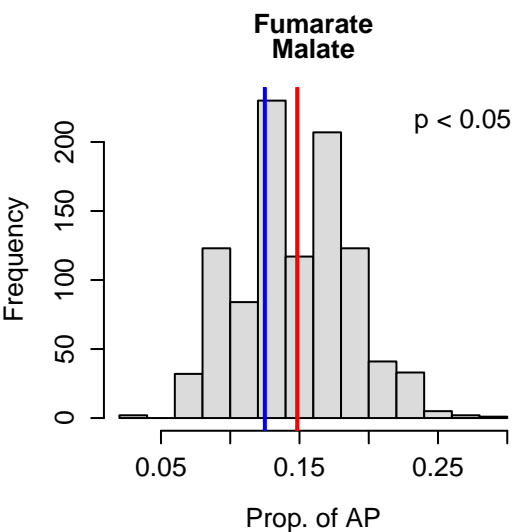

Supplement: Supplementary file 5 [file EVO-72-2202-s005.pdf]

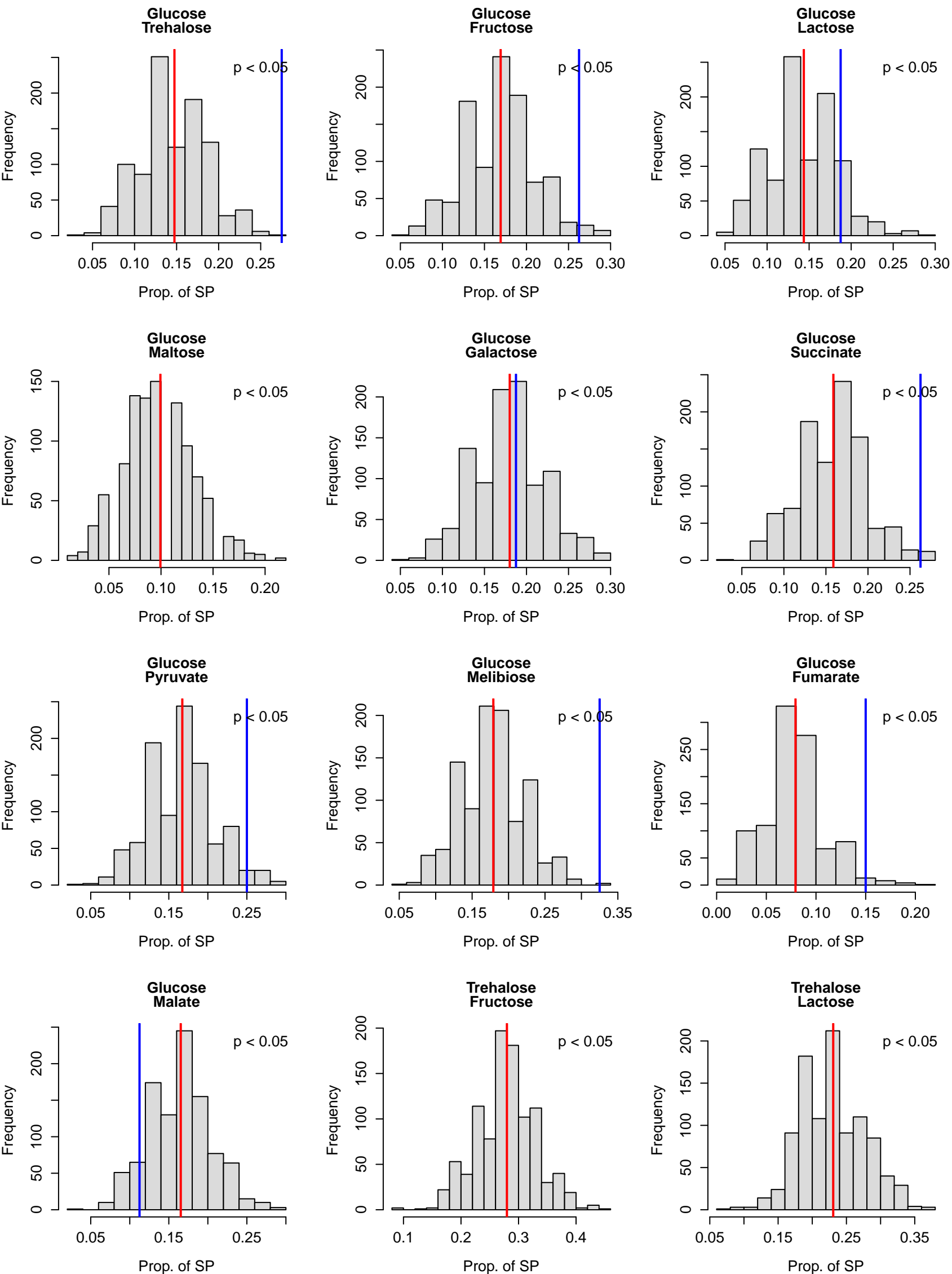

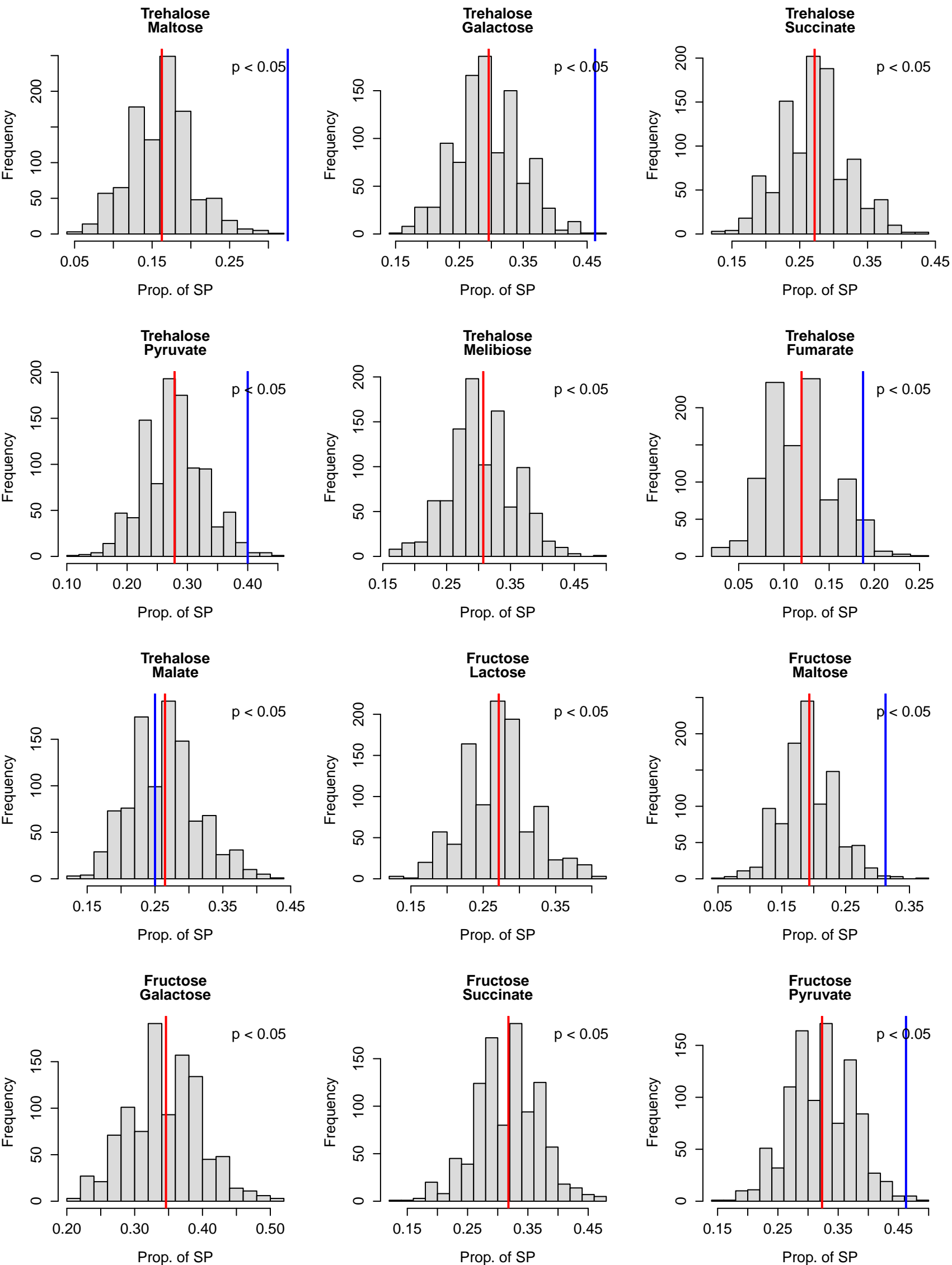

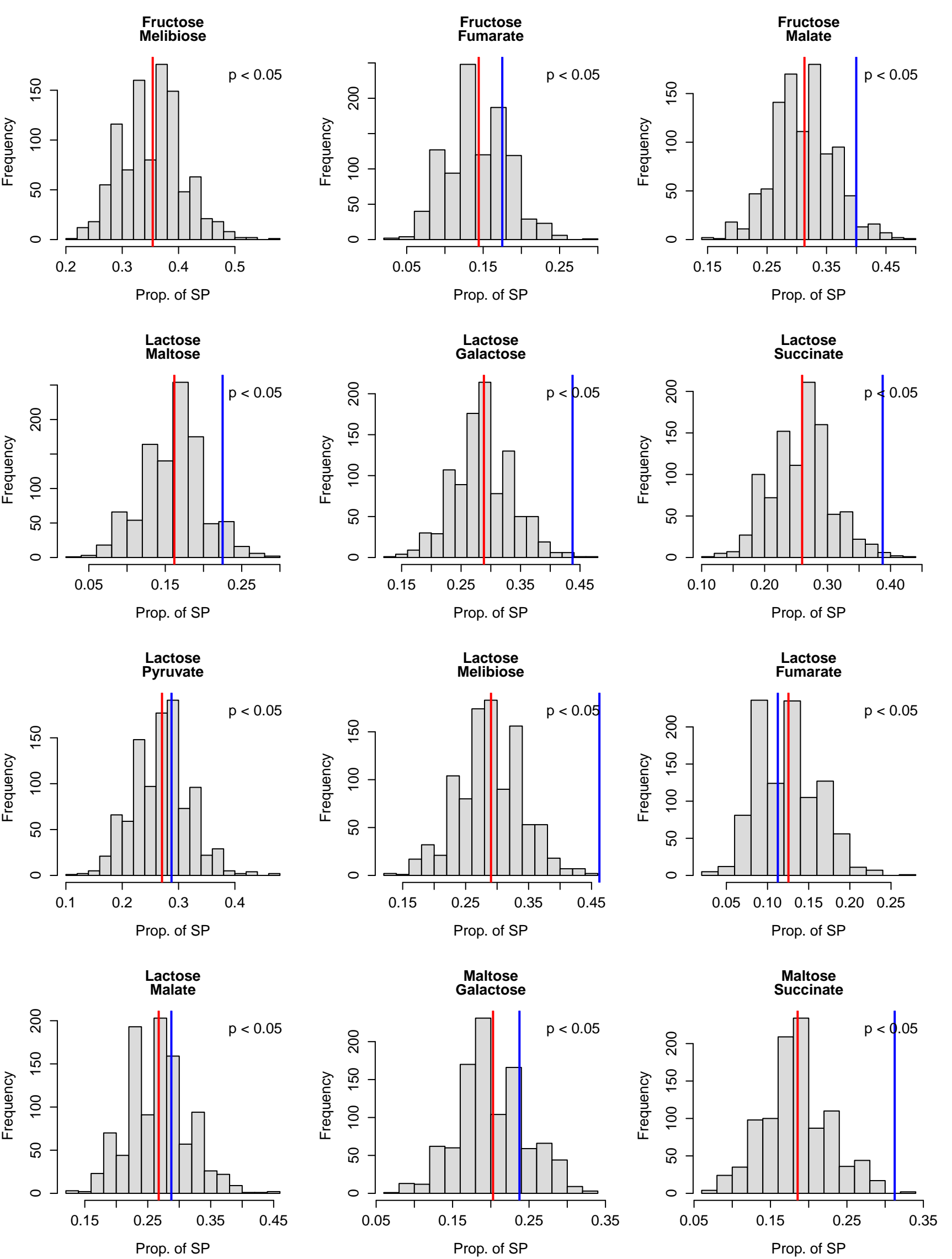

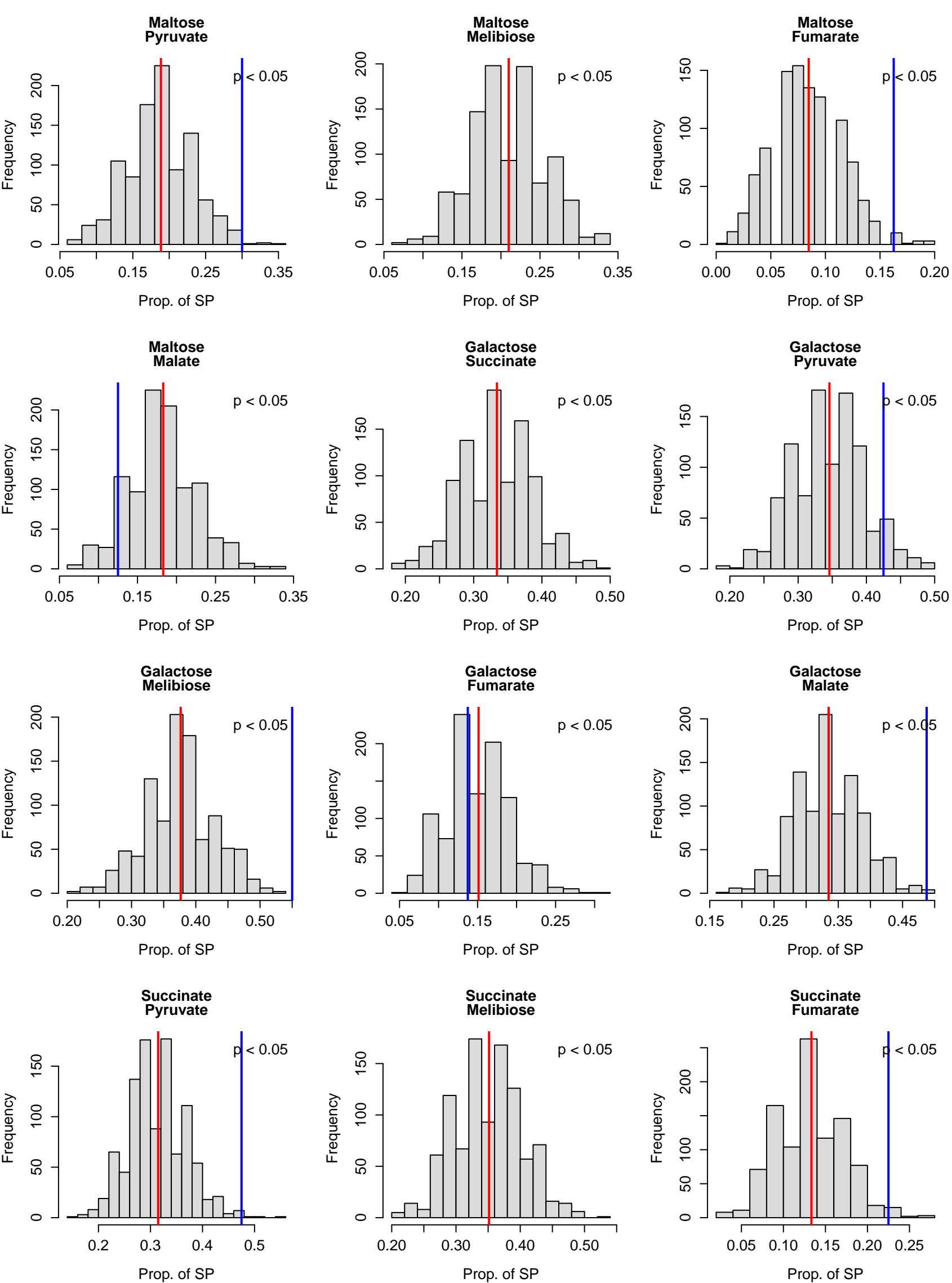

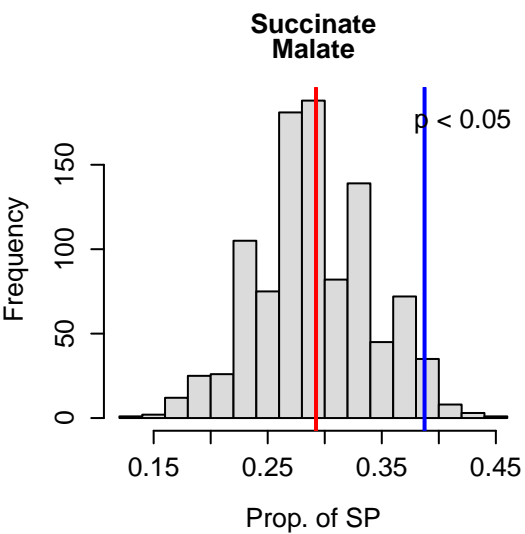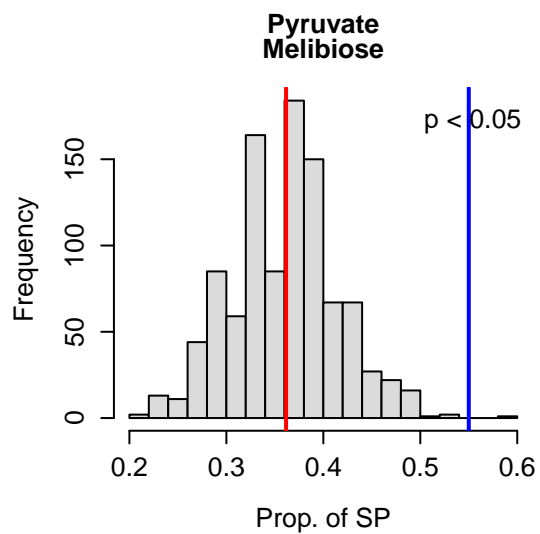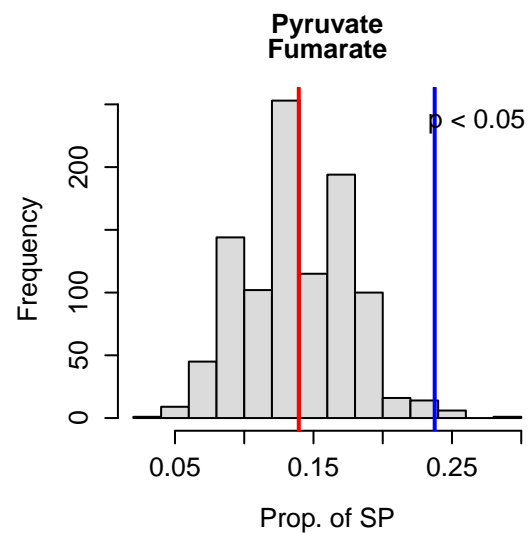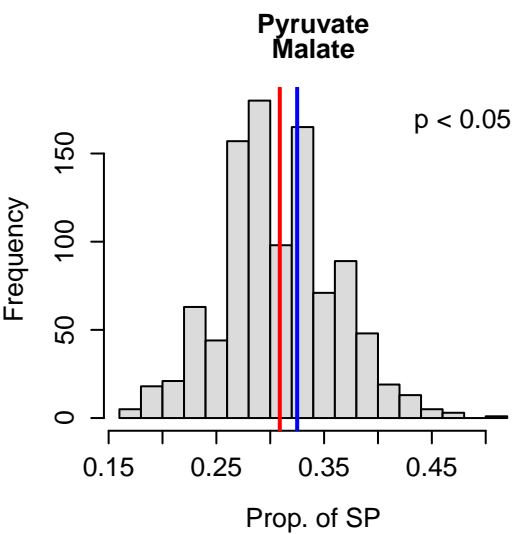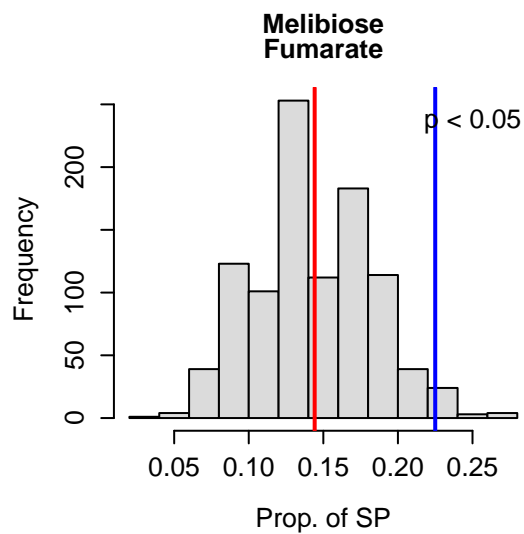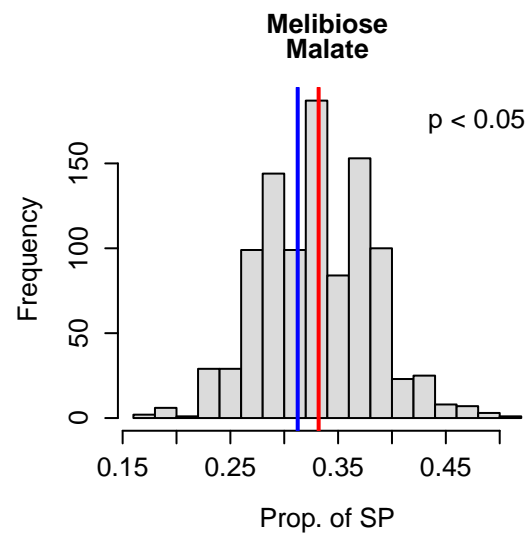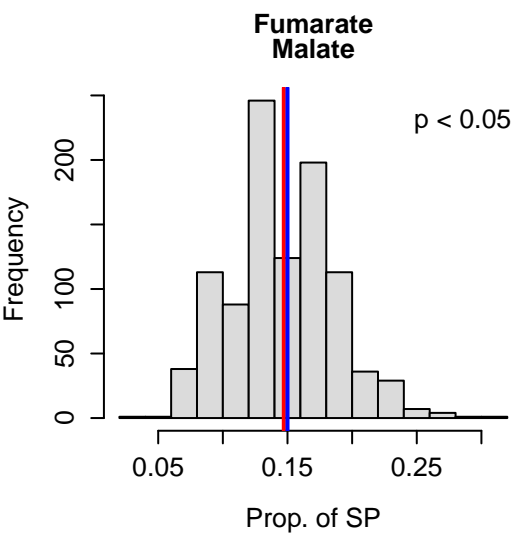

Supplement: Supplementary file 6 [file EVO-72-2202-s006.pdf]

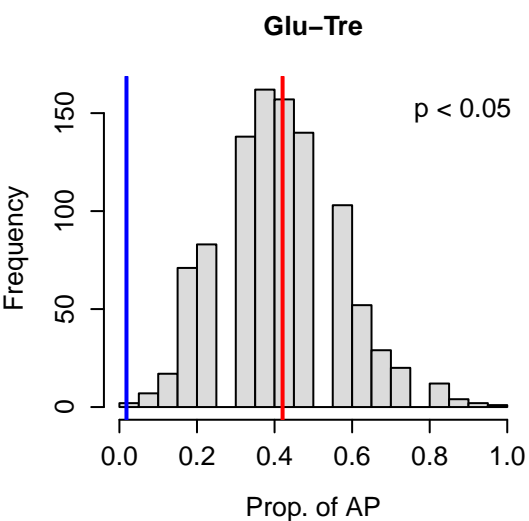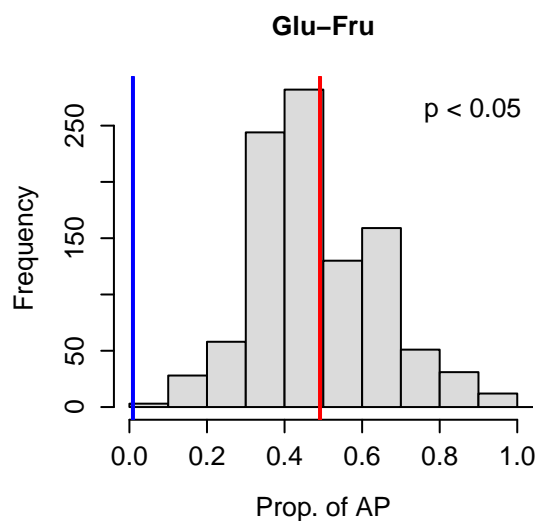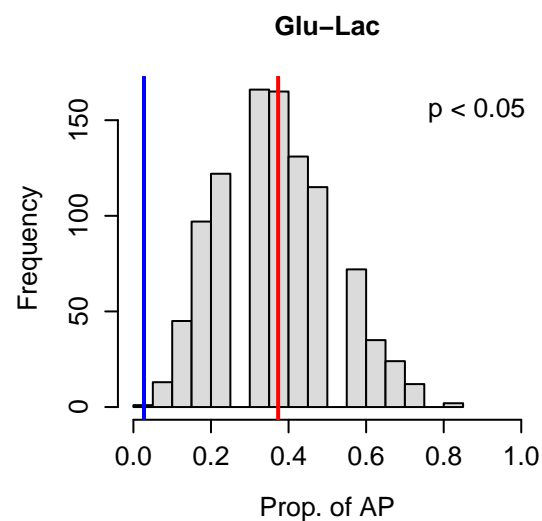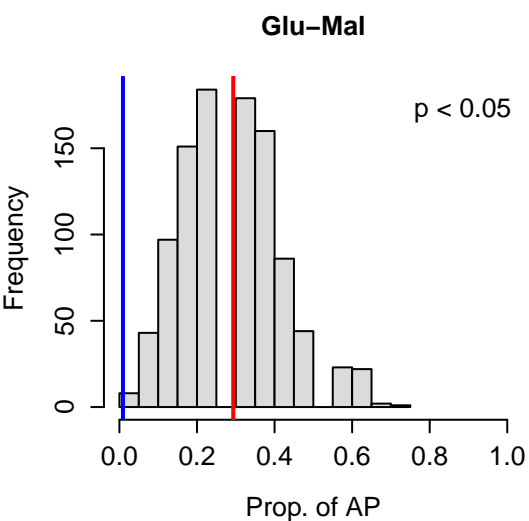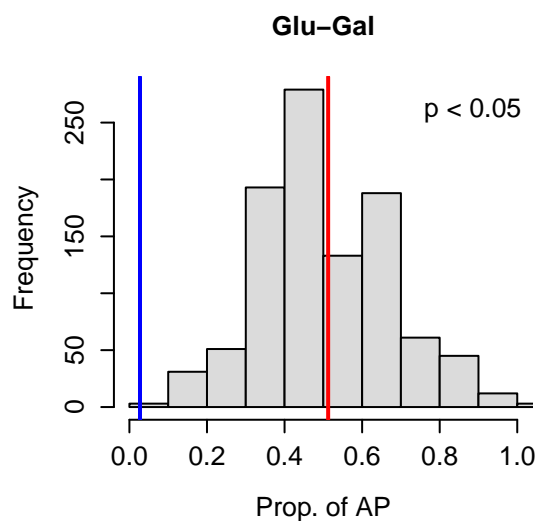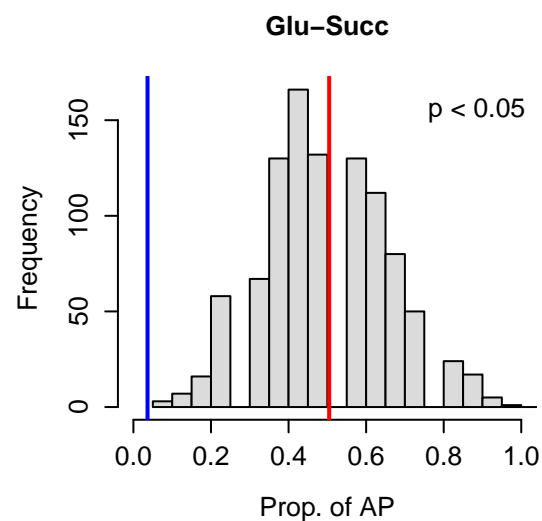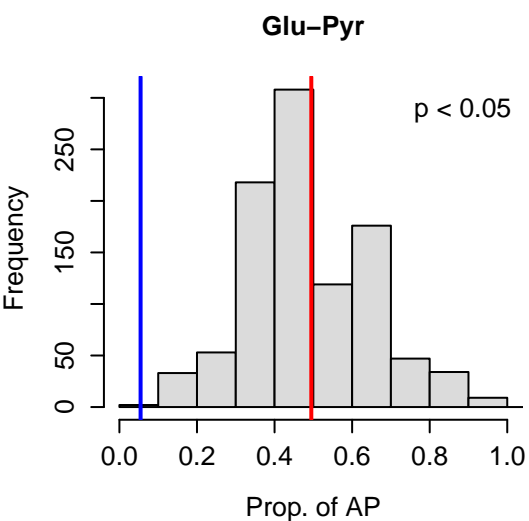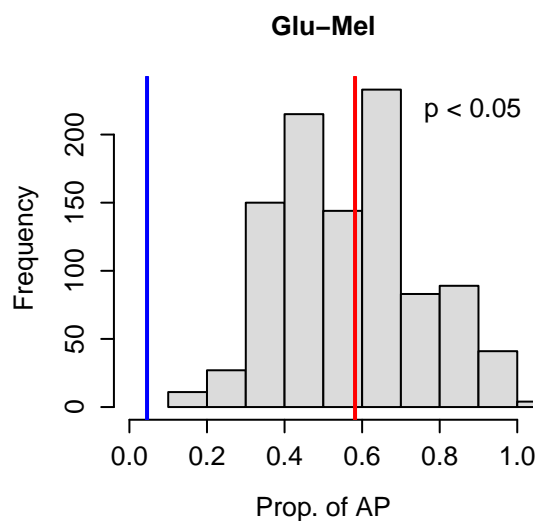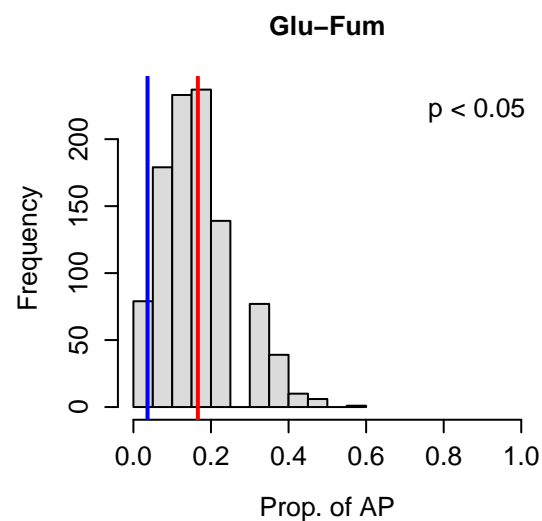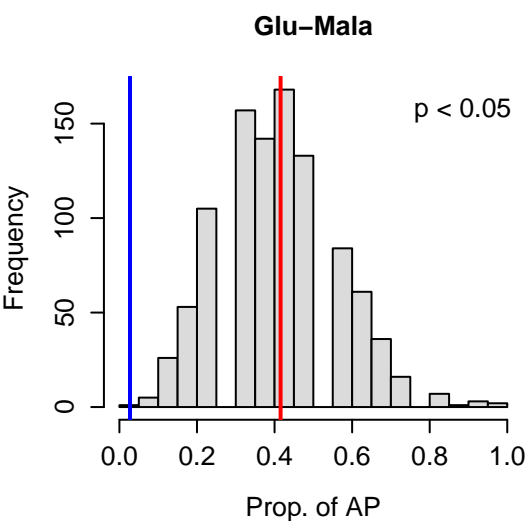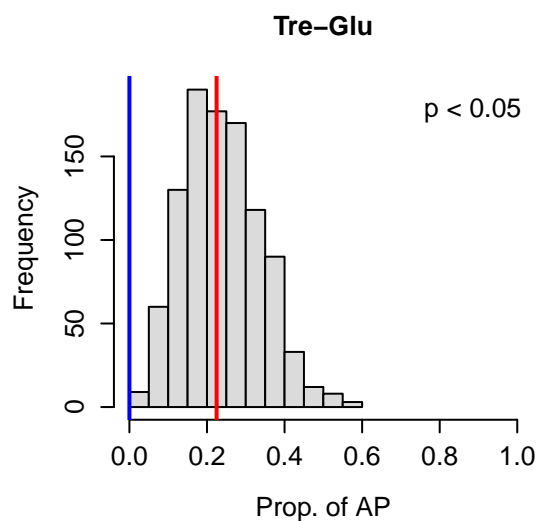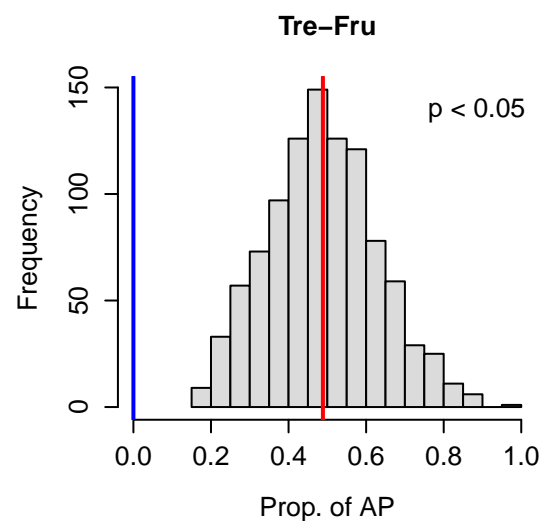

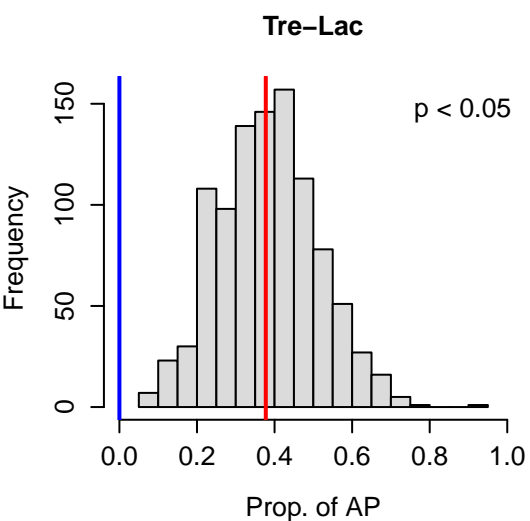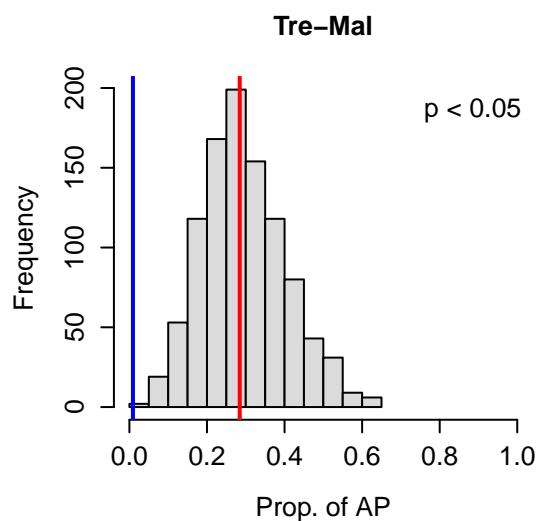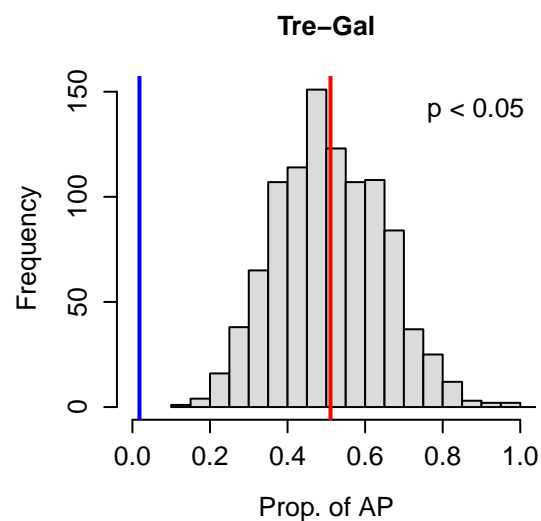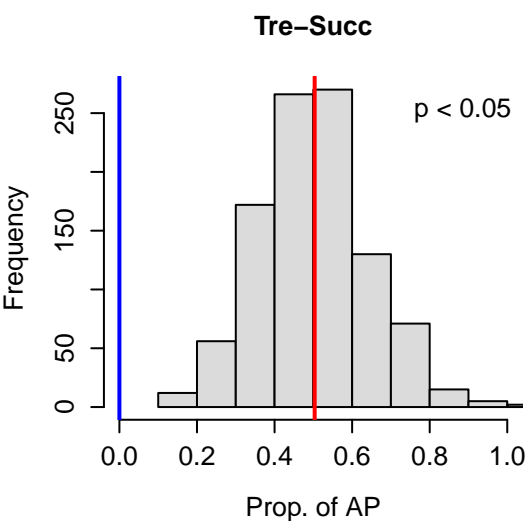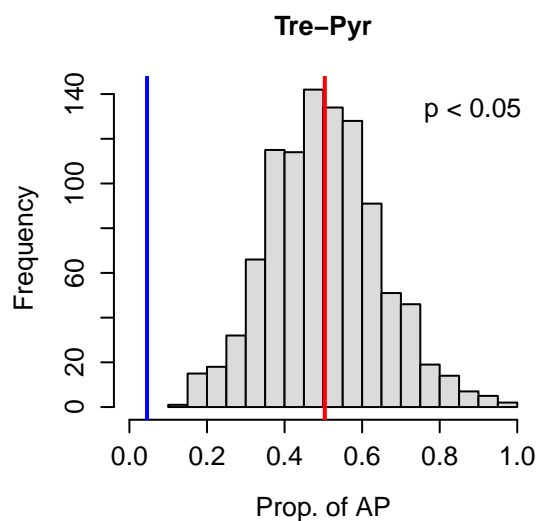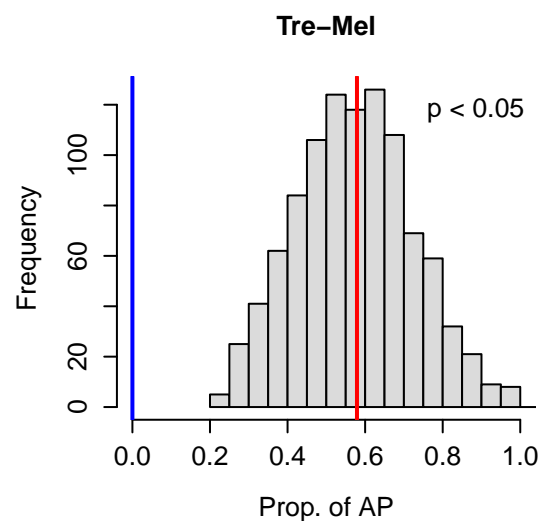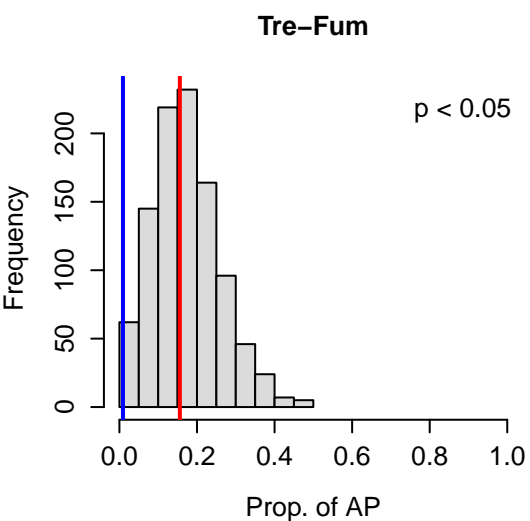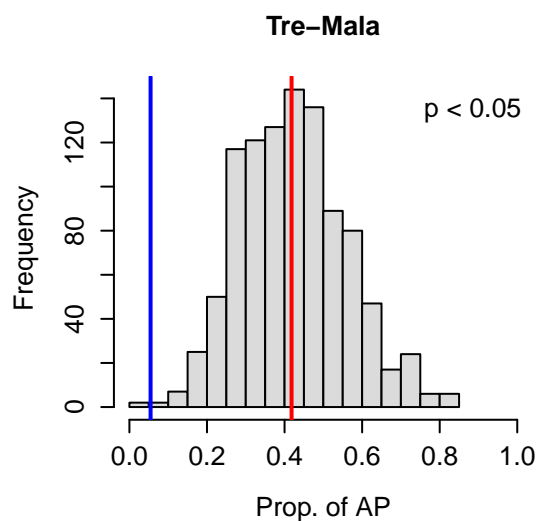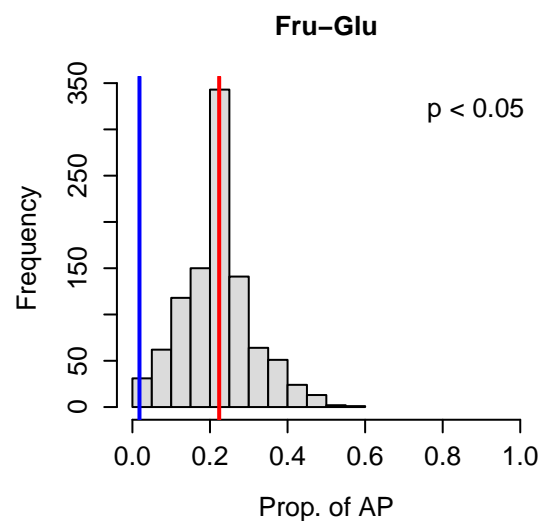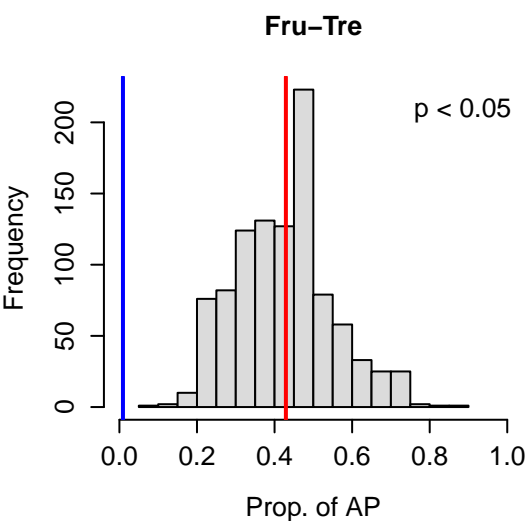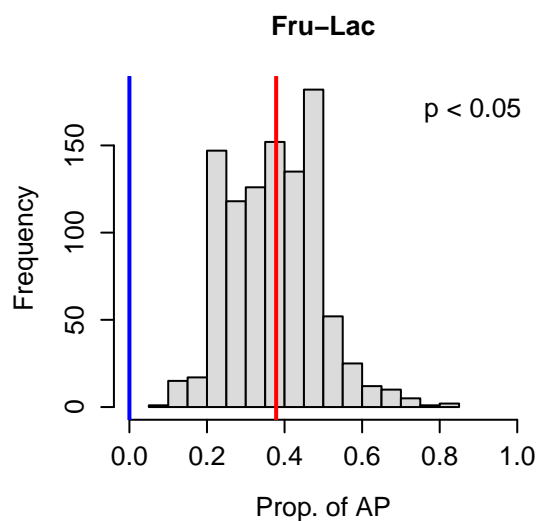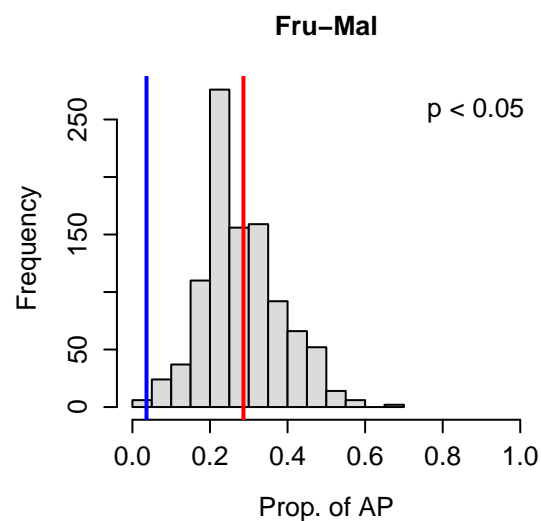

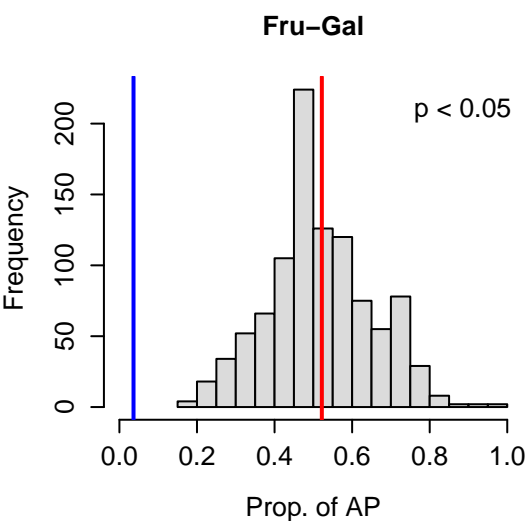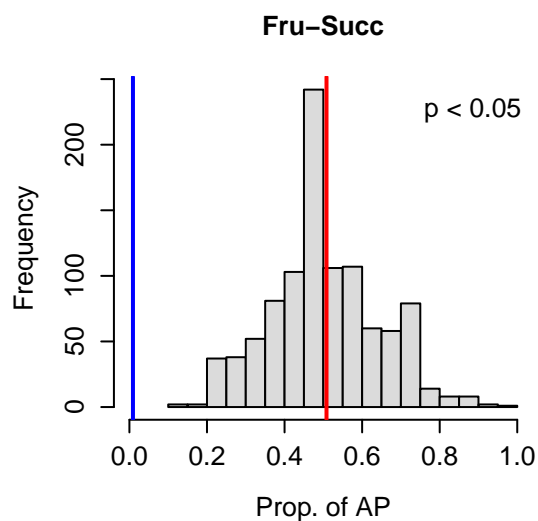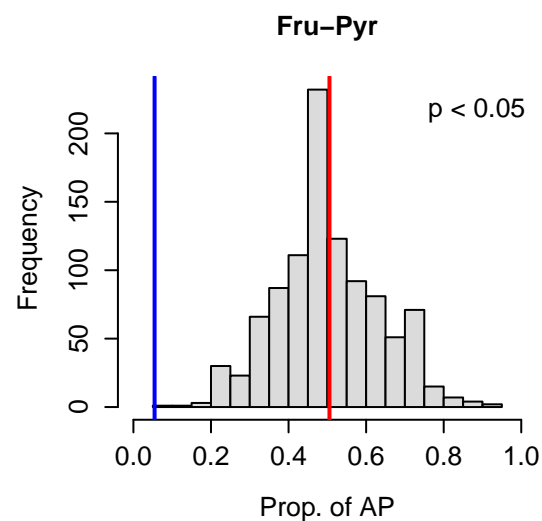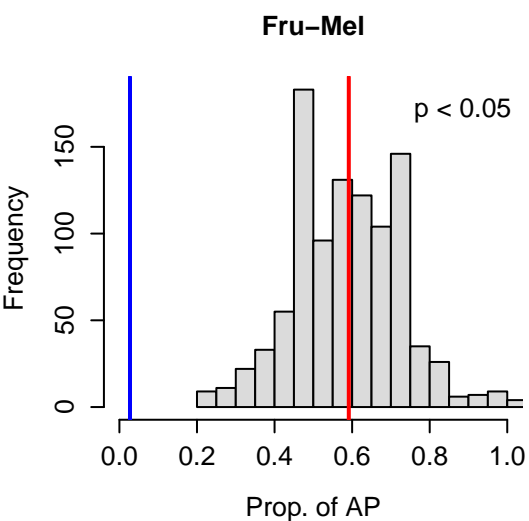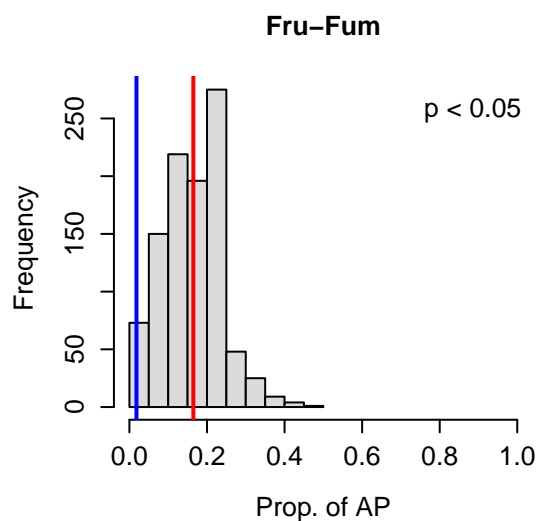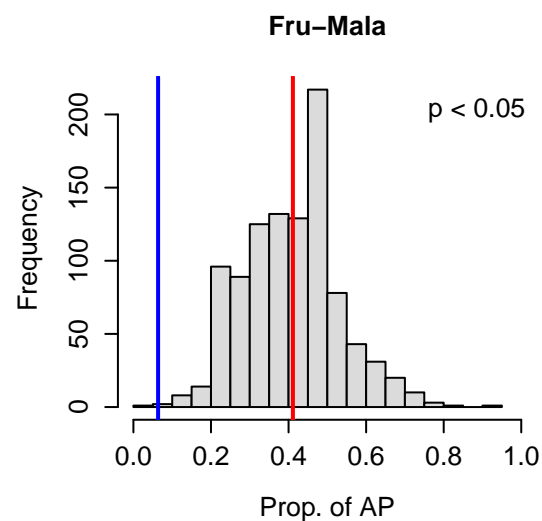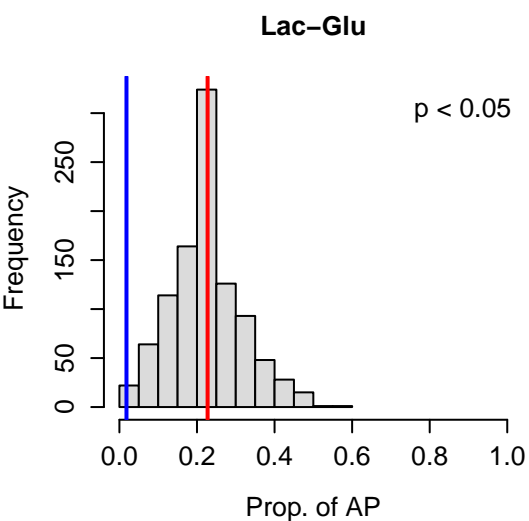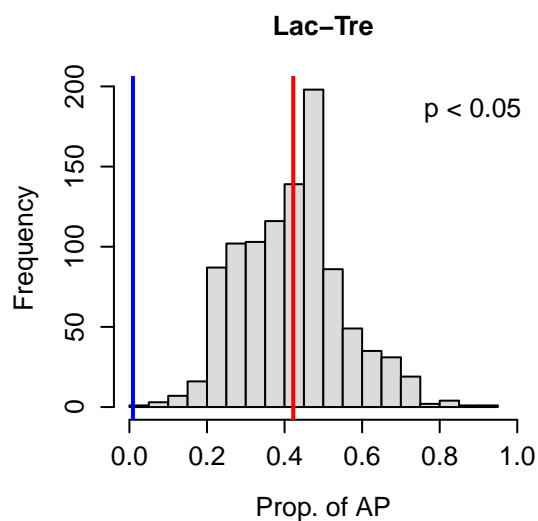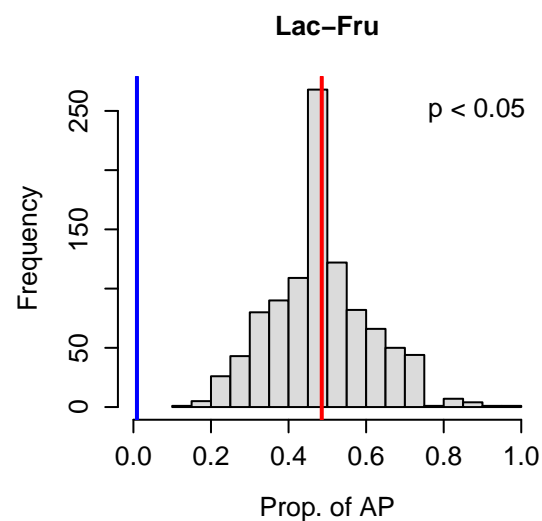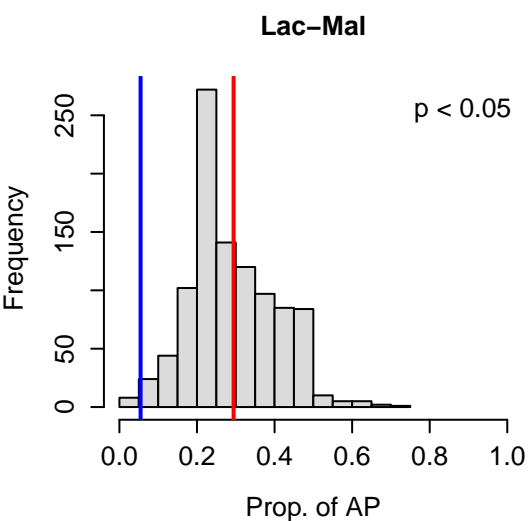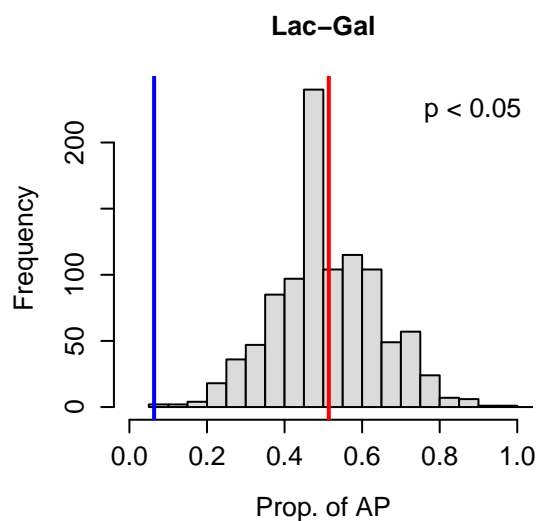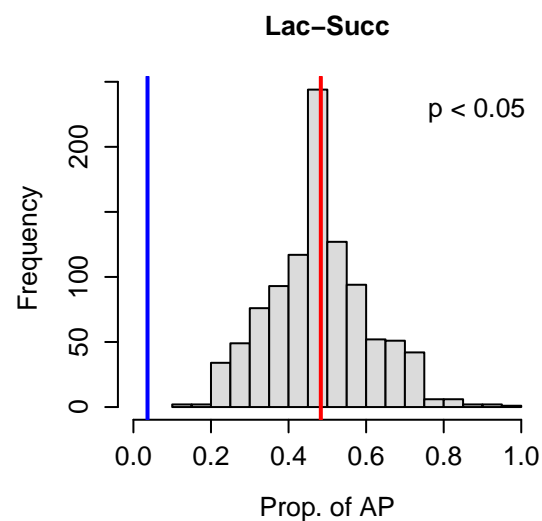

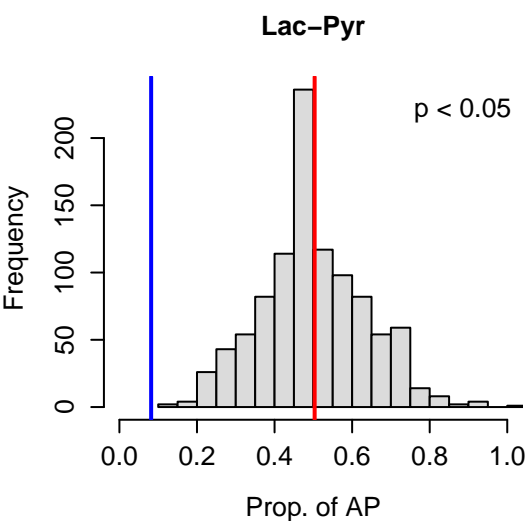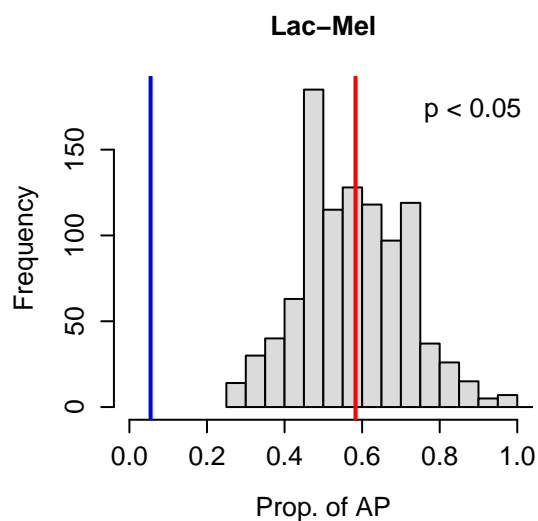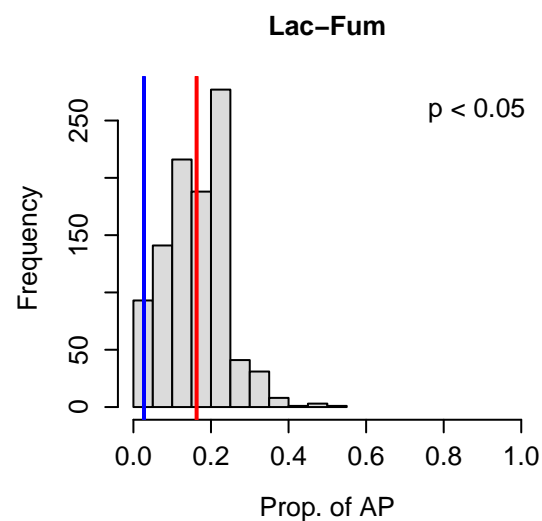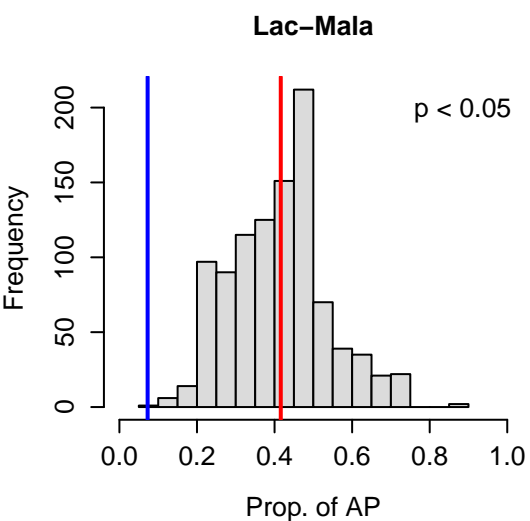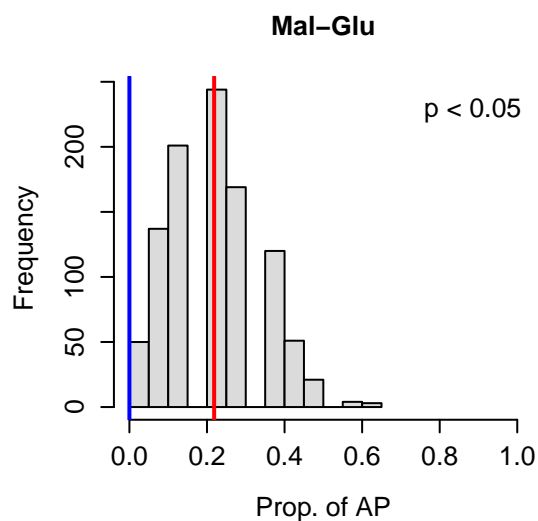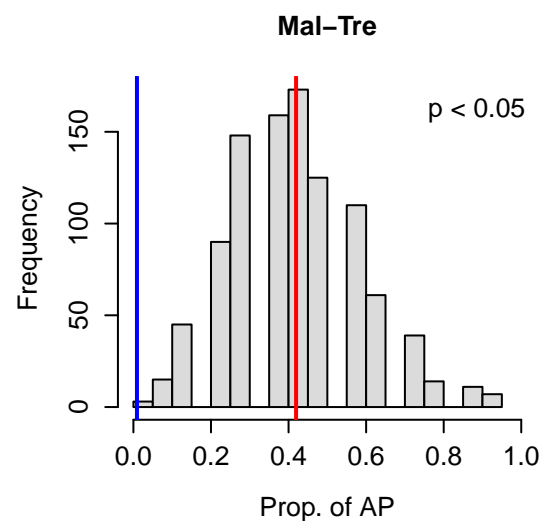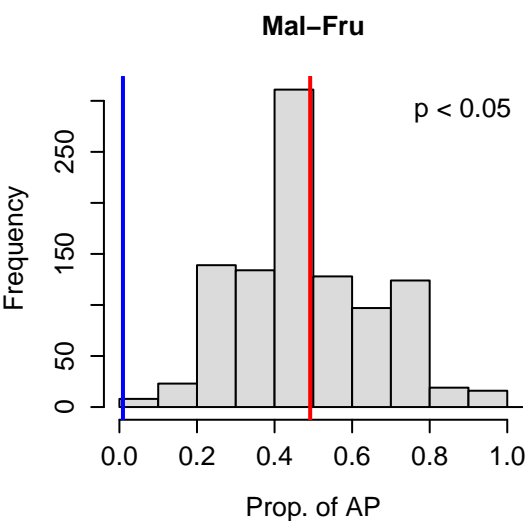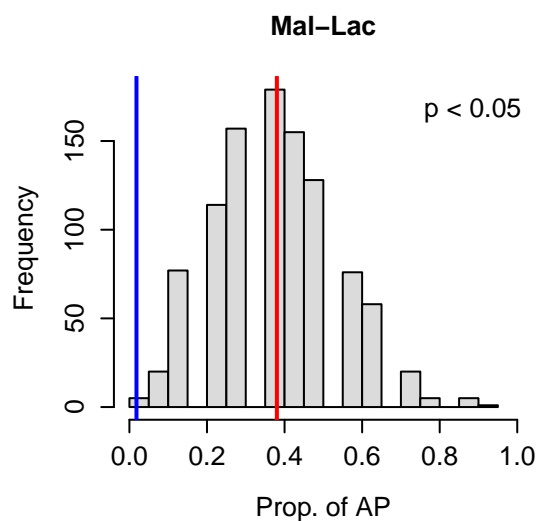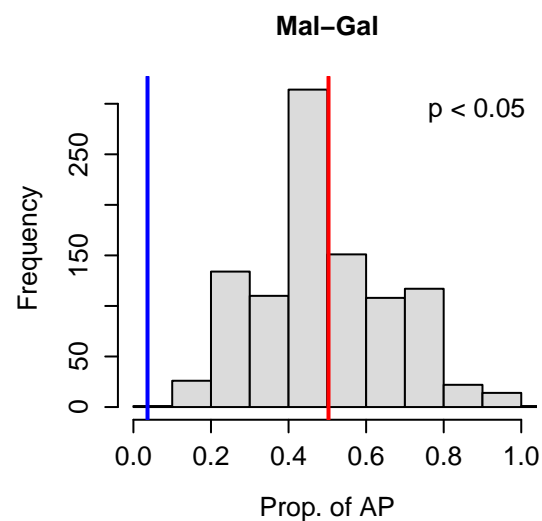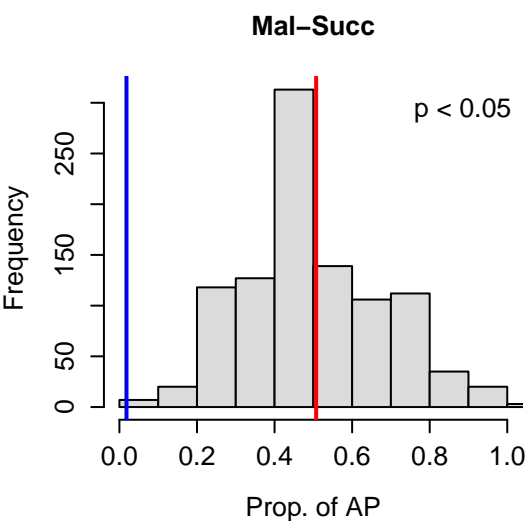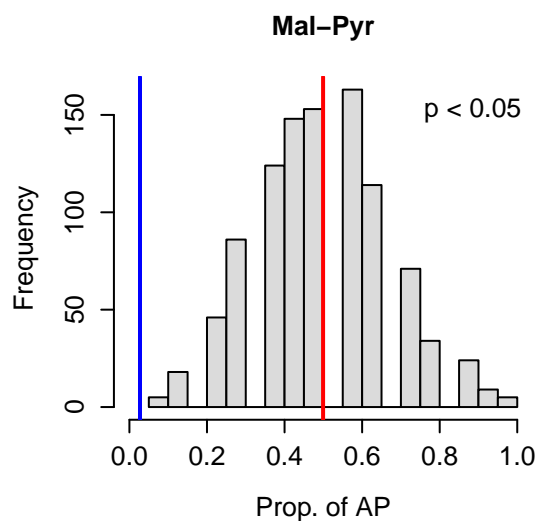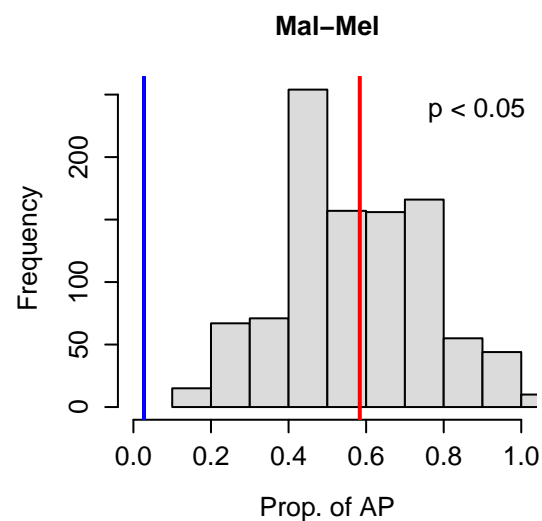

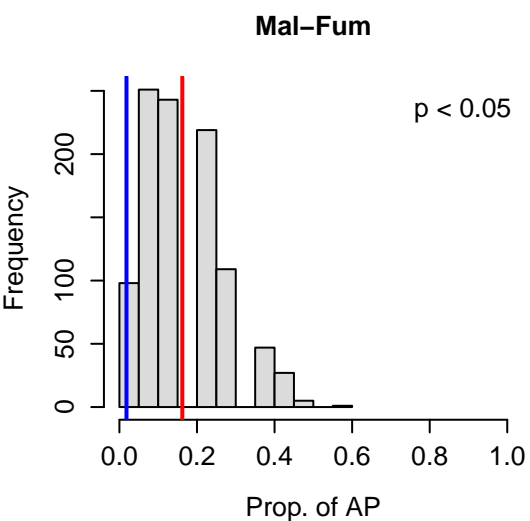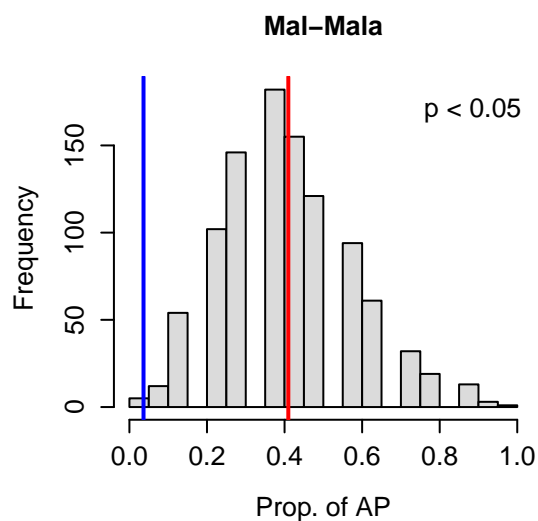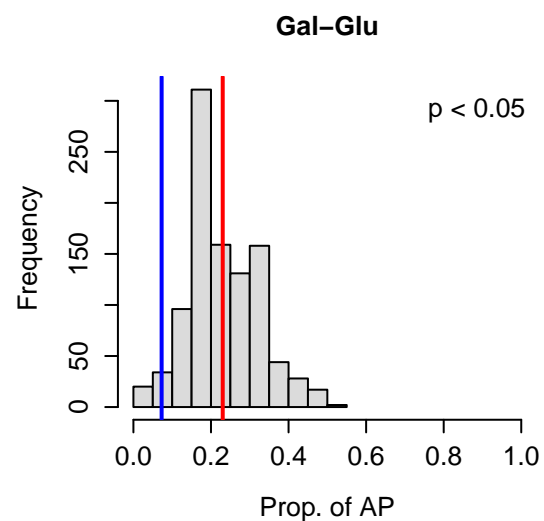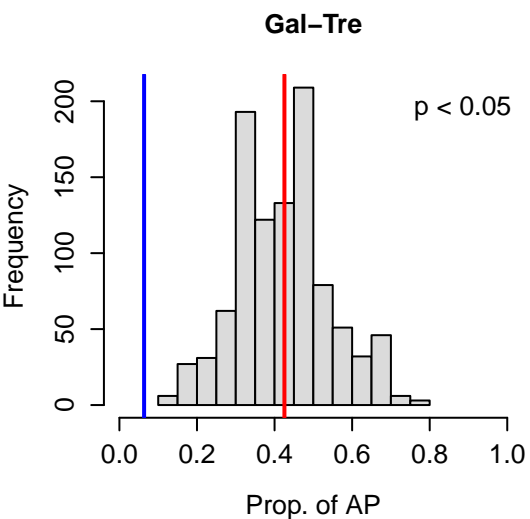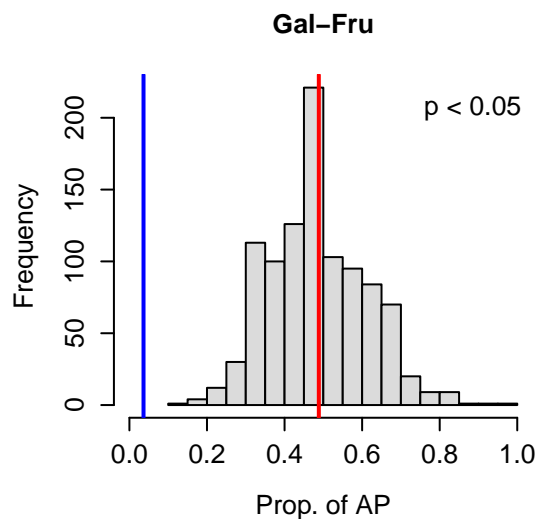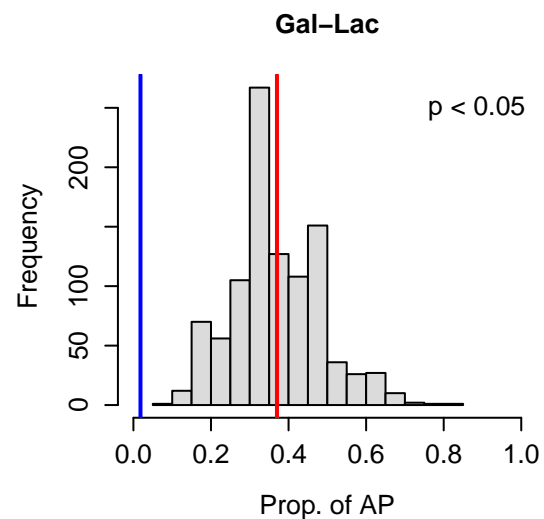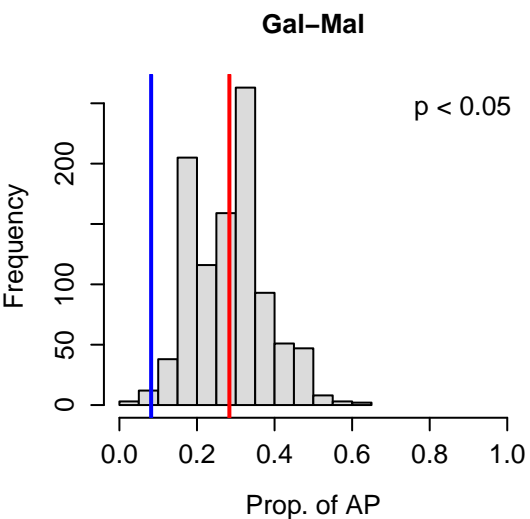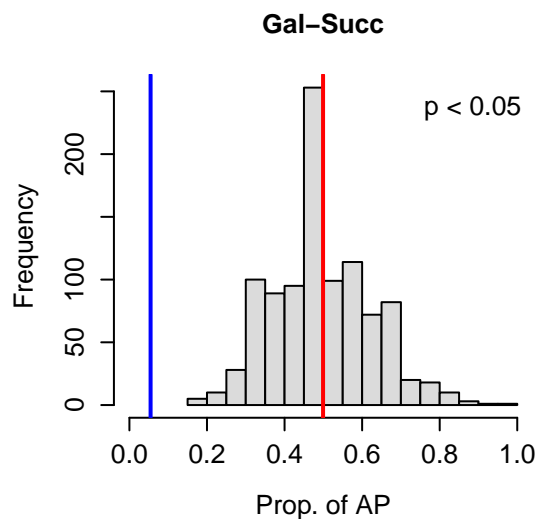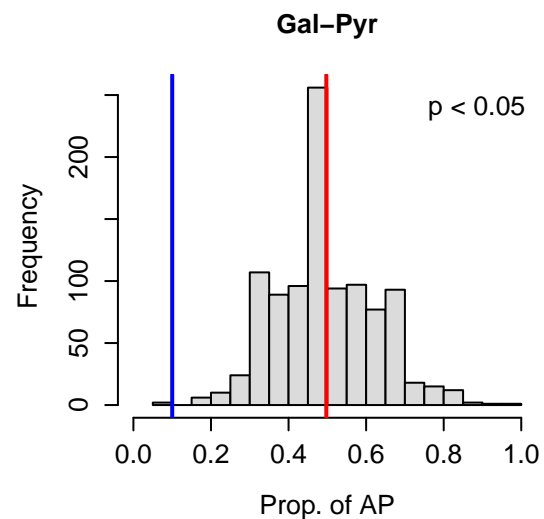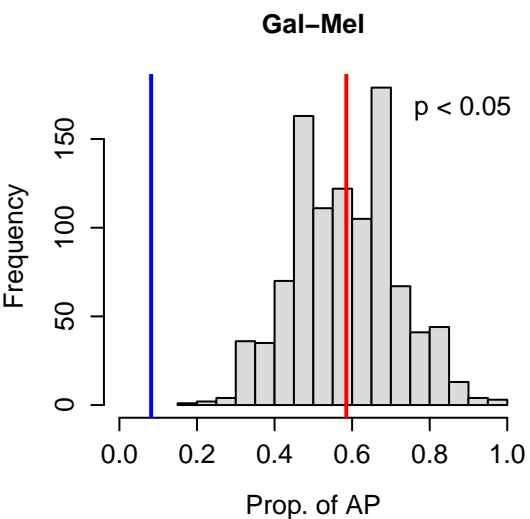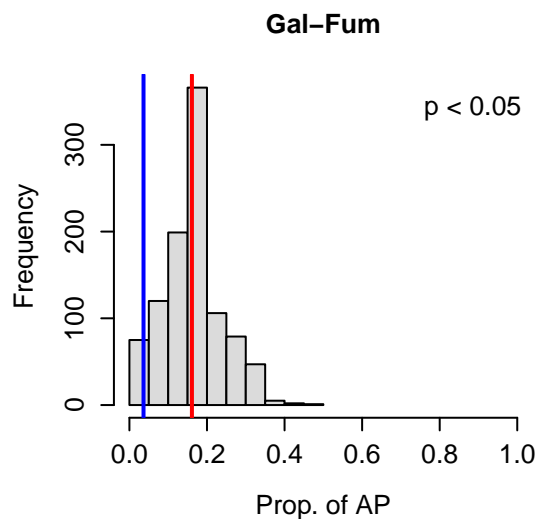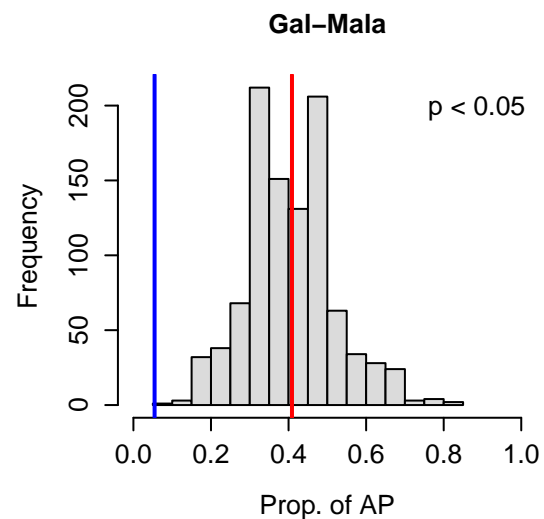

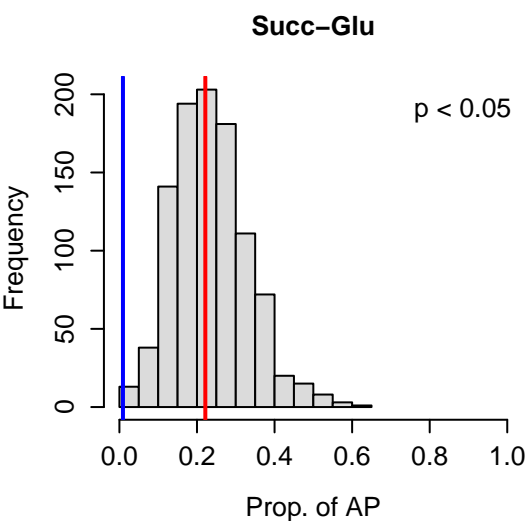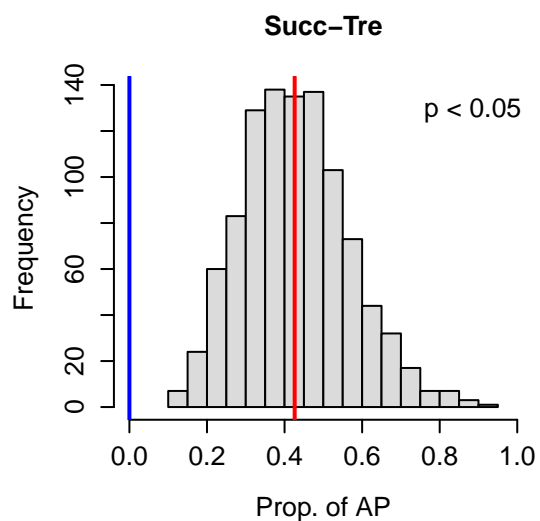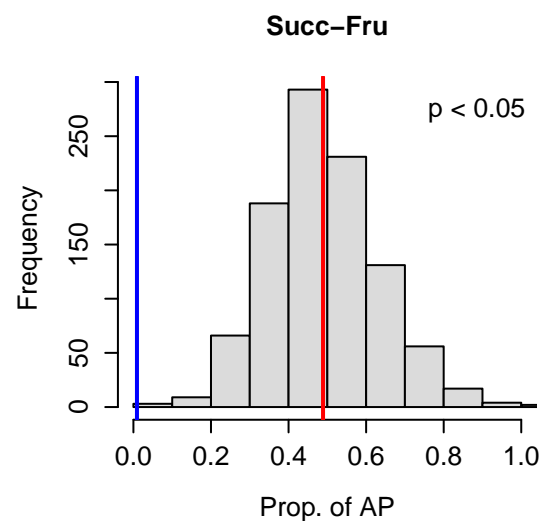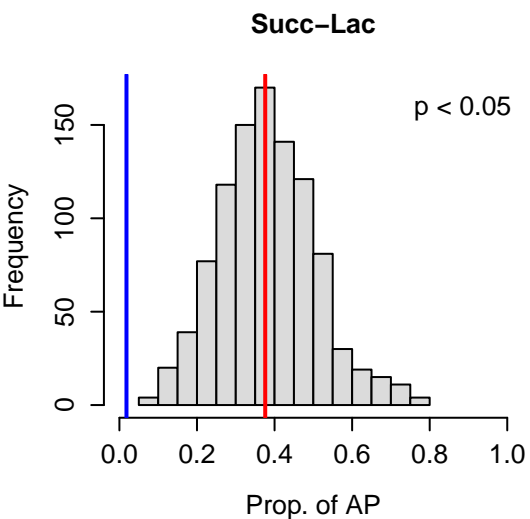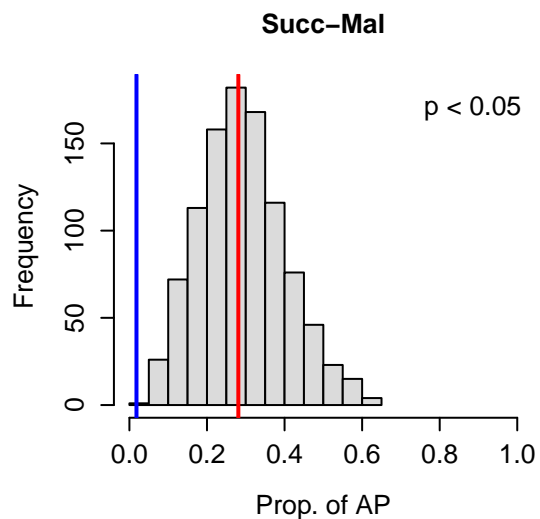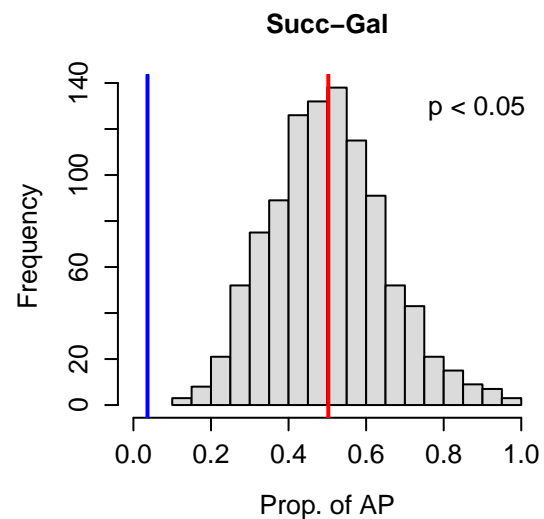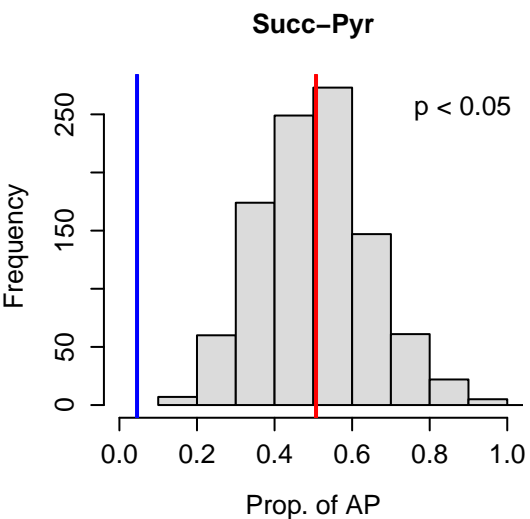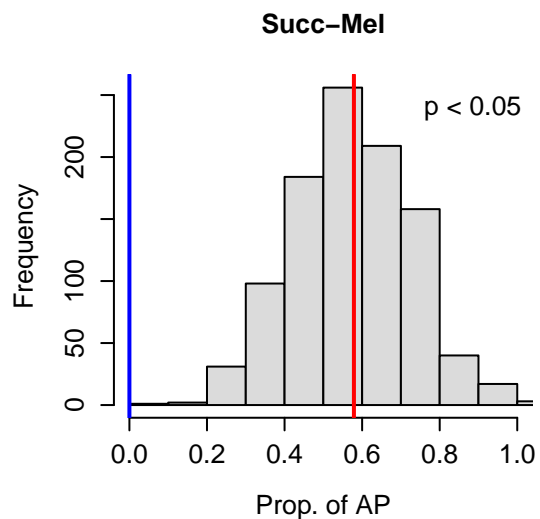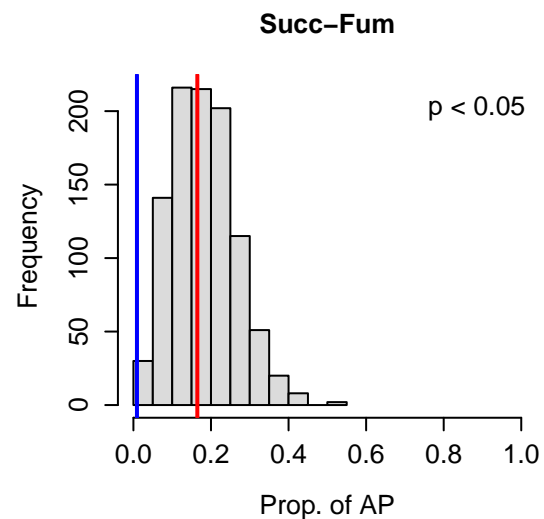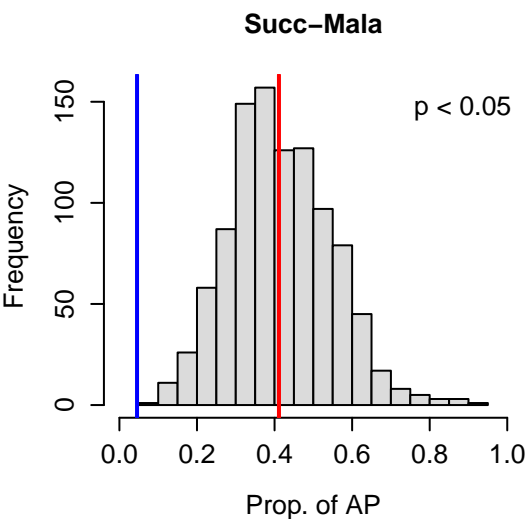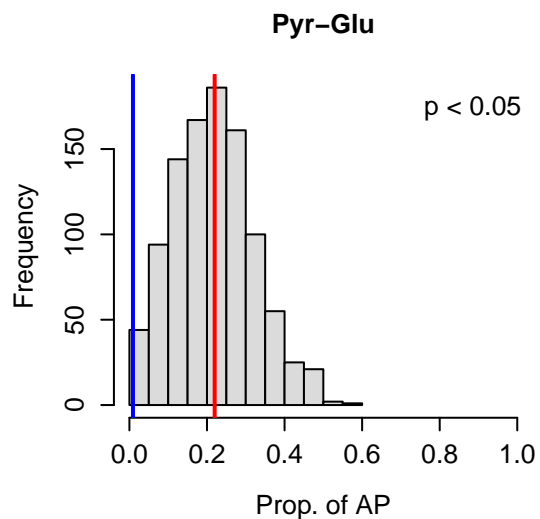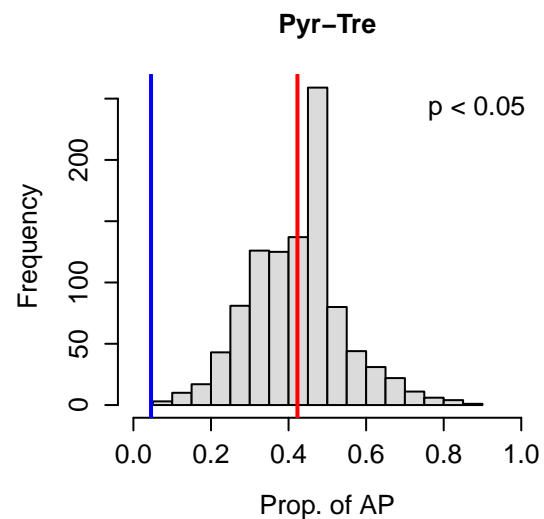

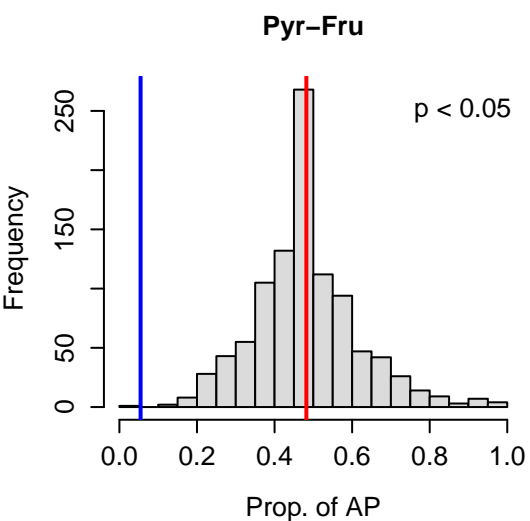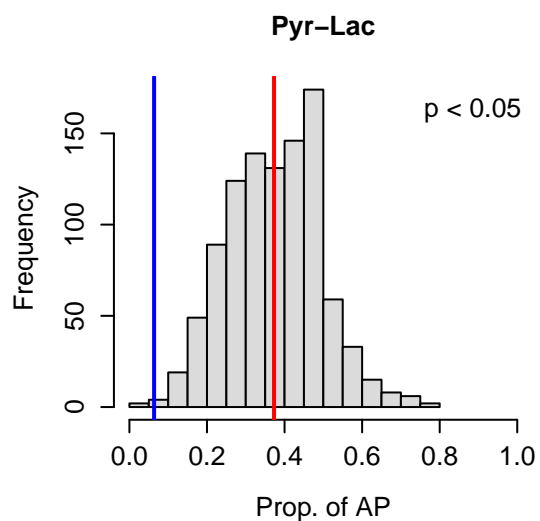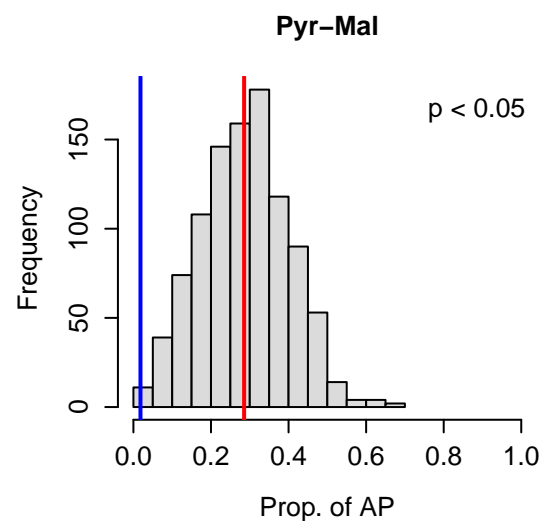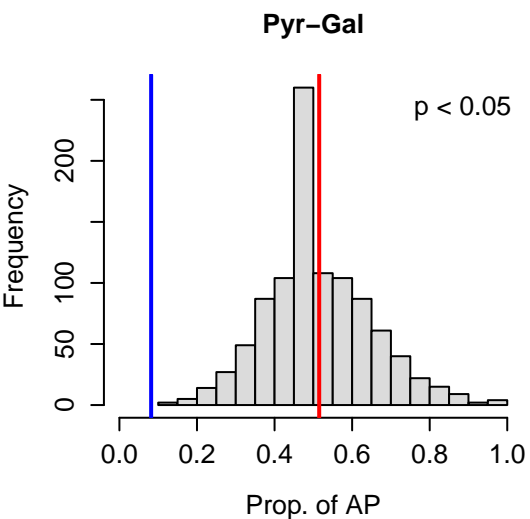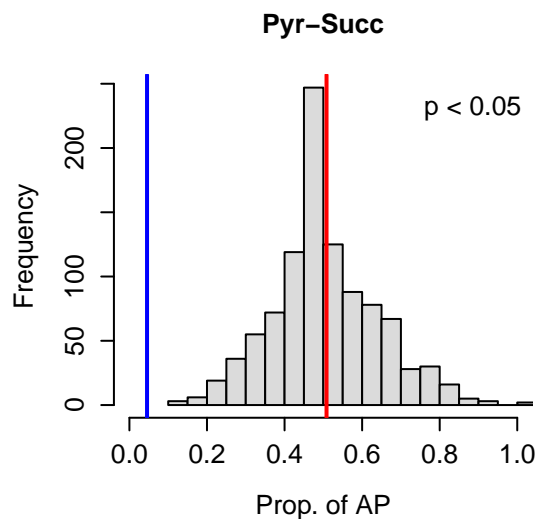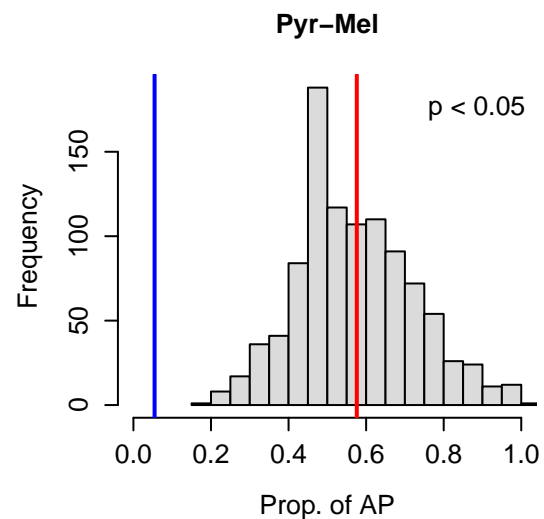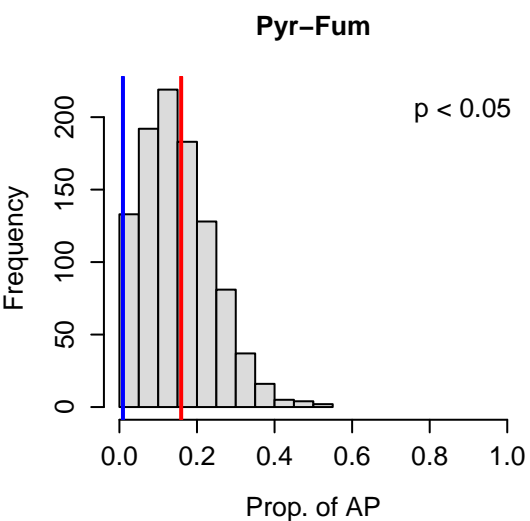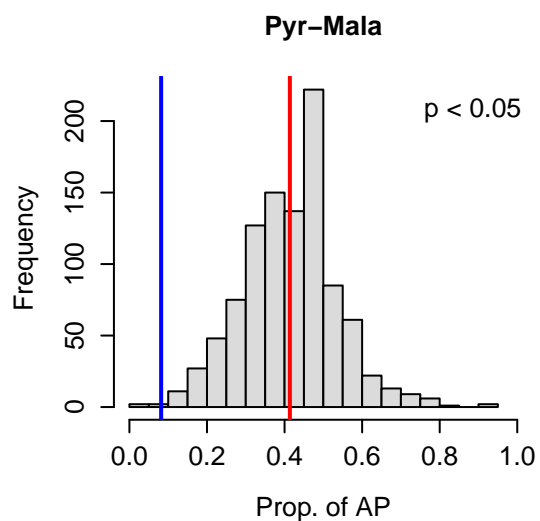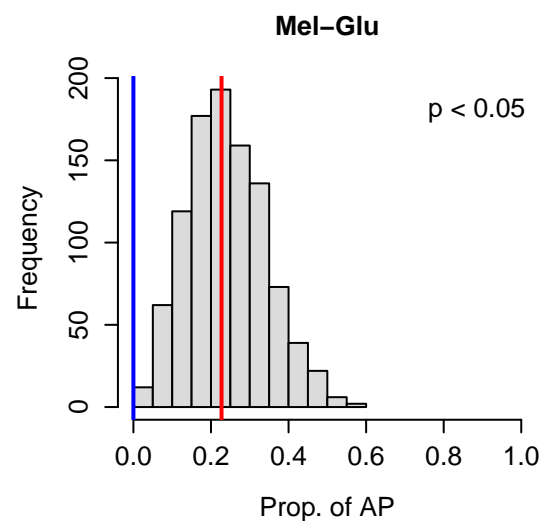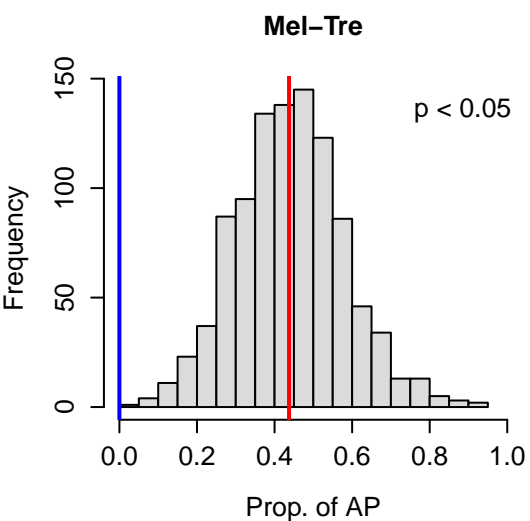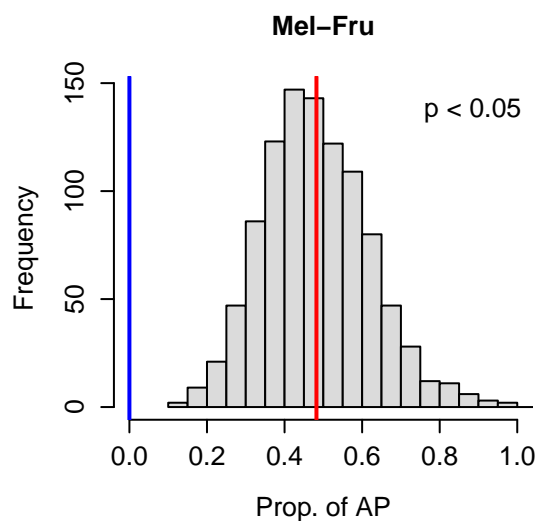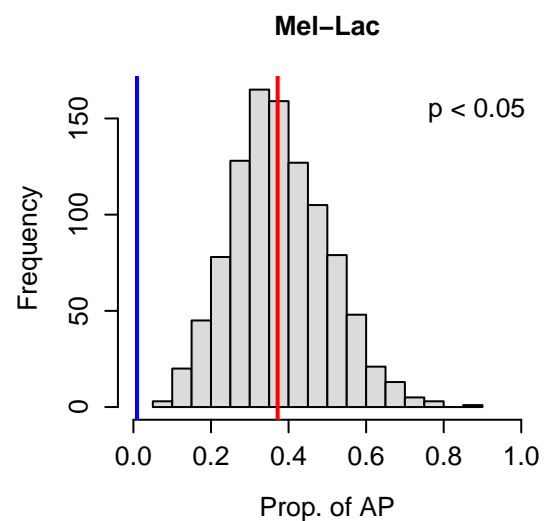

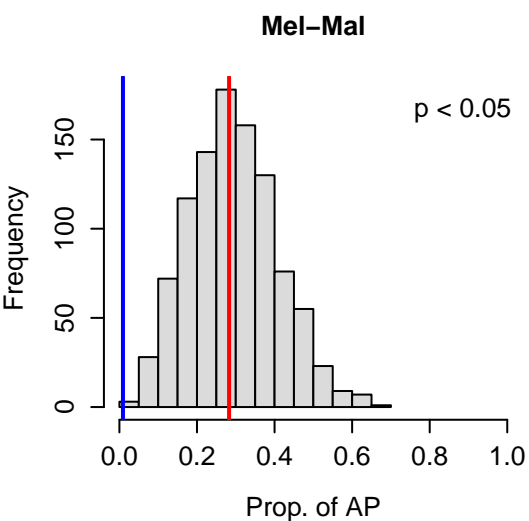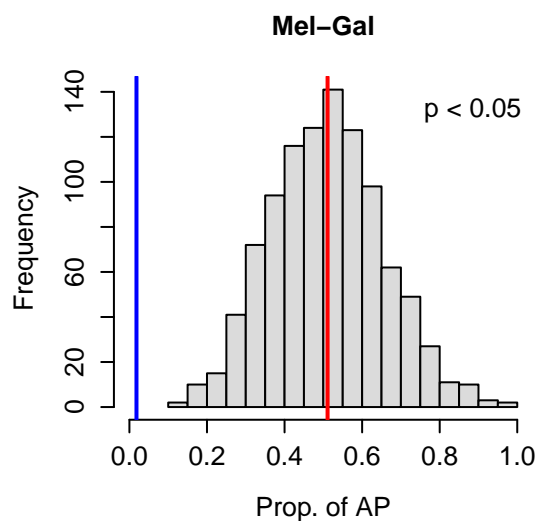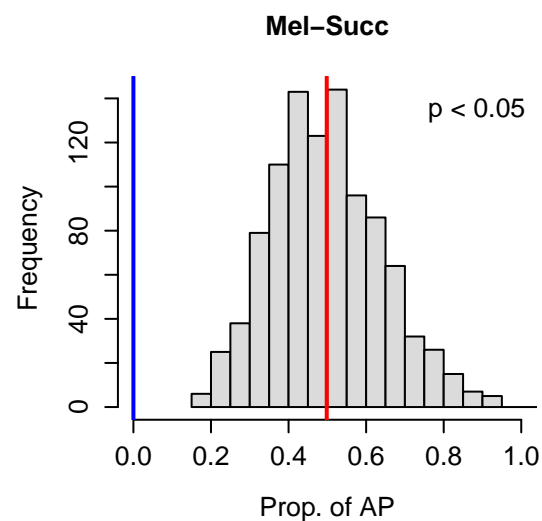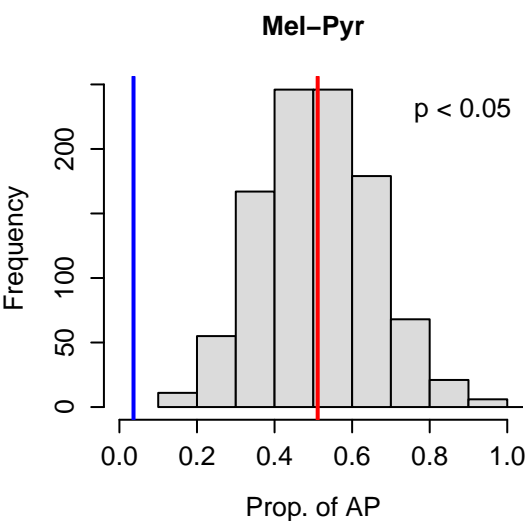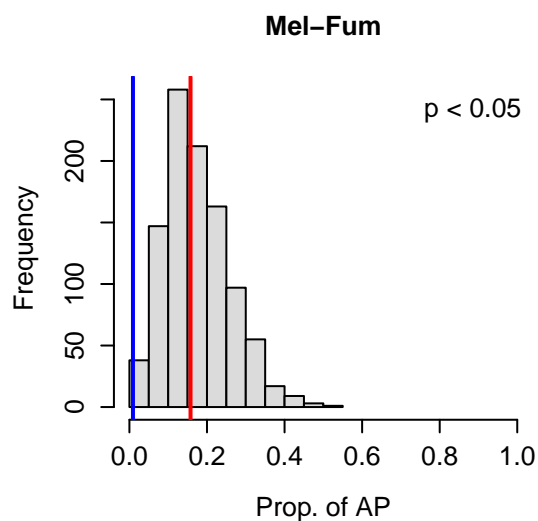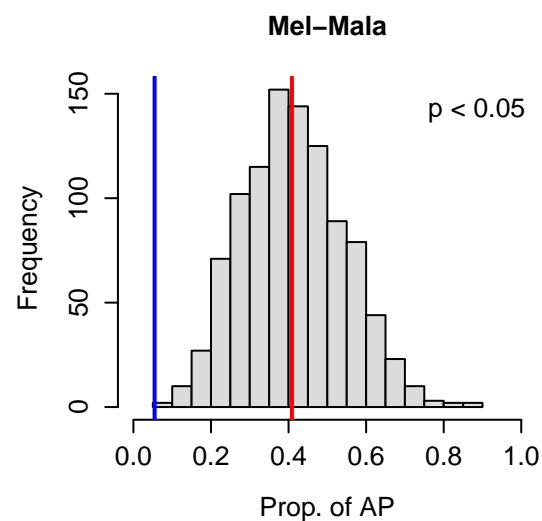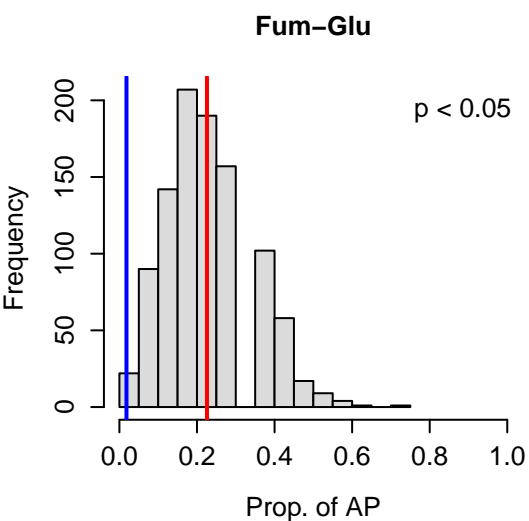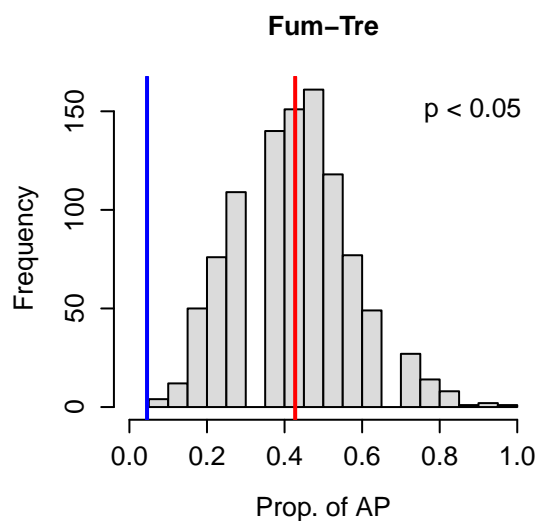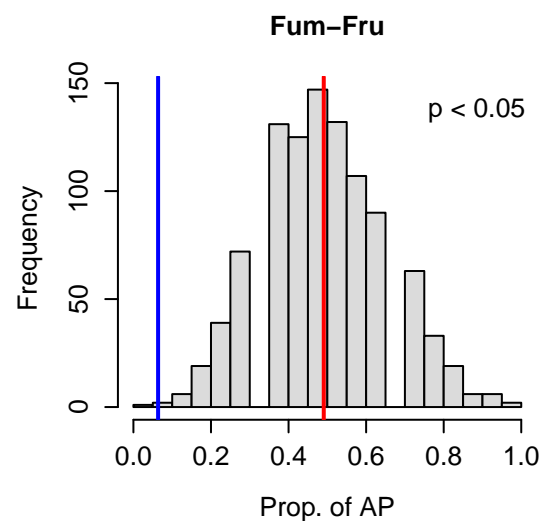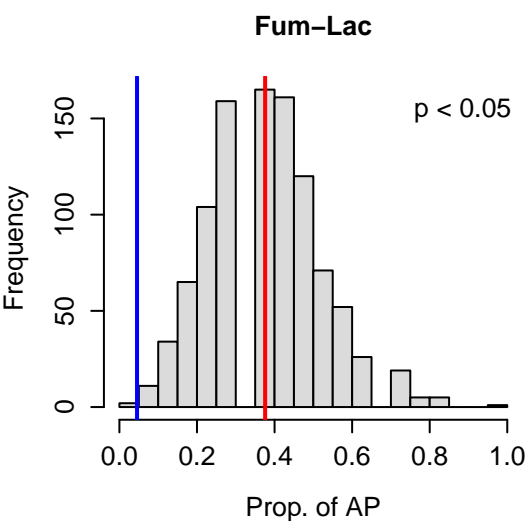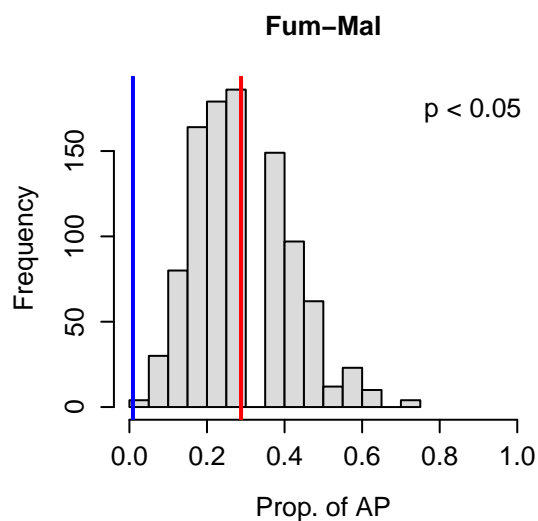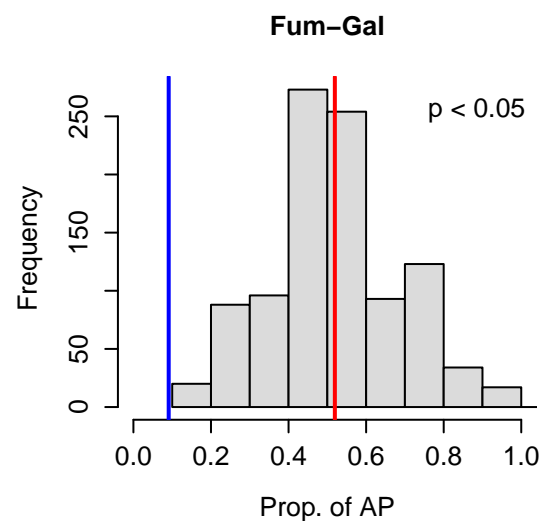

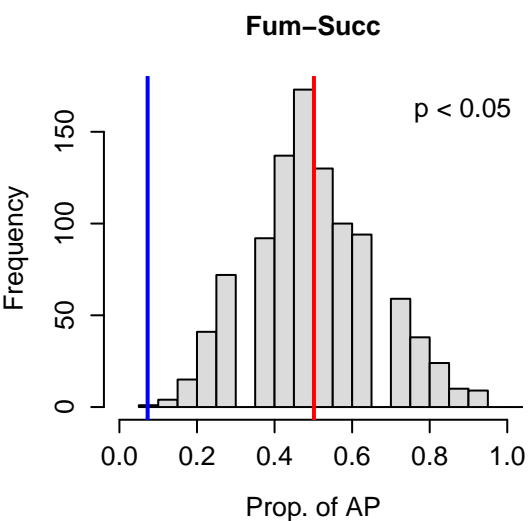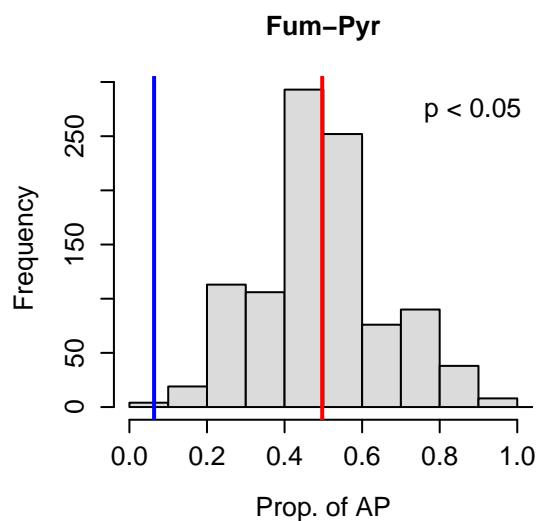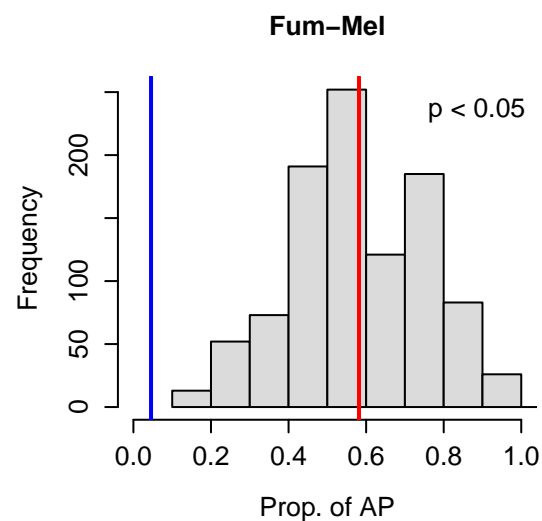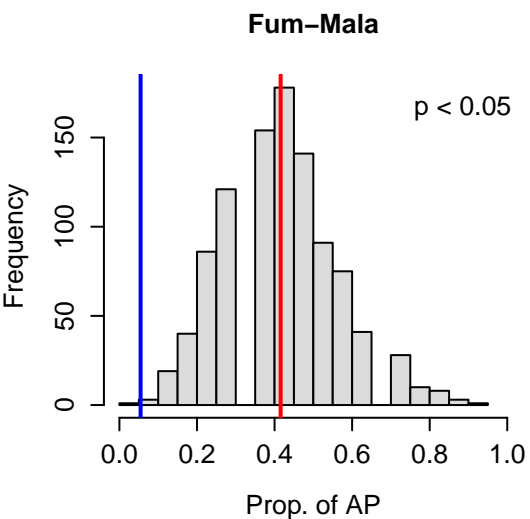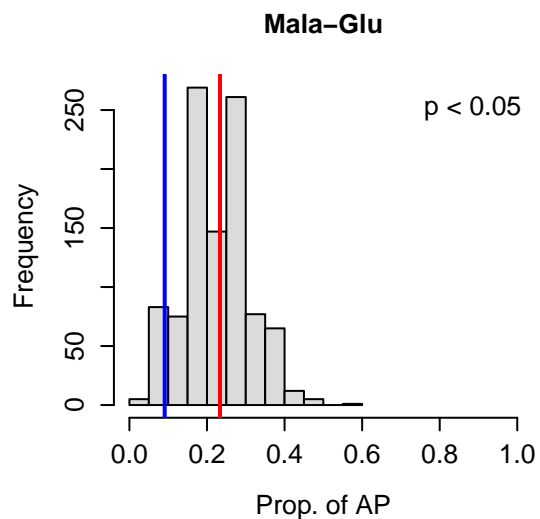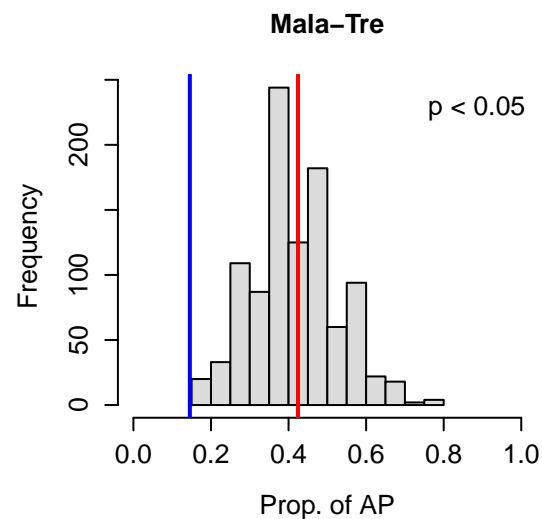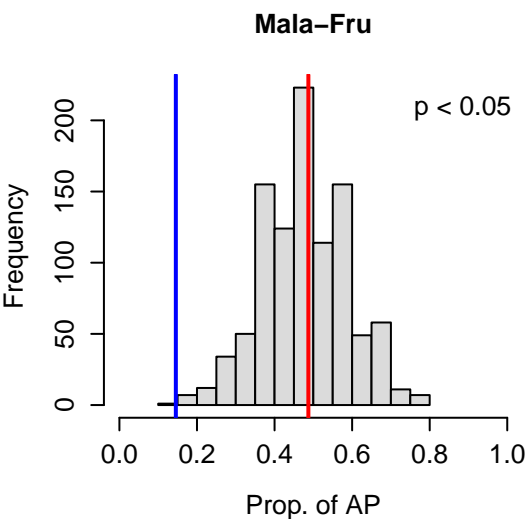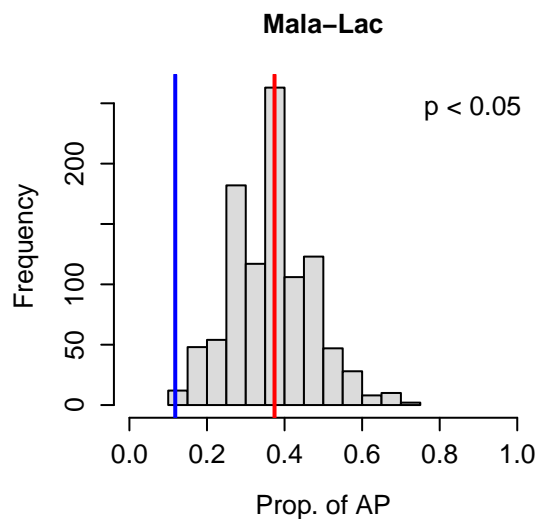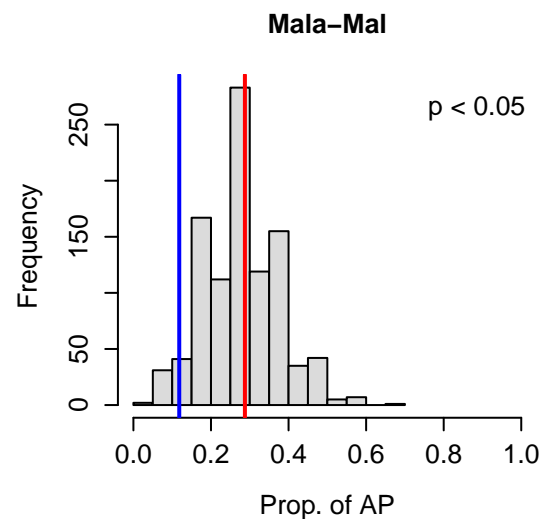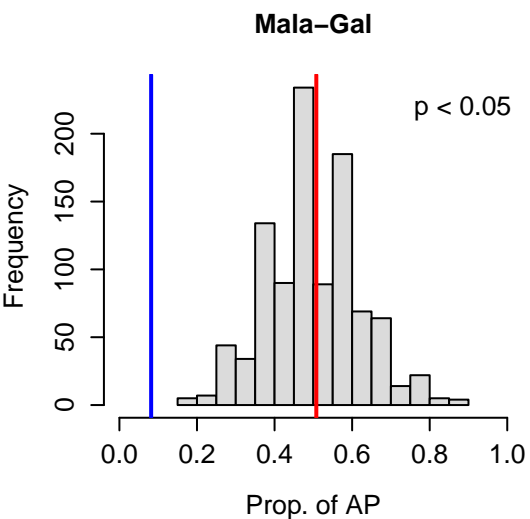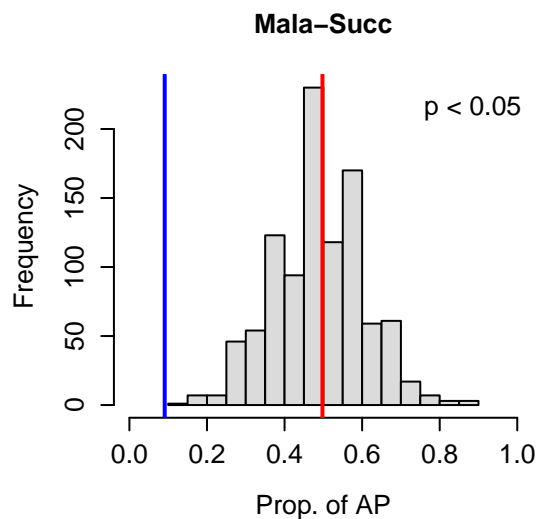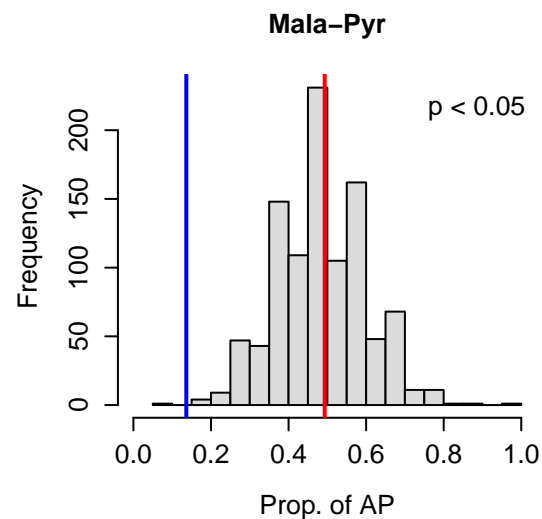

**Mala-Mel**

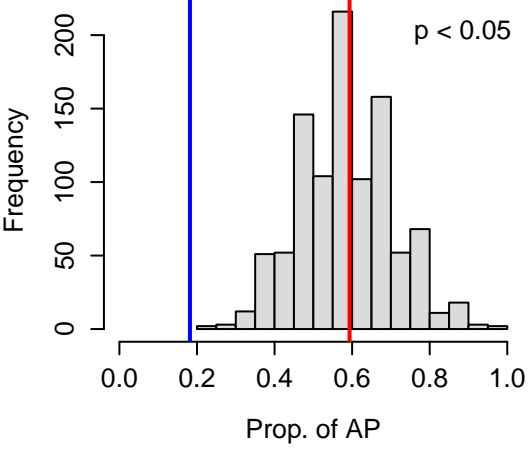

**Mala-Fum**

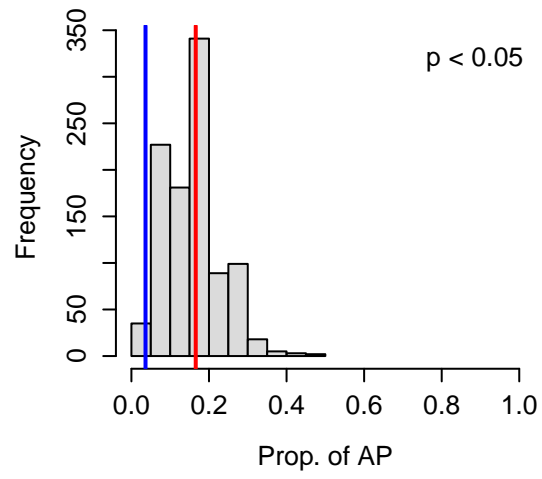

Supplement: Supplementary file 7 [file EVO-72-2202-s007.pdf]

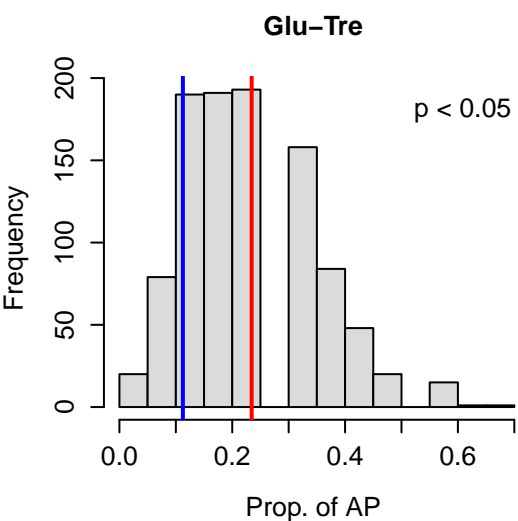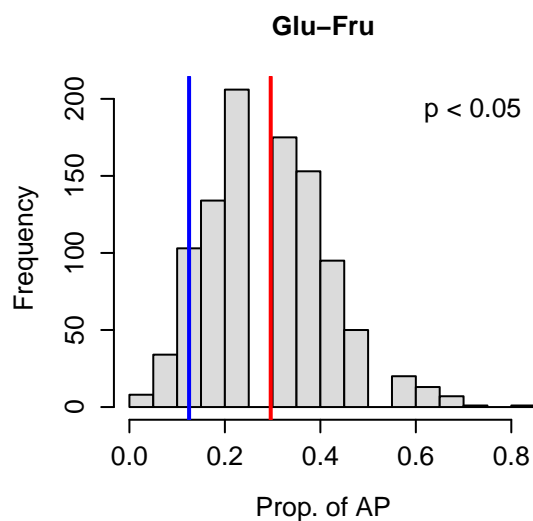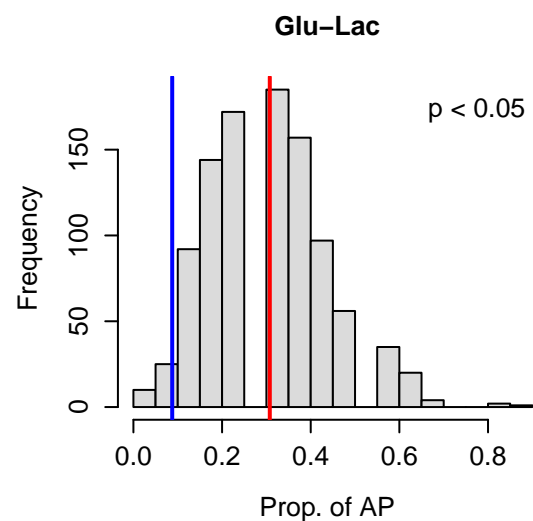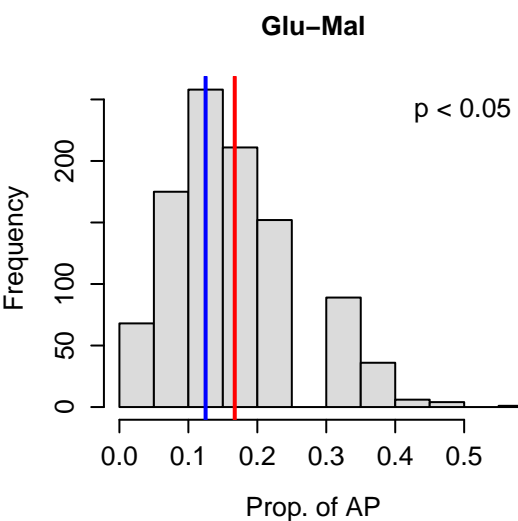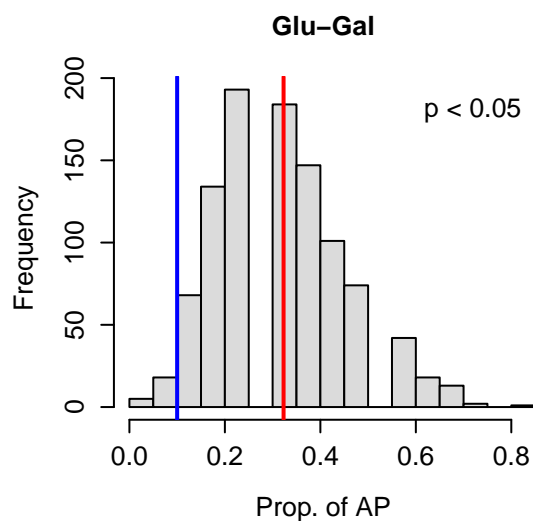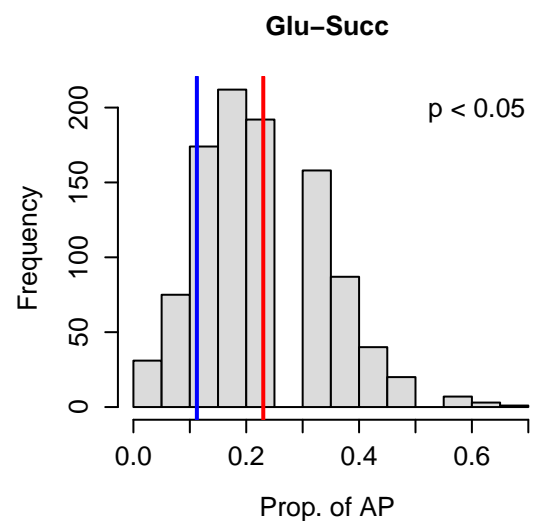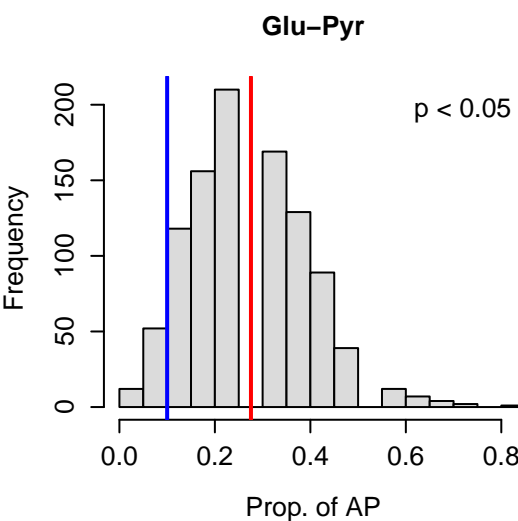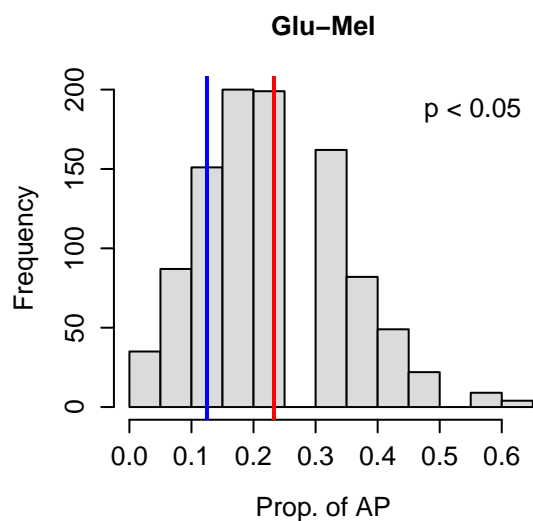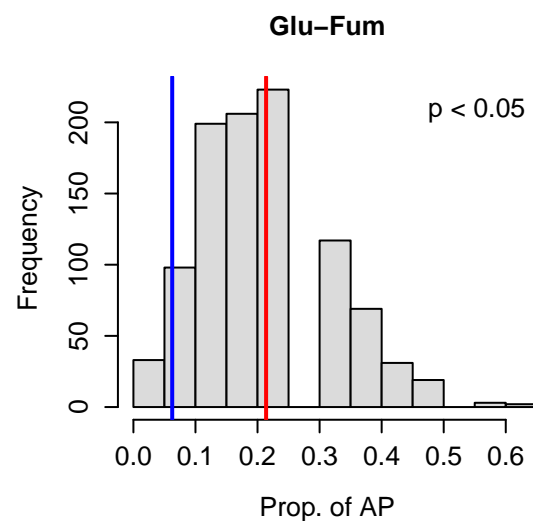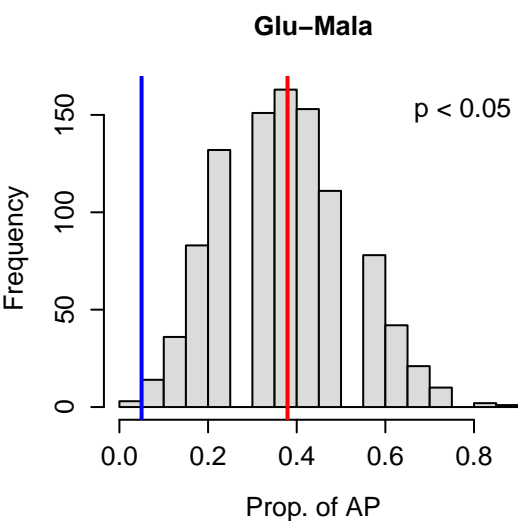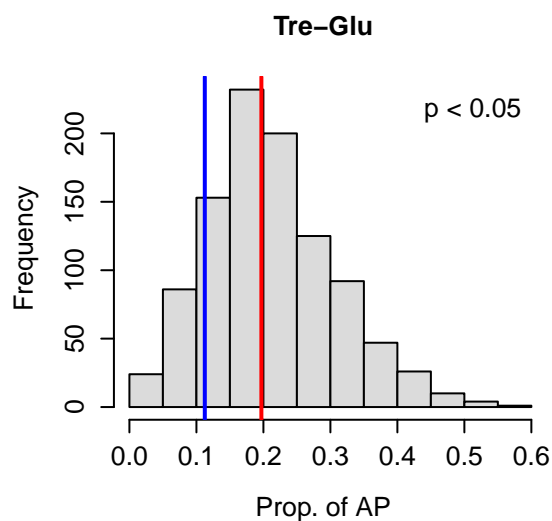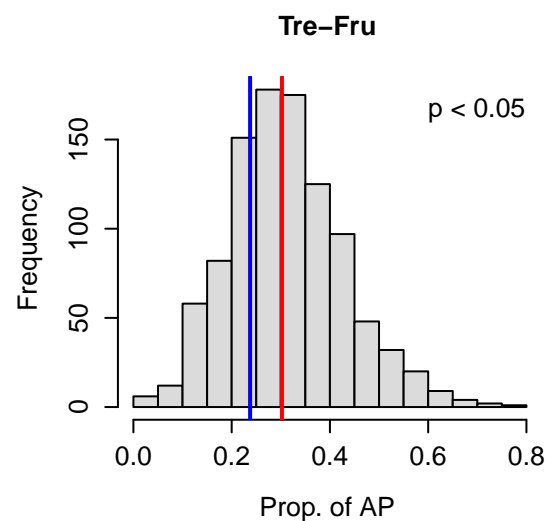

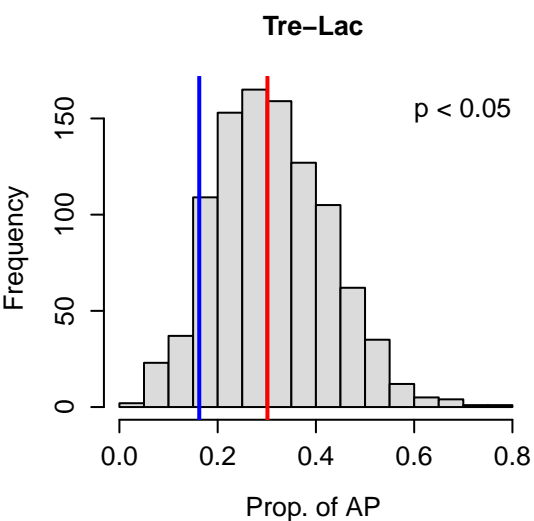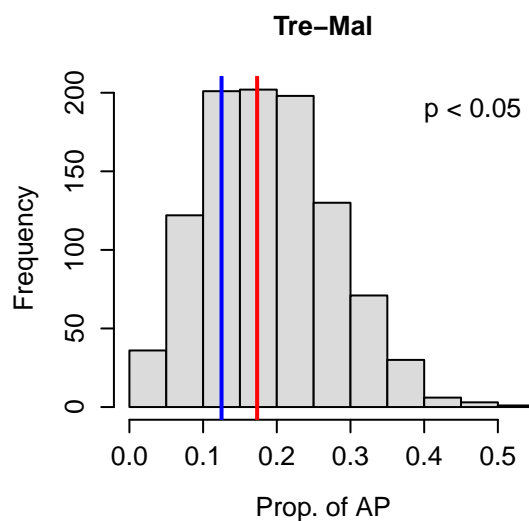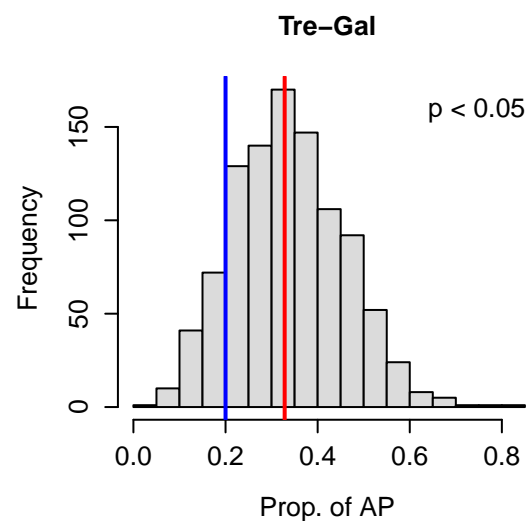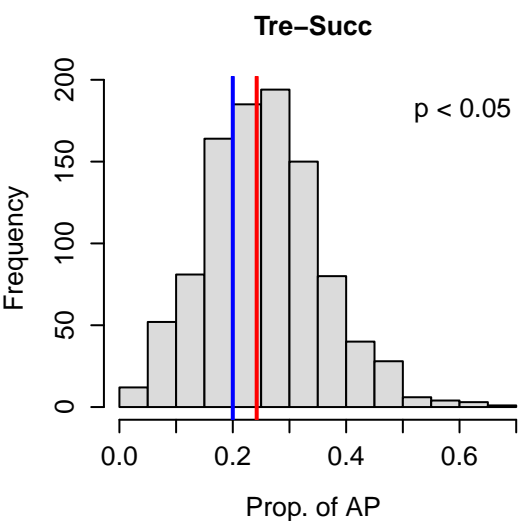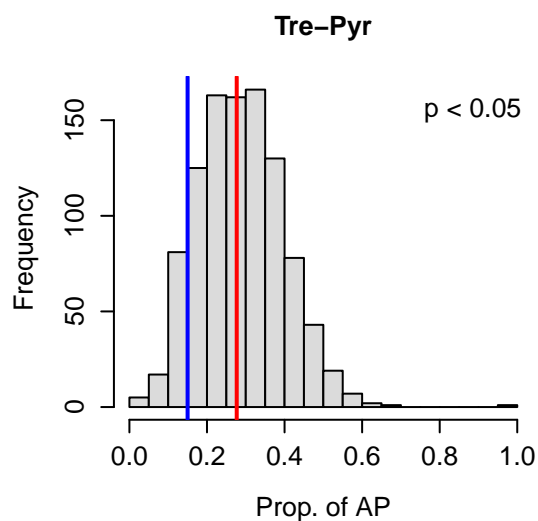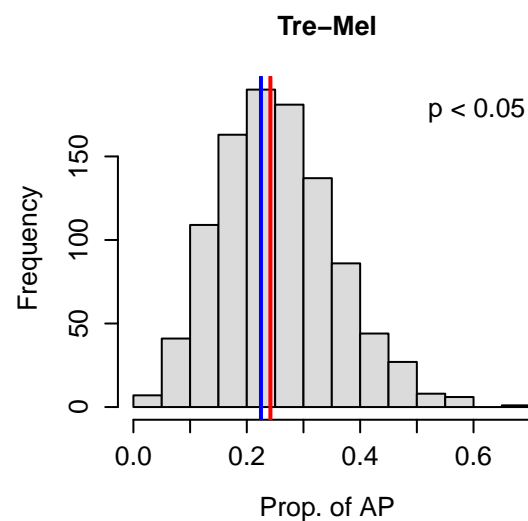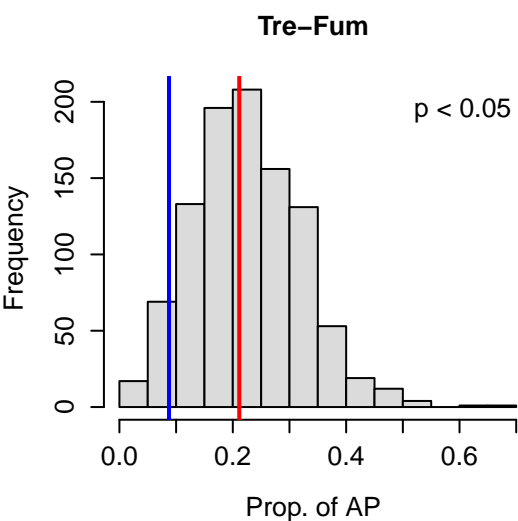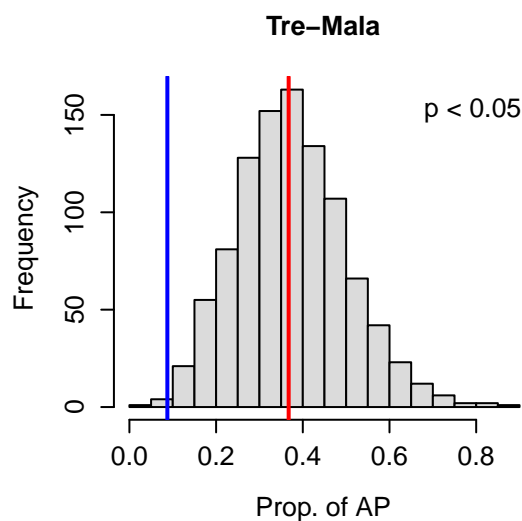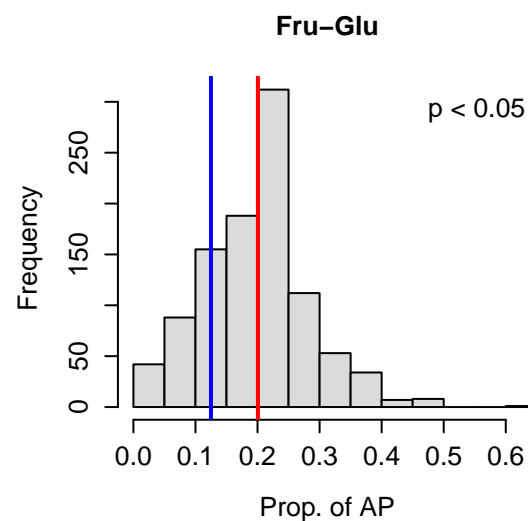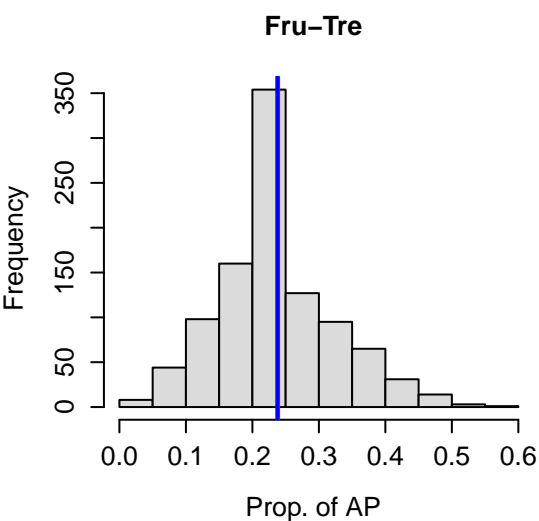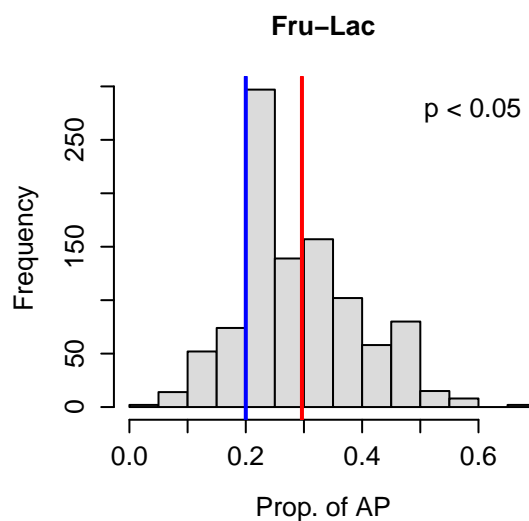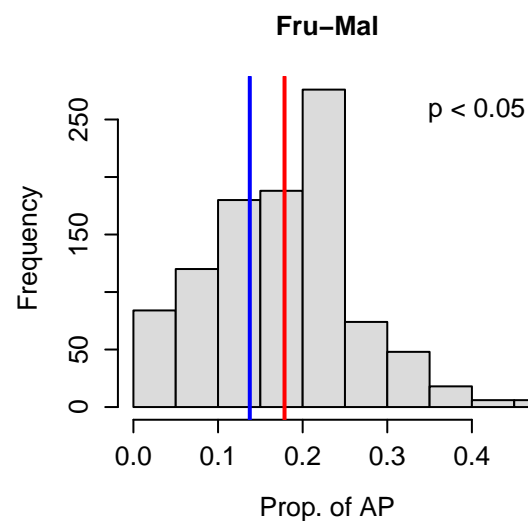

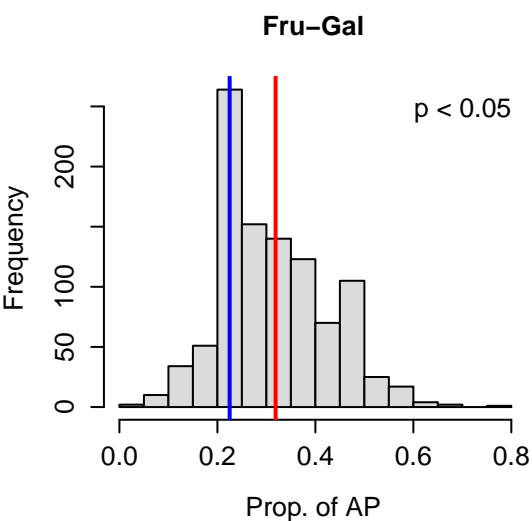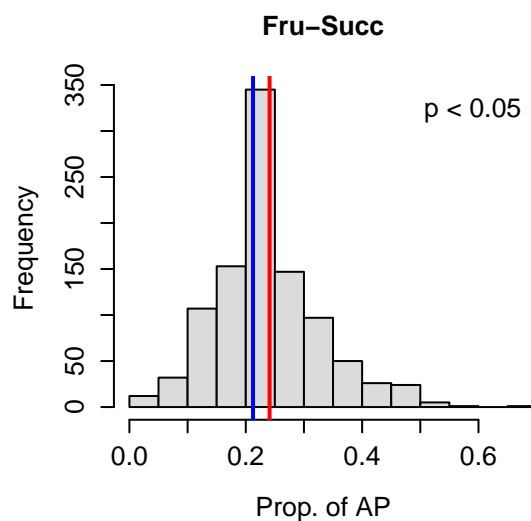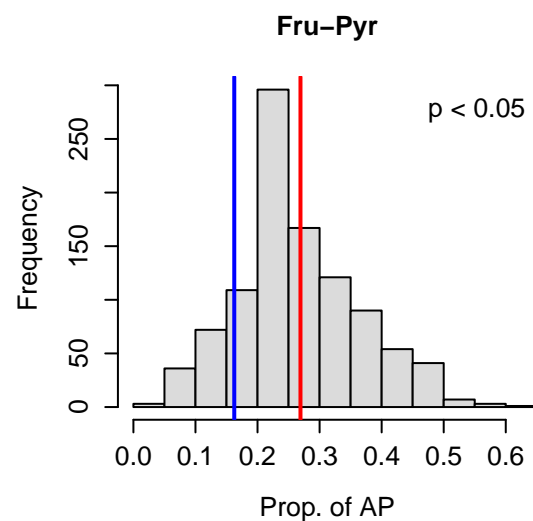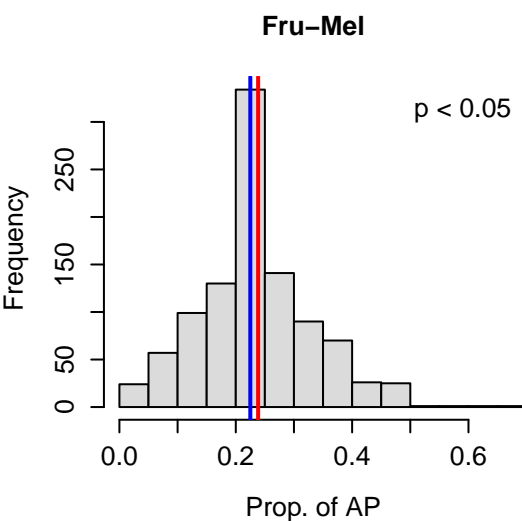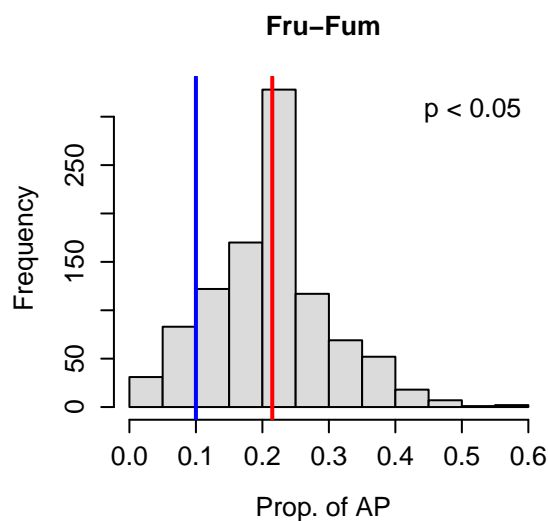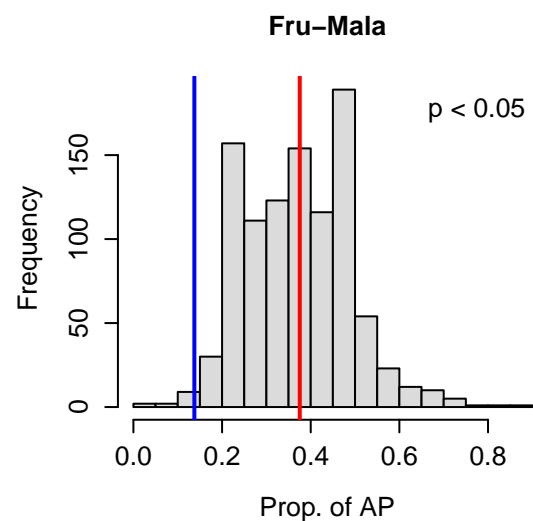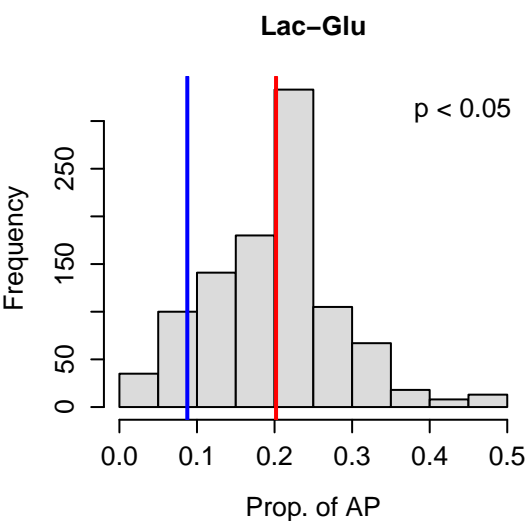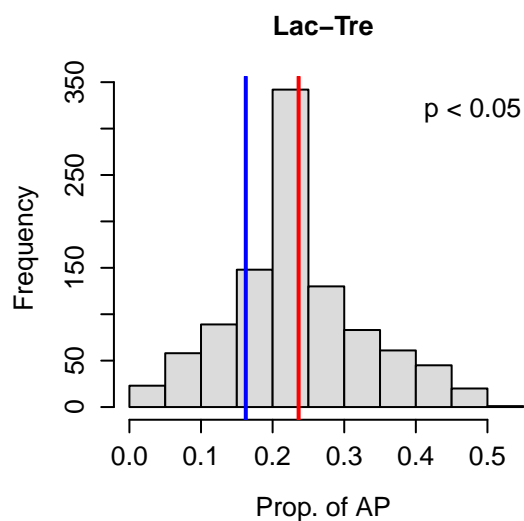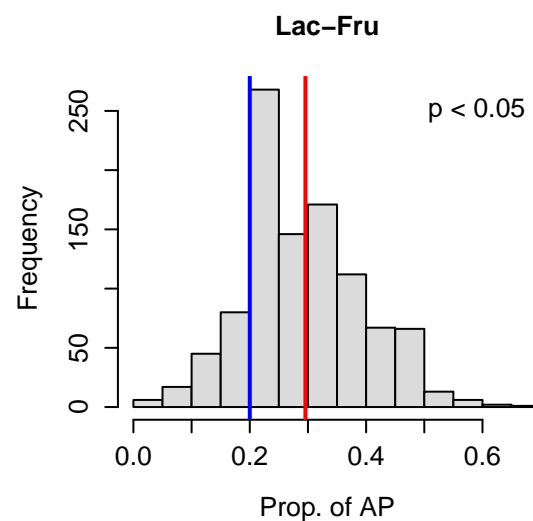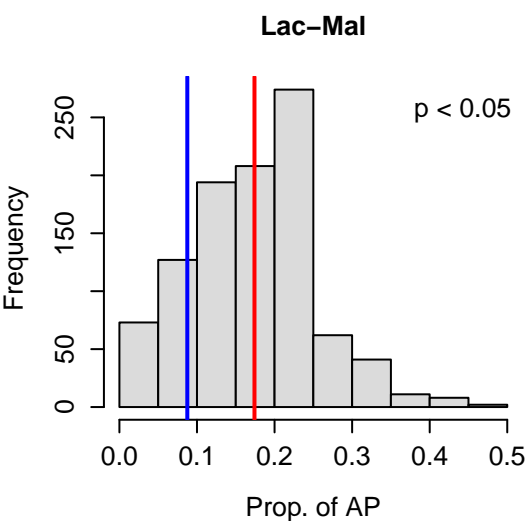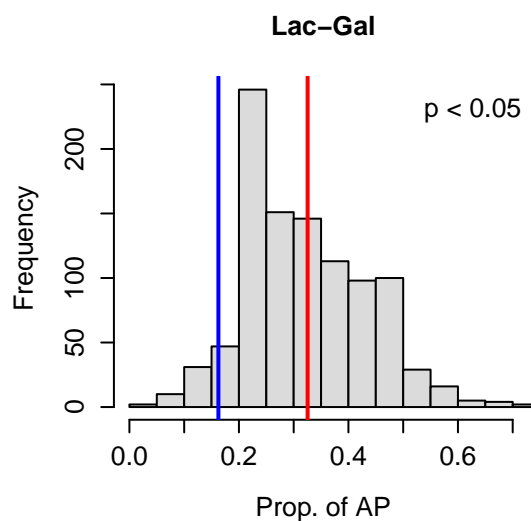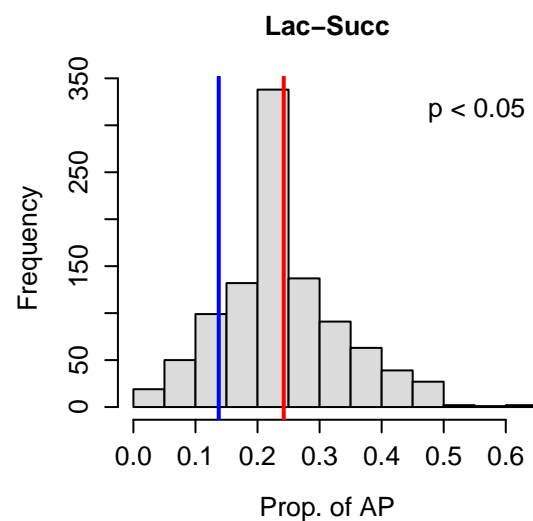

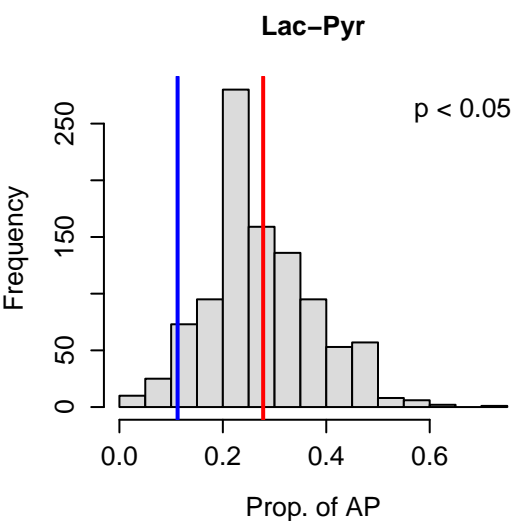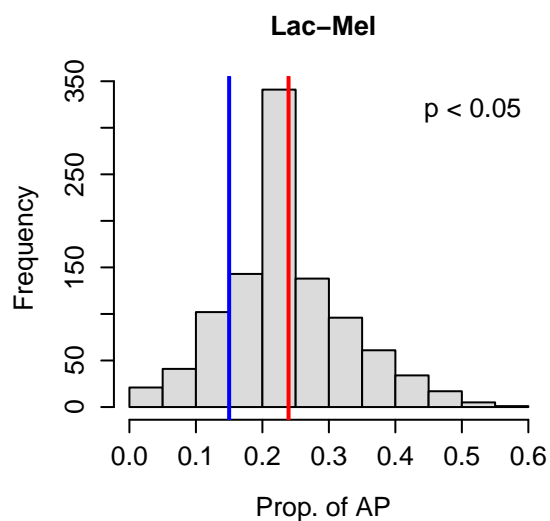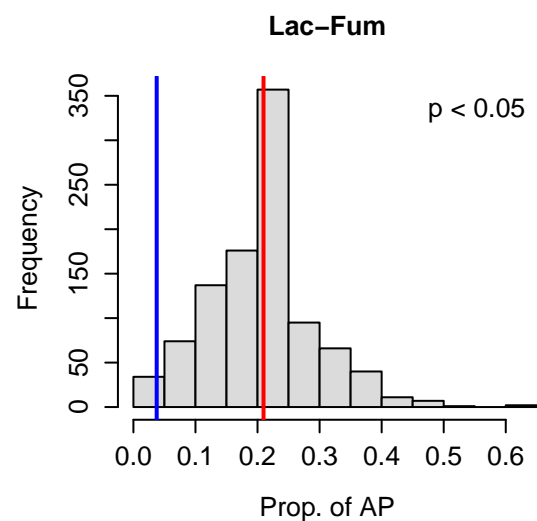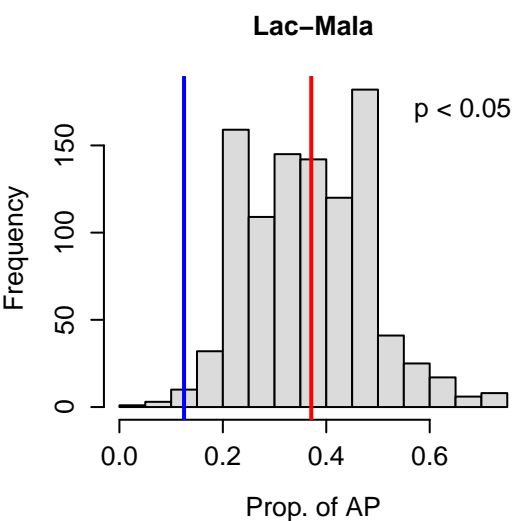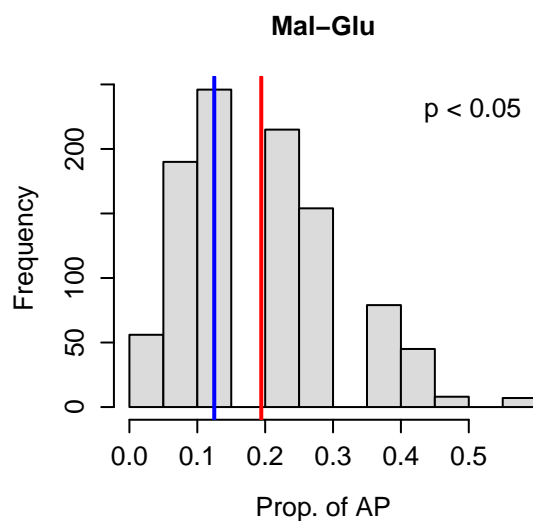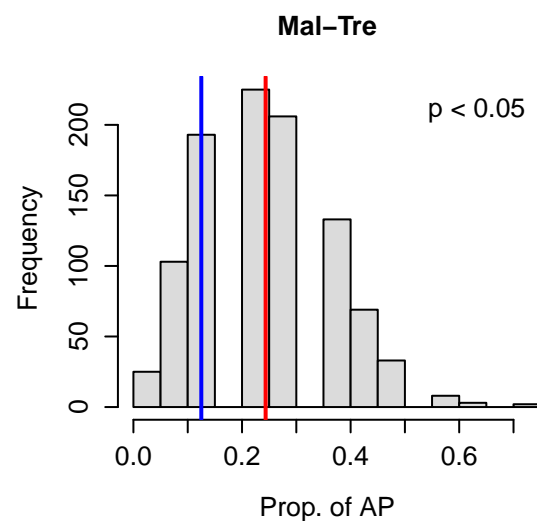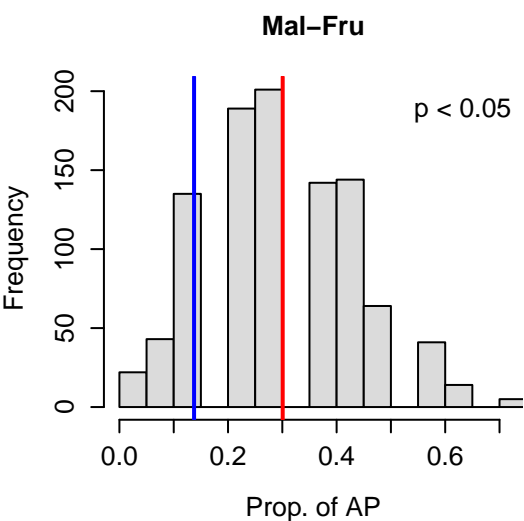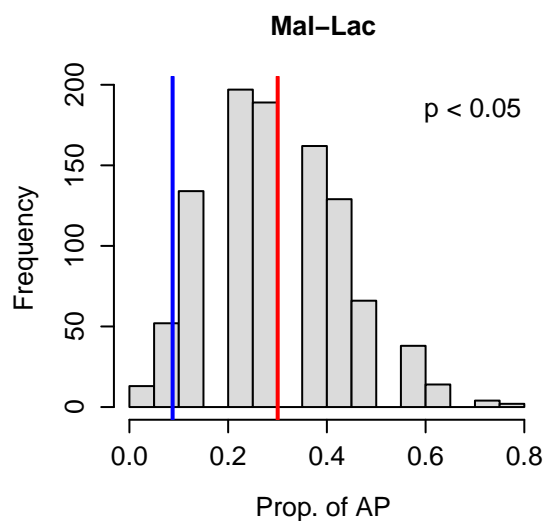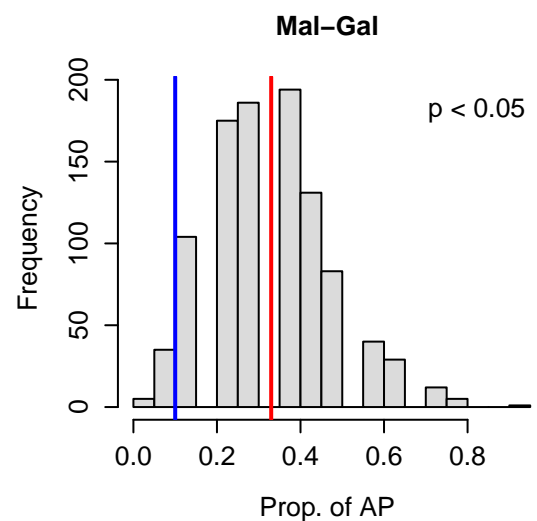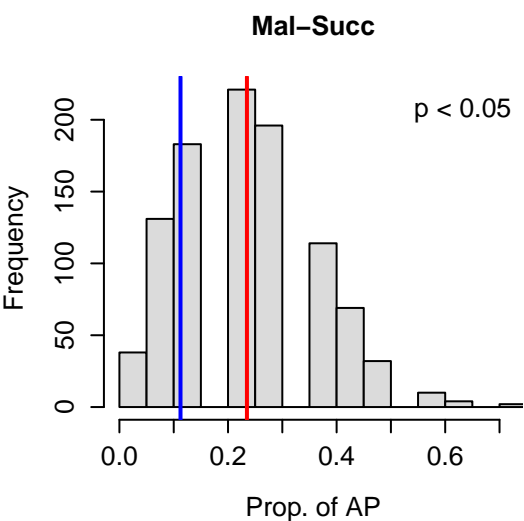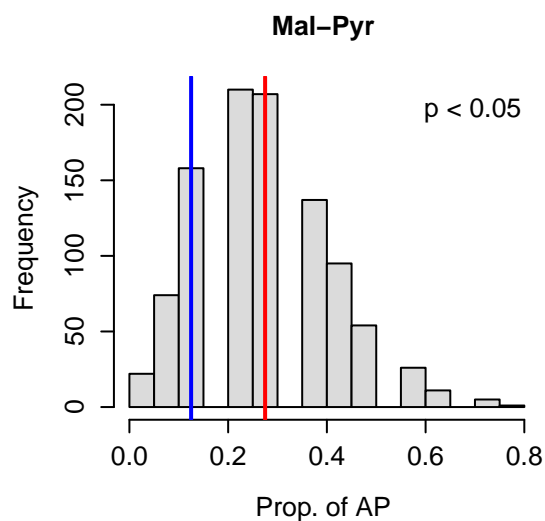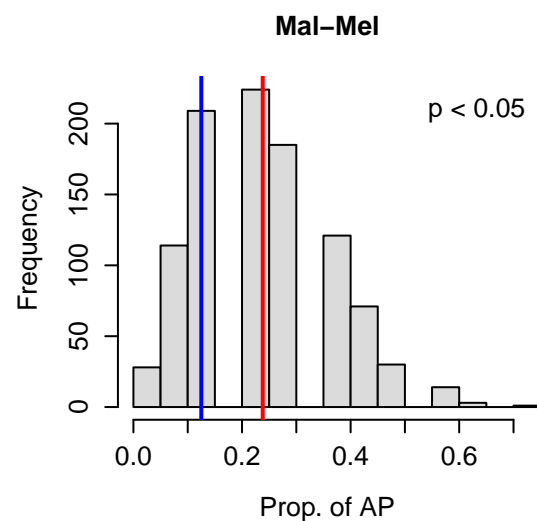

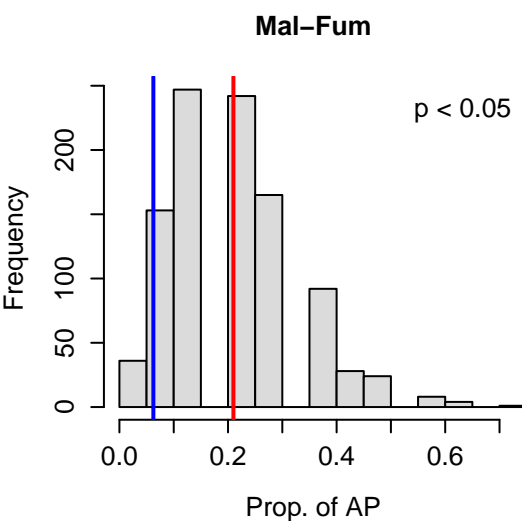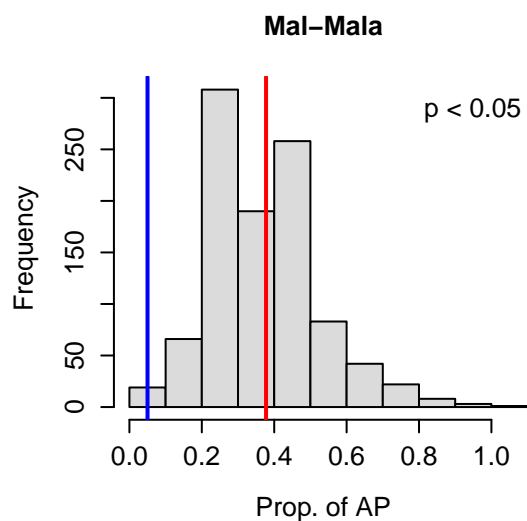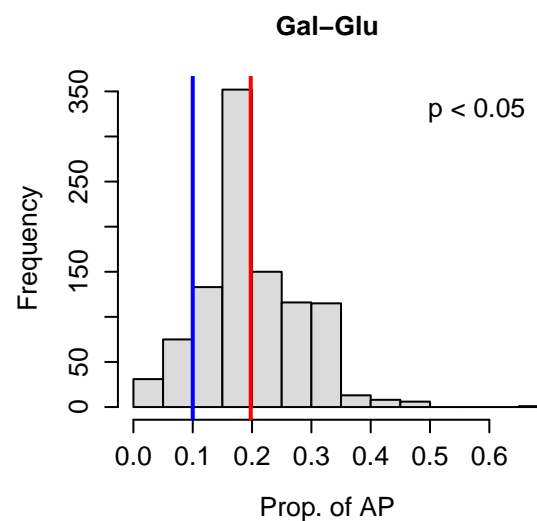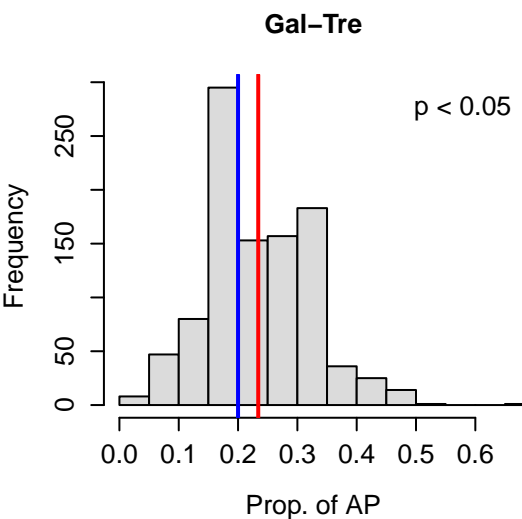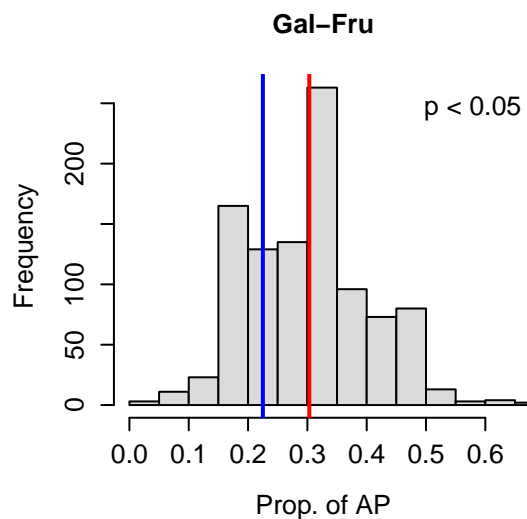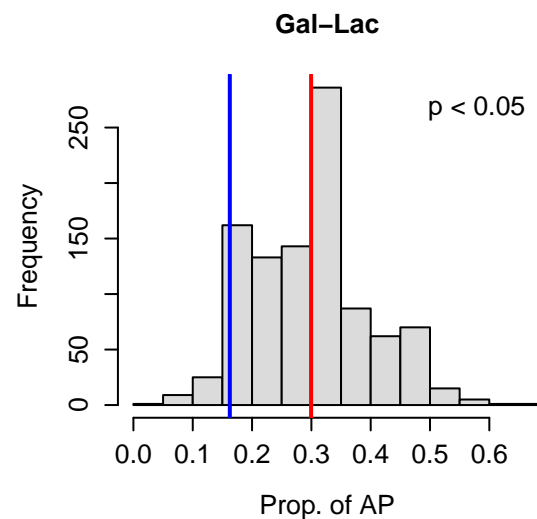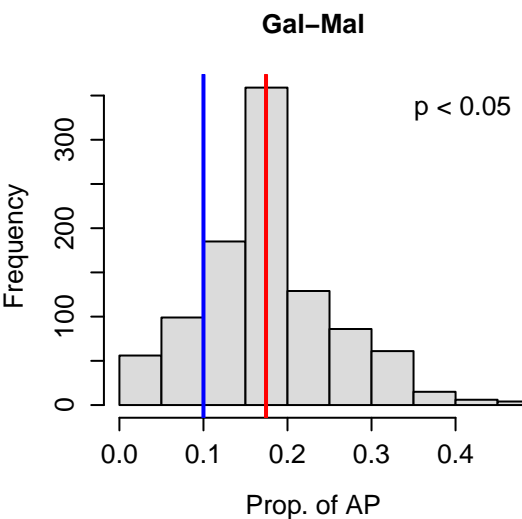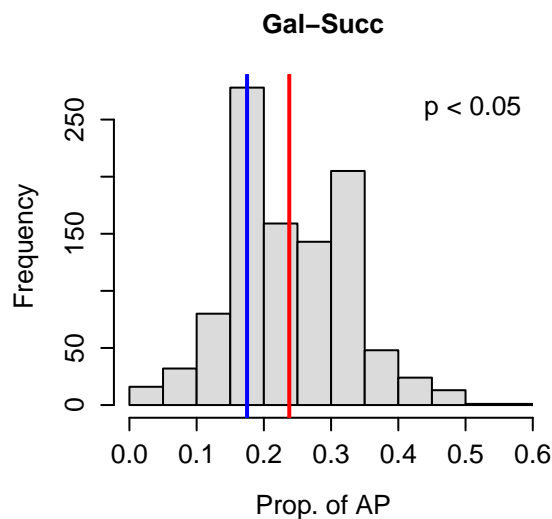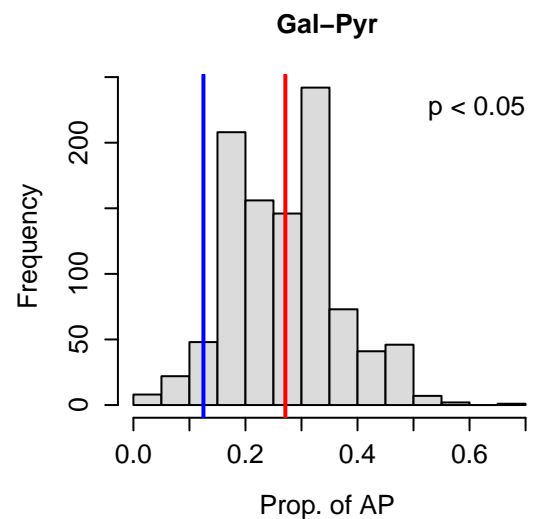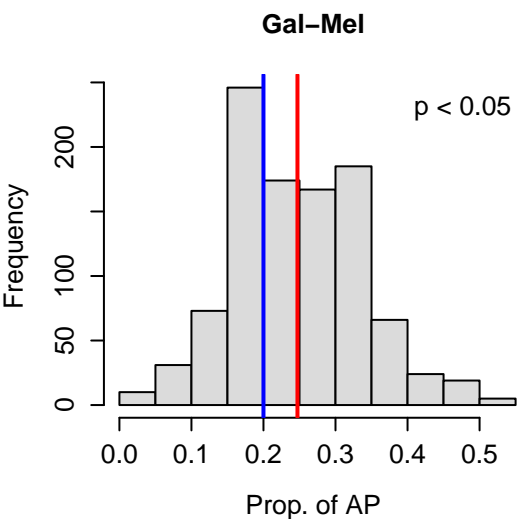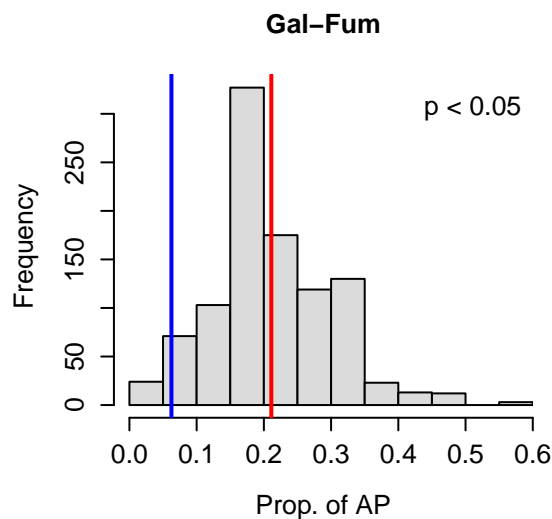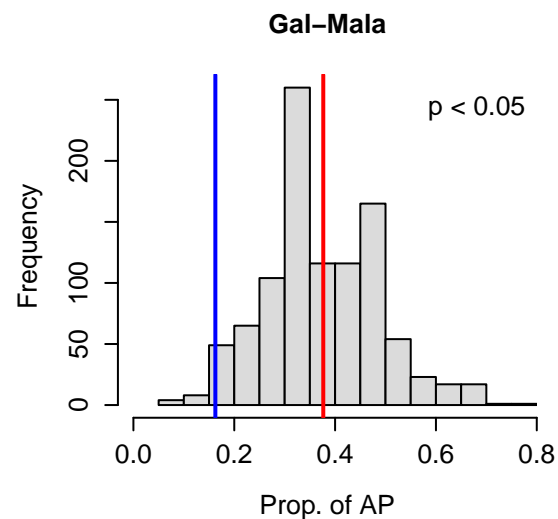

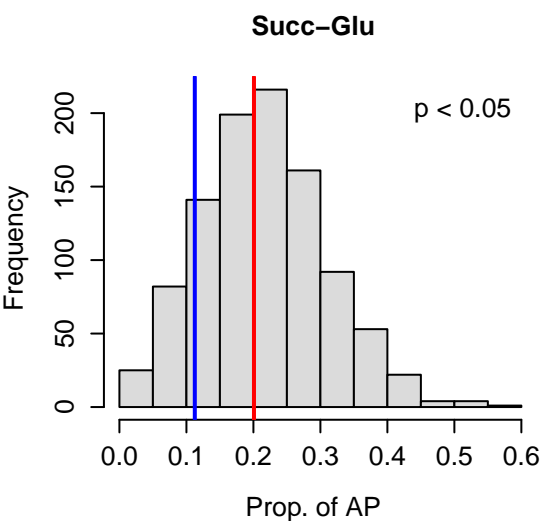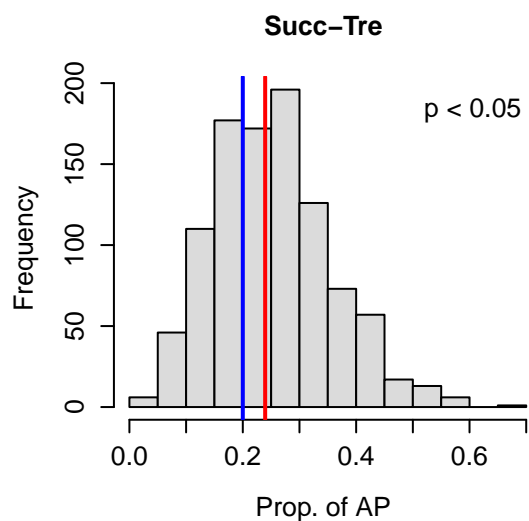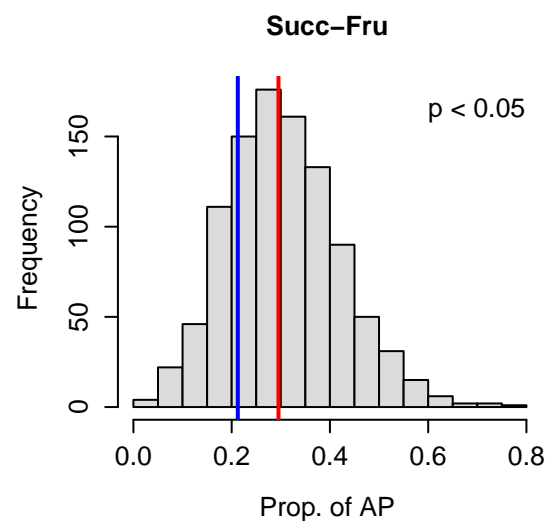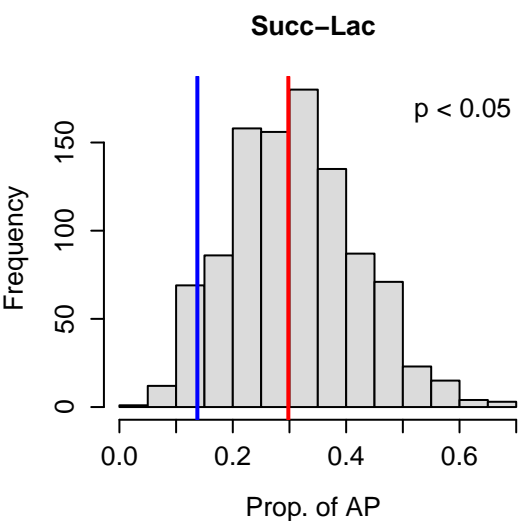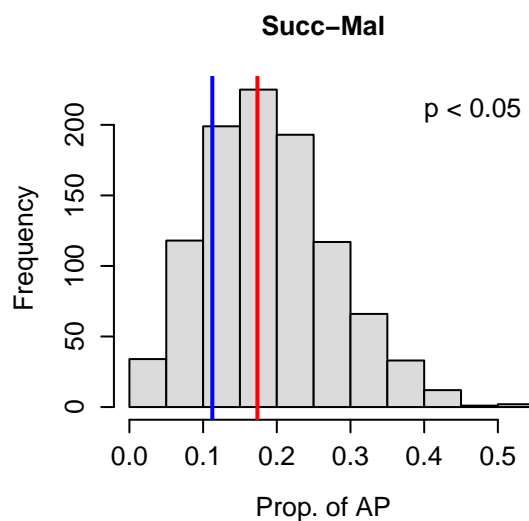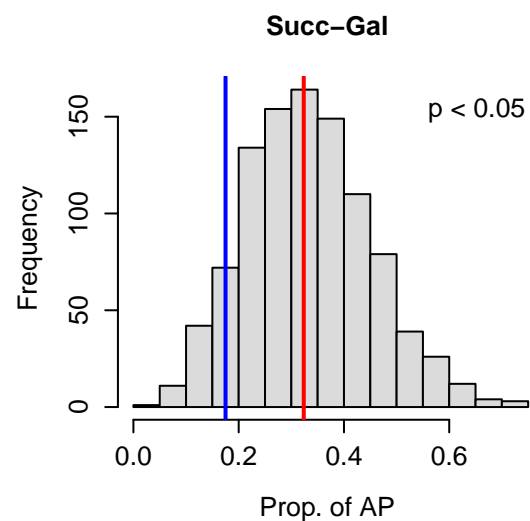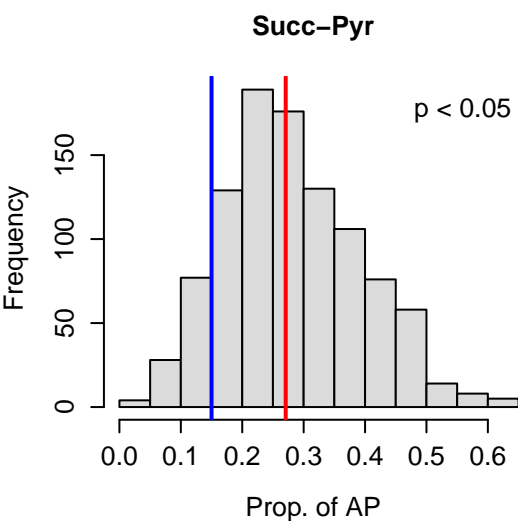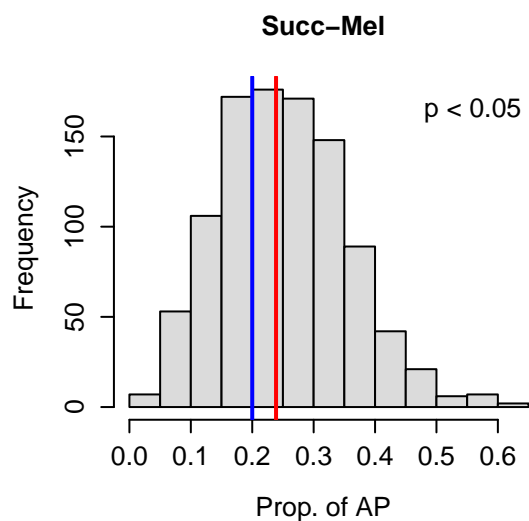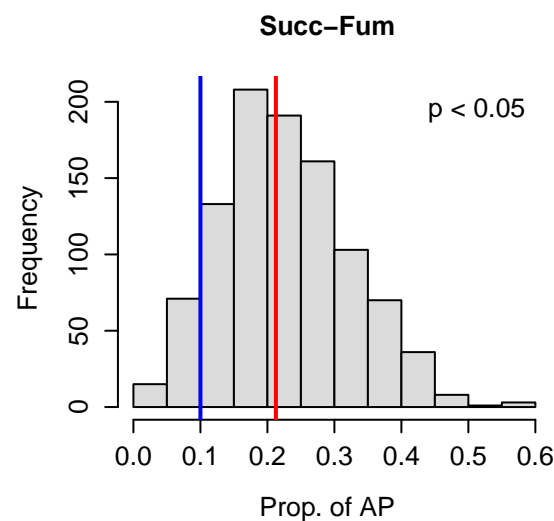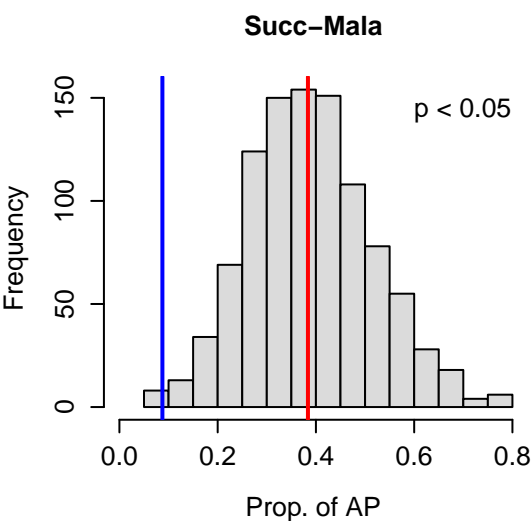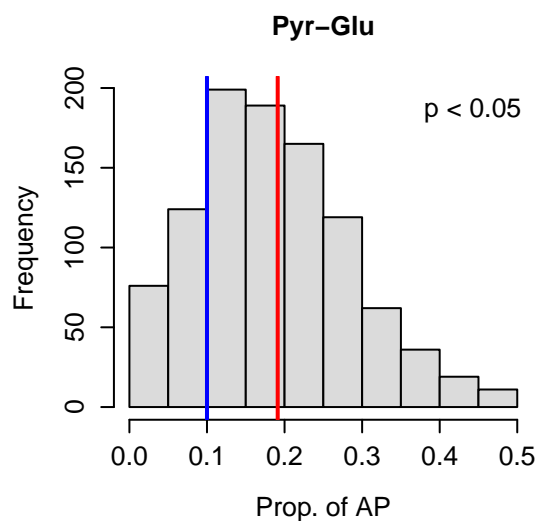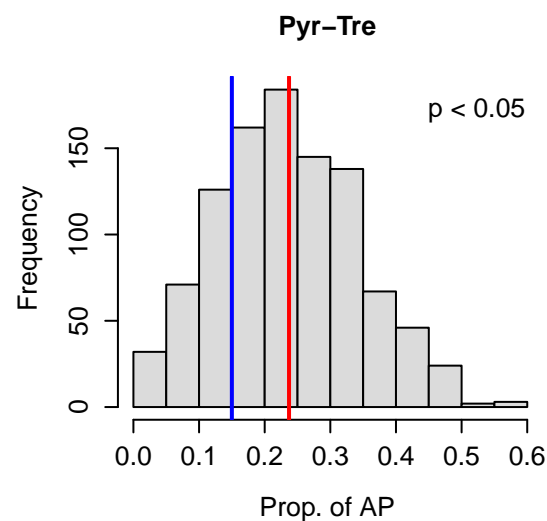

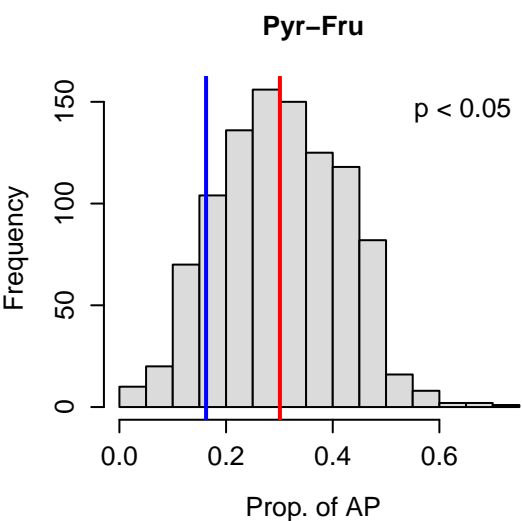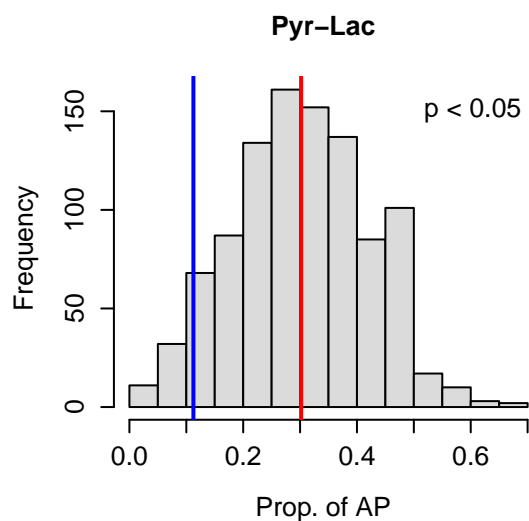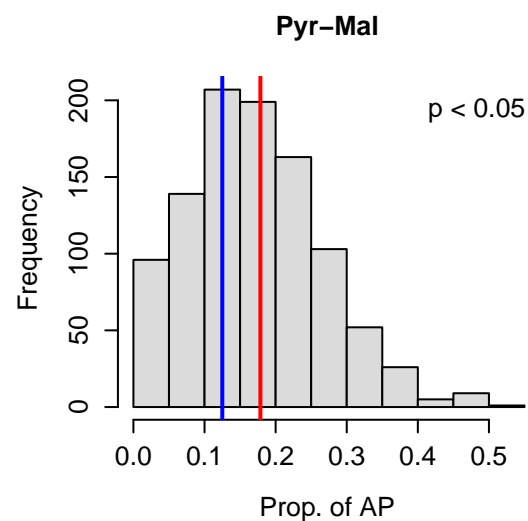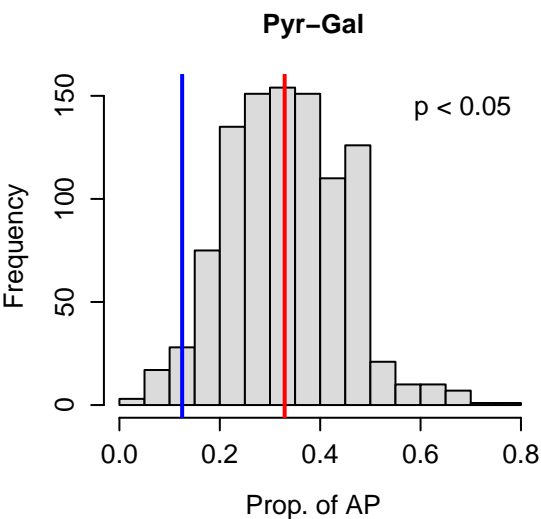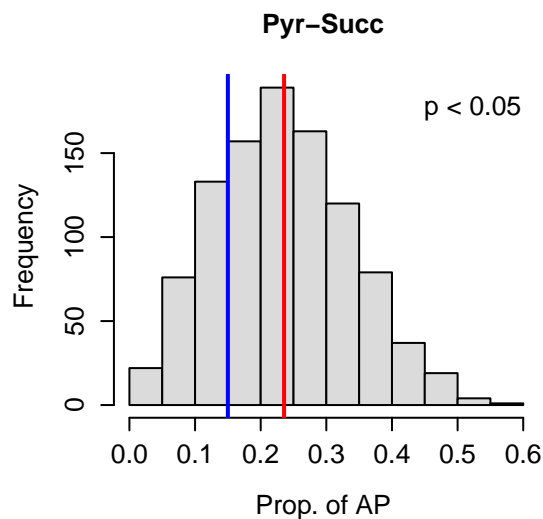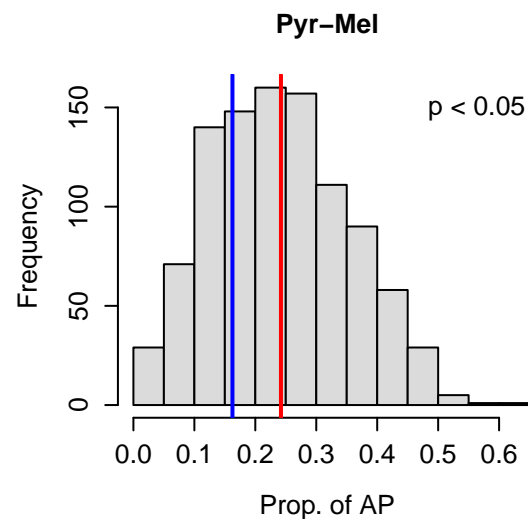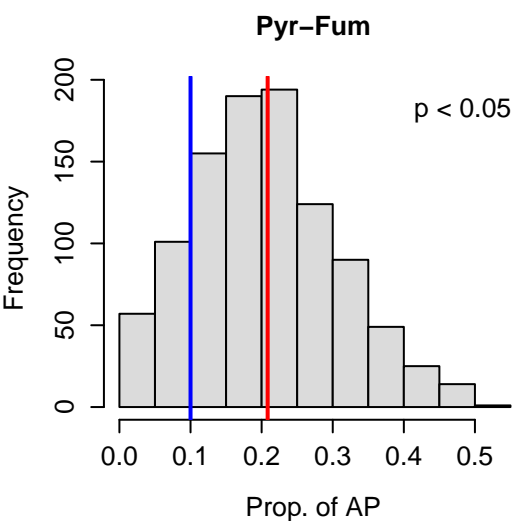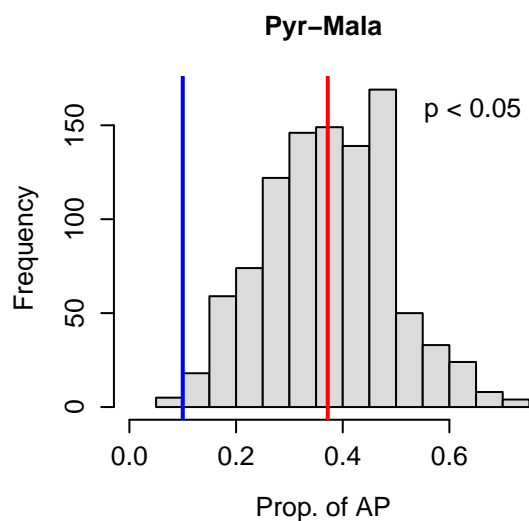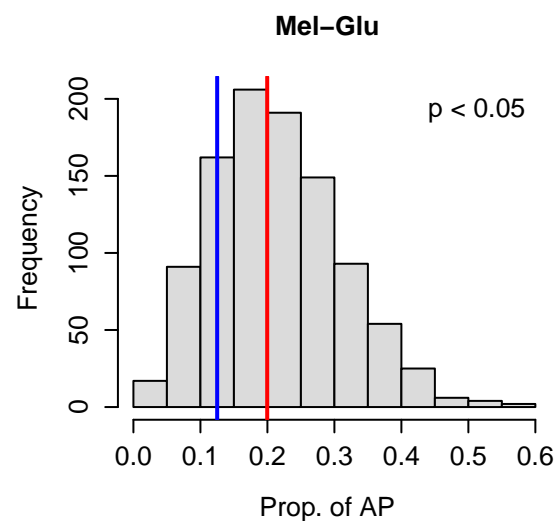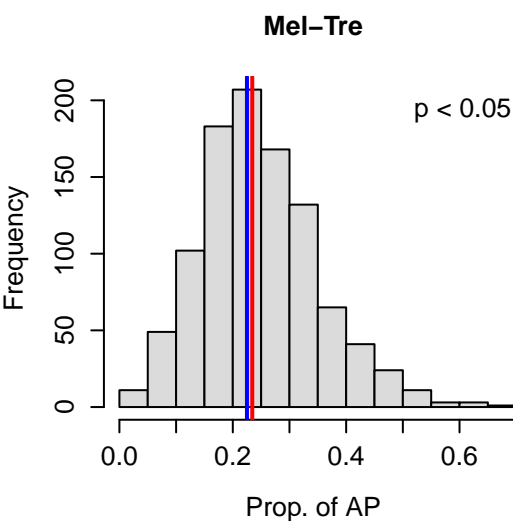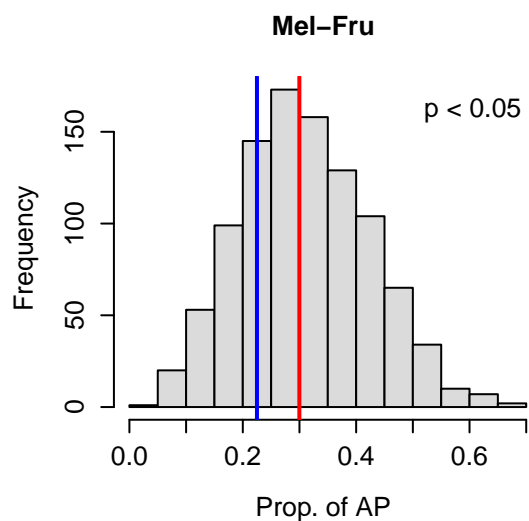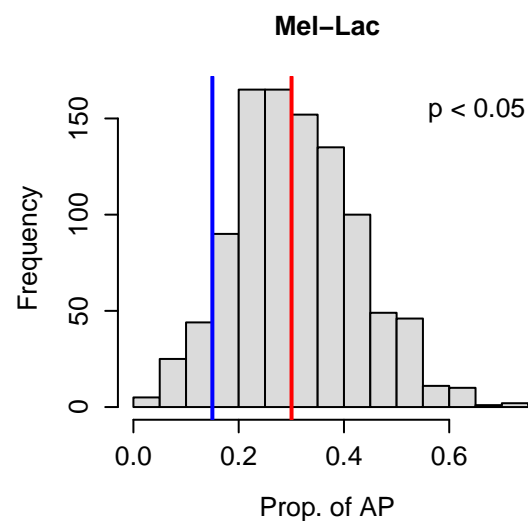

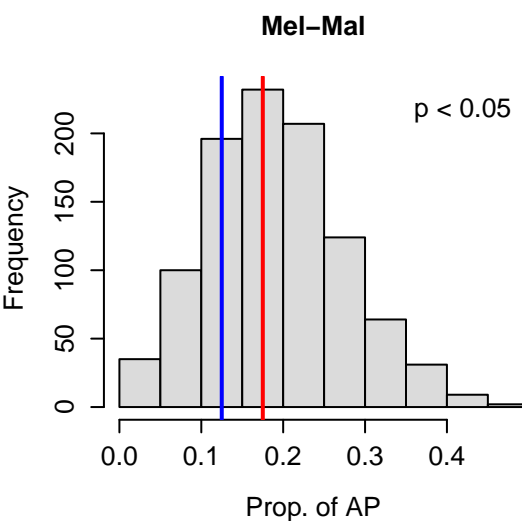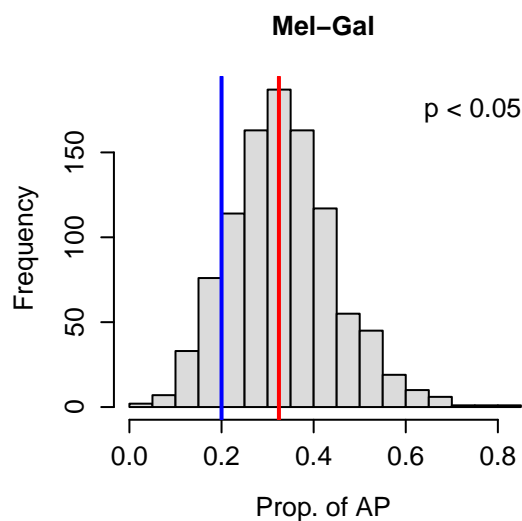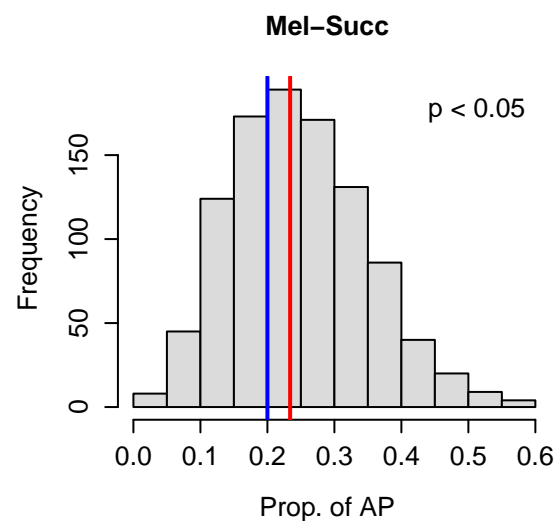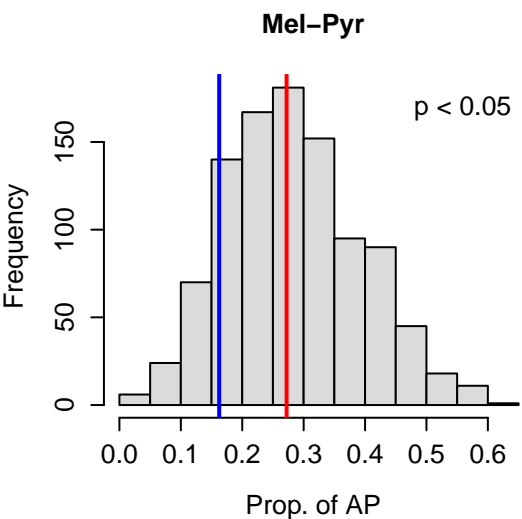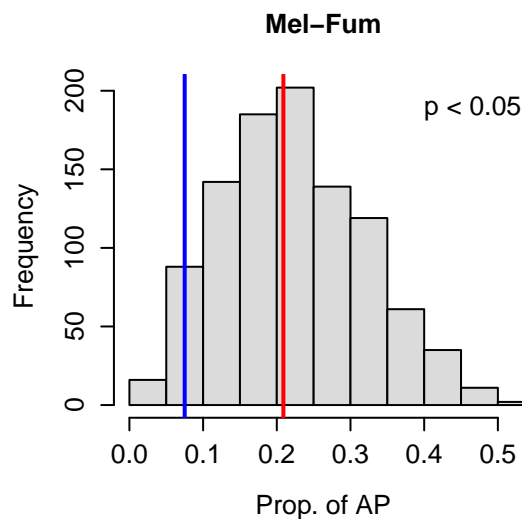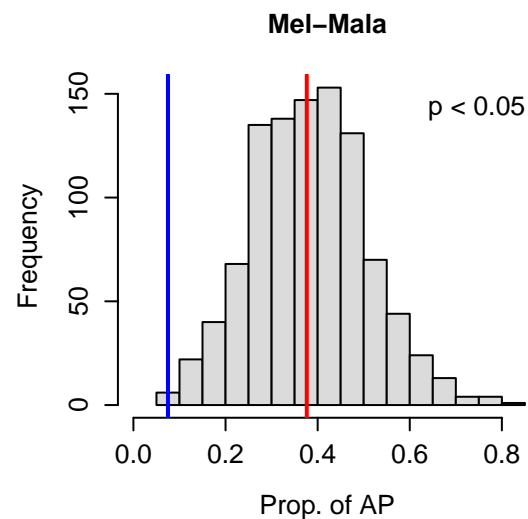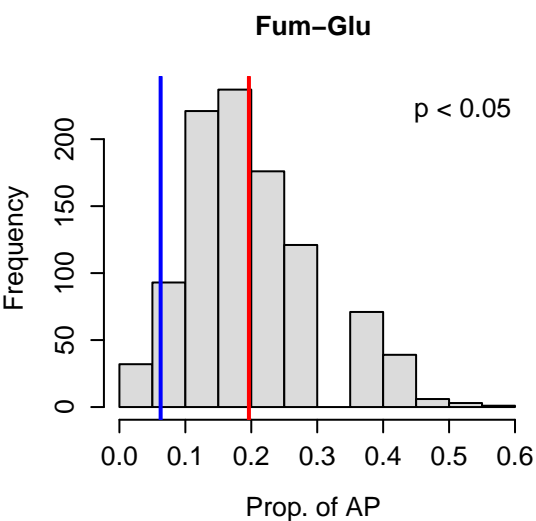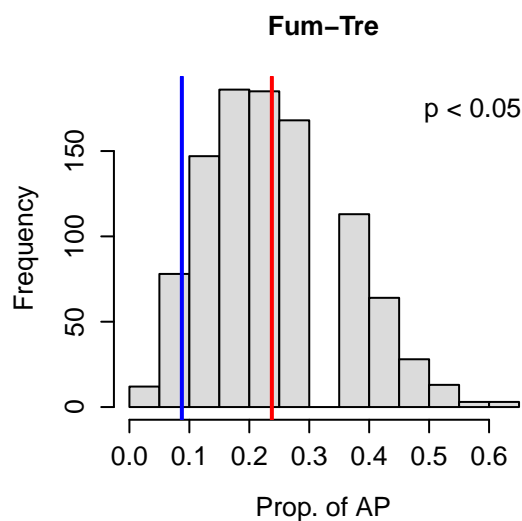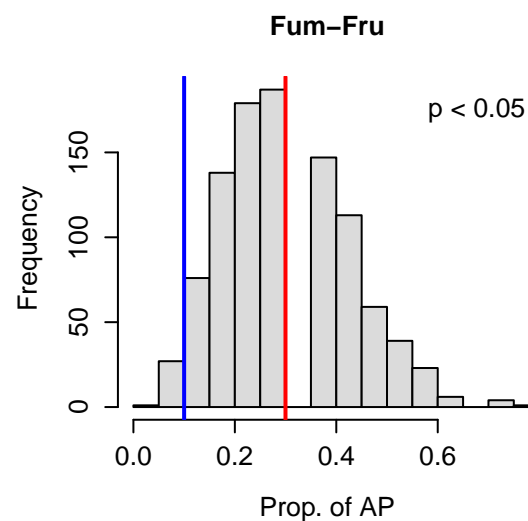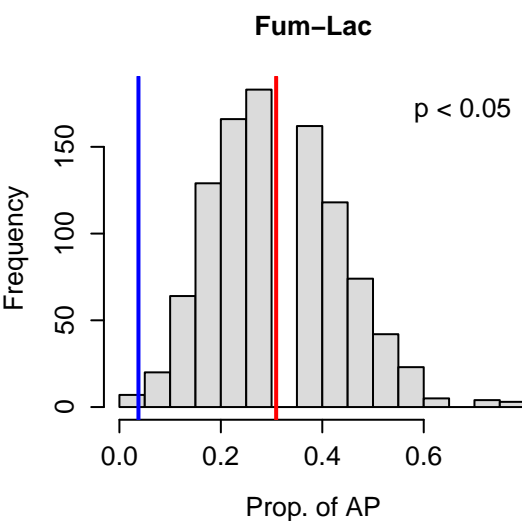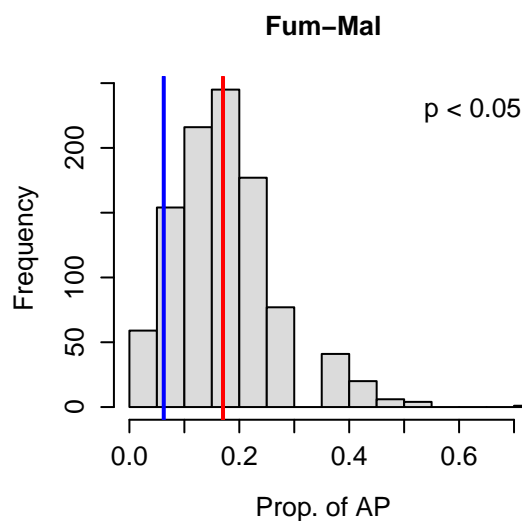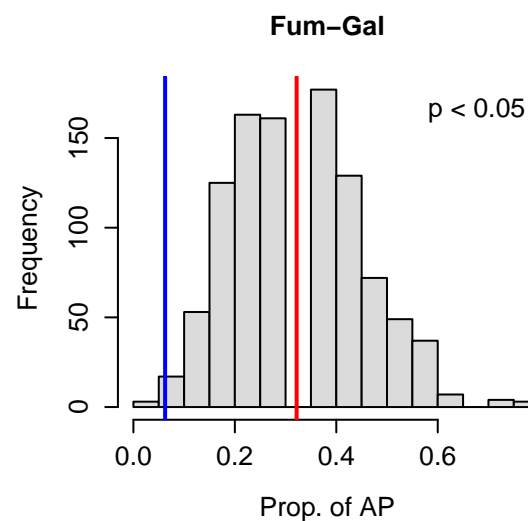

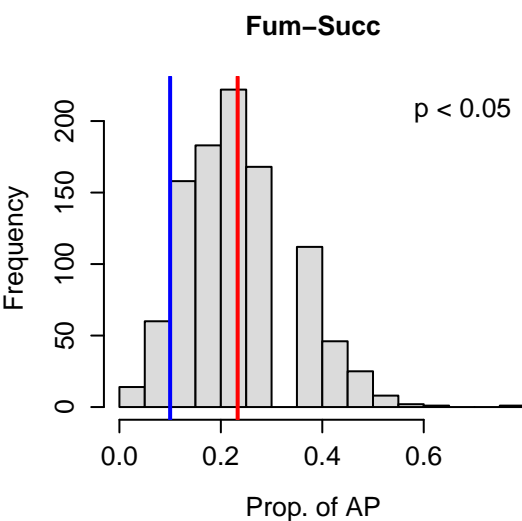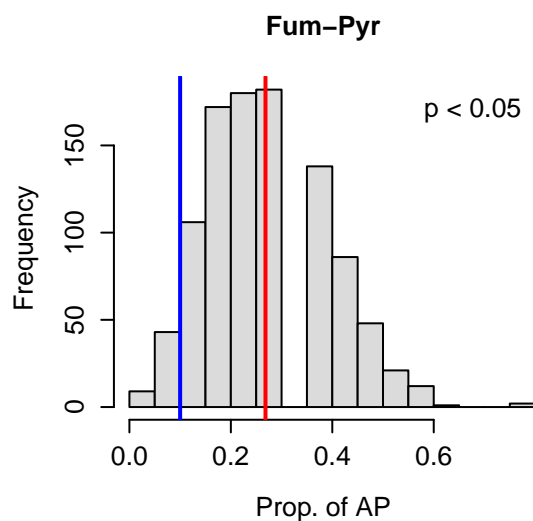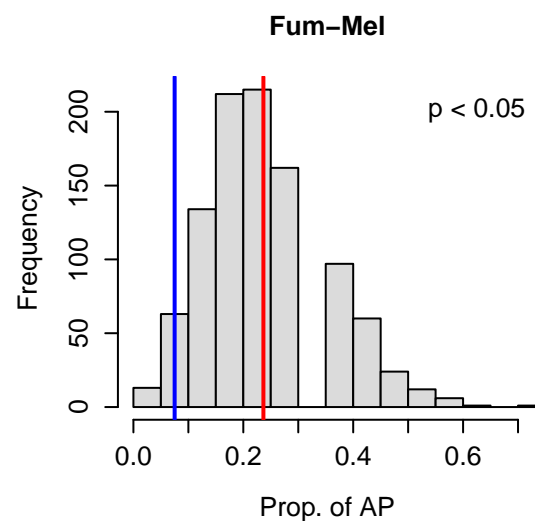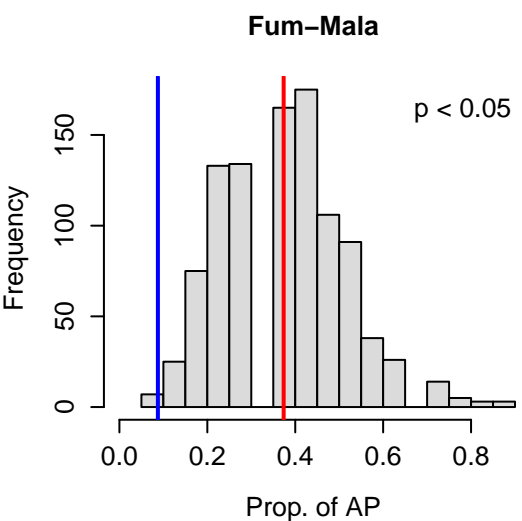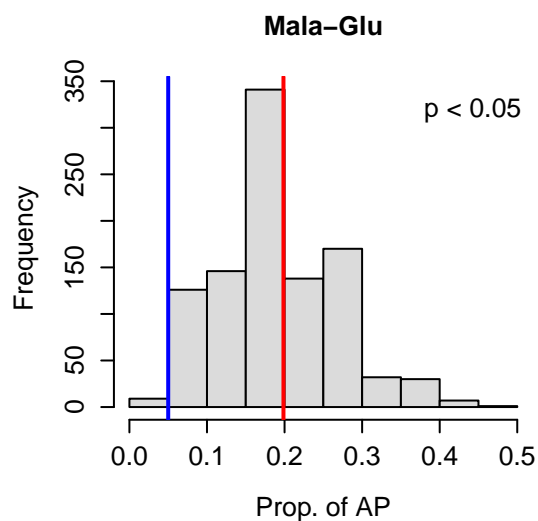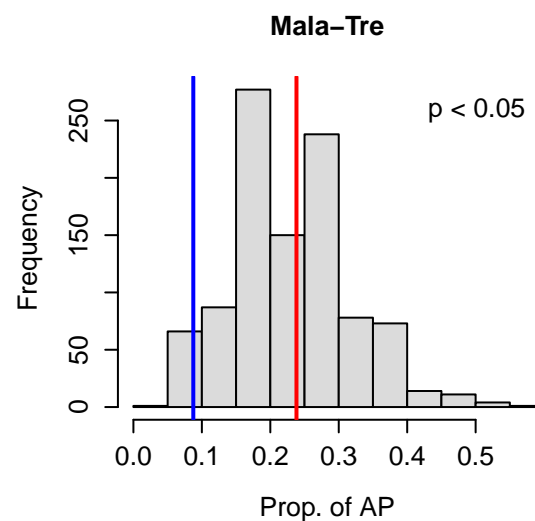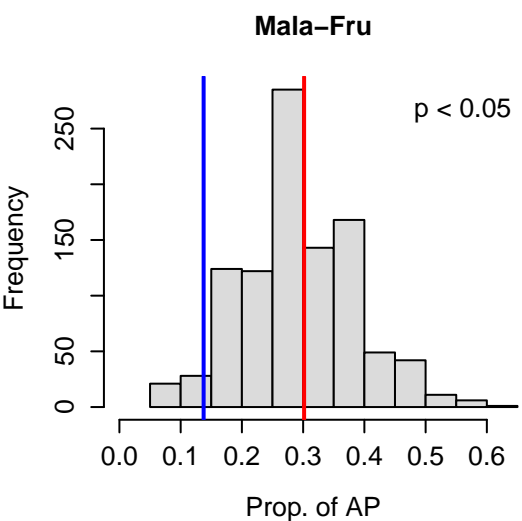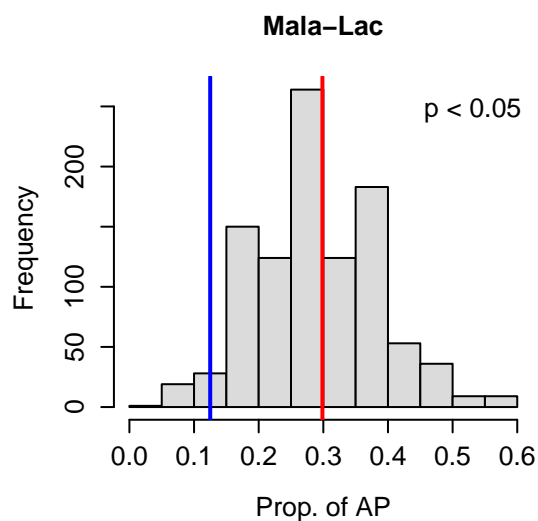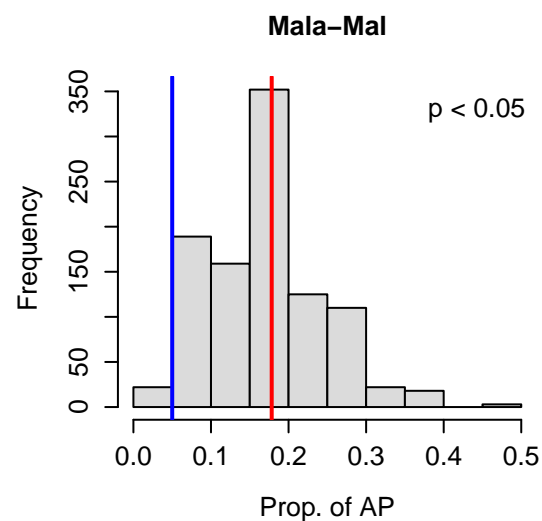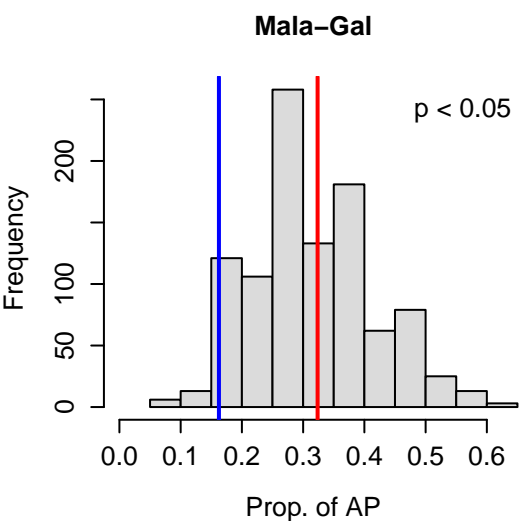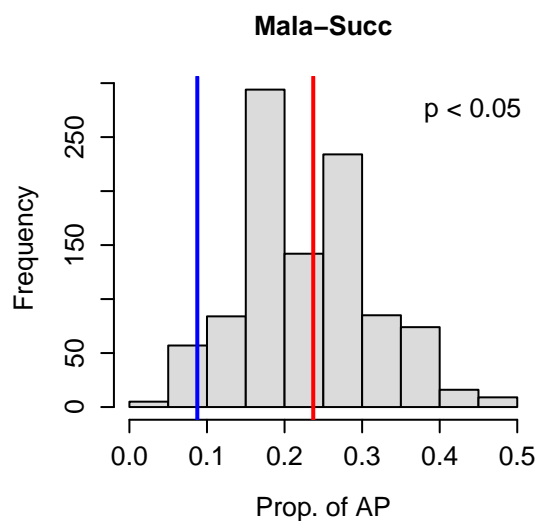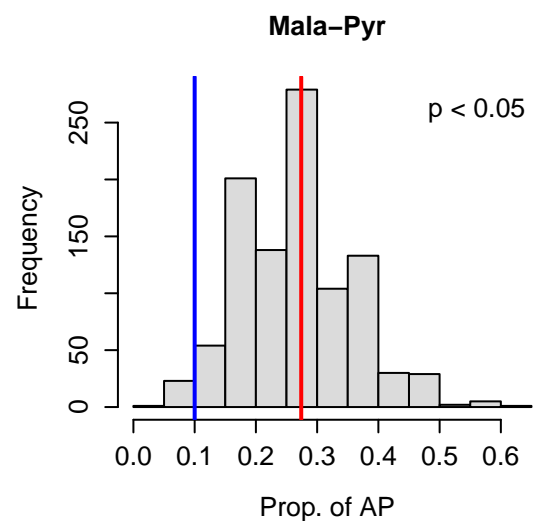

**Mala-Mel**

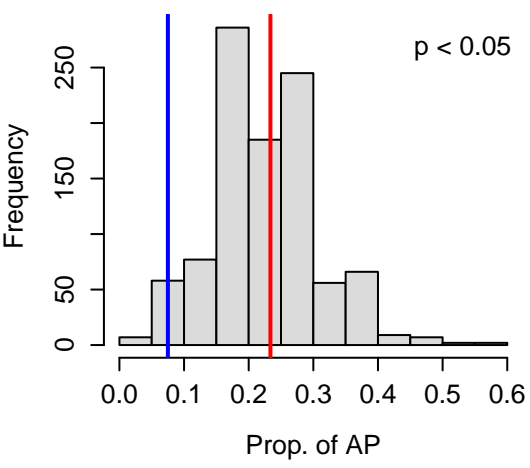

**Mala-Fum**

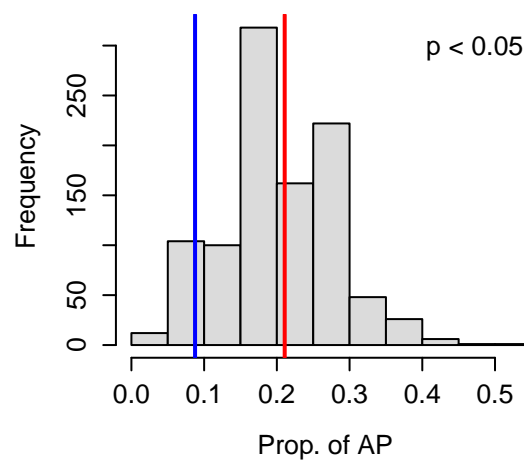

Supplement: Supplementary file 8 [file EVO-72-2202-s008.pdf]

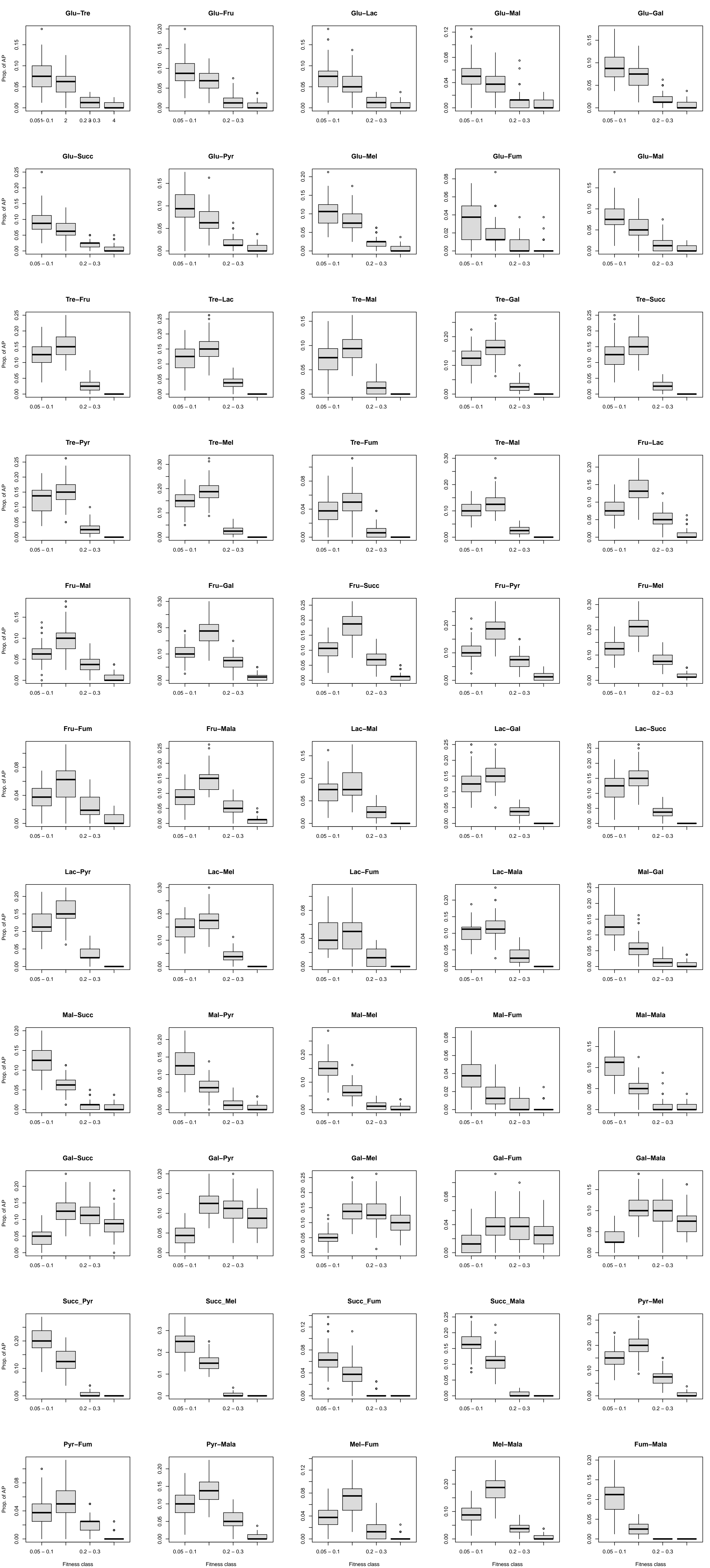

Supplement: Supplementary file 9 [file EVO-72-2202-s009.pdf]

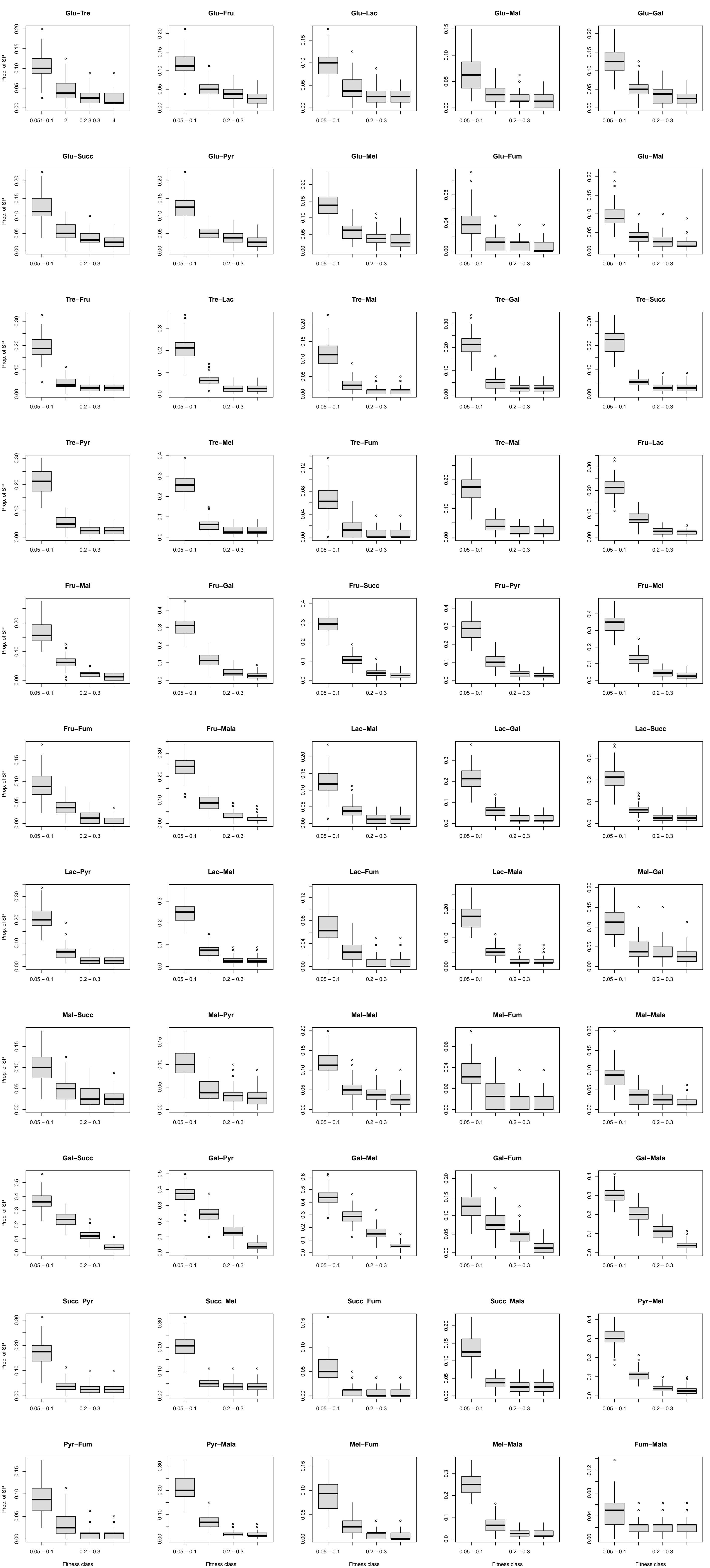

Supplement: Supplementary file 10 [file EVO-72-2202-s010.pdf]
